# Heterogeneous Off-Target Effects of Ultra-Low Dose Dimethyl Sulfoxide (DMSO) on Targetable Signaling Events in Lung Cancer In Vitro Models

**DOI:** 10.3390/ijms22062819

**Published:** 2021-03-10

**Authors:** Elisa Baldelli, Mahalakshmi Subramanian, Abduljalil M. Alsubaie, Guy Oldaker, Maria Emelianenko, Emna El Gazzah, Sara Baglivo, Kimberley A. Hodge, Fortunato Bianconi, Vienna Ludovini, Lucio Crino’, Emanuel F. Petricoin, Mariaelena Pierobon

**Affiliations:** 1 Center for Applied Proteomics and Molecular Medicine, George Mason University, Manassas, VA 20110, USA; ebaldell@gmu.edu (E.B.); khodge5@gmu.edu (K.A.H.); epetrico@gmu.edu (E.F.P.); 2 School of Systems Biology, George Mason University, Manassas, VA 20110, USA; mahaaeiou93@gmail.com (M.S.); aalsuba5@masonlive.gmu.edu (A.M.A.); emnagazzah@yahoo.com (E.E.G.); 3 Department of Mathematical Science, George Mason University, Fairfax, VA 22030, USA; goldaker@gmu.edu (G.O.); memelian@gmu.edu (M.E.); 4 Division of Medical Oncology, S. Maria della Misericordia Hospital, 06156 Perugia, Italy; baglivosara@gmail.com (S.B.); oncolab@hotmail.com (V.L.); 5 Independent Researcher, Belvedere 44, Montefalco, 06036 Perugia, Italy; fortunato.bianconi@gmail.com; 6 Department of Medical Oncology, Istituto Scientifico Romagnolo per lo Studio e la Cura dei Tumori (IRST) Istituto di Ricovero e Cura a Carattere Scientifico (IRCCS), 47014 Meldola, Italy; lucio.crino@irst.emr.it

**Keywords:** dimethyl sulfoxide effects, NSCLC cell lines, pathway activation, signal transduction, drug targets

## Abstract

Targetable alterations in cancer offer novel opportunities to the drug discovery process. However, pre-clinical testing often requires solubilization of these drugs in cosolvents like dimethyl sulfoxide (DMSO). Using a panel of cell lines commonly used for in vitro drug screening and pre-clinical testing, we explored the DMSO off-target effects on functional signaling networks, drug targets, and downstream substrates. Eight Non-Small Cell Lung Cancer (NSCLC) cell lines were incubated with three concentrations of DMSO (0.0008%, 0.002%, and 0.004% **v*/*v**) over time. Expression and activation levels of 187 proteins, of which 137 were kinases and downstream substrates, were captured using the Reverse Phase Protein Array (RPPA). The DMSO effect was heterogeneous across cell lines and varied based on concentration, exposure time, and cell line. Of the 187 proteins measured, all were statistically different in at least one comparison at the highest DMSO concentration, followed by 99.5% and 98.9% at lower concentrations. Only 46% of the proteins were found to be statistically different in more than 5 cell lines, indicating heterogeneous response across models. These cell line specific alterations modulate response to in vitro drug screening. Ultra-low DMSO concentrations have broad and heterogeneous effects on targetable signaling proteins. Off-target effects need to be carefully evaluated in pre-clinical drug screening and testing.

## 1. Introduction

The identification of targetable alterations in cancer continues to offer novel opportunities to the drug discovery process and to the development of compounds capable of directly interacting with and modulating activation of malfunctioning proteins [1,2]. Because most of these new therapeutic compounds are water insoluble hydrophobic molecules, solubilization of these drugs for in vitro screening and cell-based assays often requires the use of cosolvents, of which dimethyl sulfoxide (DMSO) represents the most widely used example [3]. While DMSO increases a drug’s solubility and, through the interaction with phospholipids [4], eases a compound’s ability to cross the cellular membrane, it also unspecifically binds to hydrophobic residues of drug targets and downstream substrates, potentially affecting their activation and function [5]. Using commercially available models commonly used for in vitro drug screening and pre-clinical testing, this work broadly explores DMSO off-target effects on functional cellular signaling networks.

While genomic alterations initiate and promote tumorigenesis, phenotypically these alterations manifest as aberrantly activated proteins and kinase-driven signal transduction events [6,7]. Within these signaling networks, kinases and downstream substrates are finely activated through post-translational modifications like phosphorylation and regulate cellular processes and gene expression profiles [7,8]. In the last few decades, compounds capable of binding altered and/or hyperactivated key nodes within these networks have opened up novel opportunities for delivering precision medicine to cancer patients [7,9]. As such, exploration of these networks is of primary importance for identifying therapeutic targets and exploring the compounds’ mechanisms of action. Given the plethora of effects DMSO has on cellular activities and macromolecules [10], discriminating between DMSO-related off-target effects and drug-specific mechanisms of action can be challenging. 

To minimize DMSO-dependent cytotoxicity [11], its effect on cell growth and differentiation rates, and altered cellular morphology, previous investigations have recommended limiting the amount of DMSO for in vitro testing to concentration < 0.1% *v*/*v* [12,13]. However, recent data have indicated that concentrations ≤ 0.1% *v*/*v* still modulate gene expression and large-scale miRNA profiles as well as tissue-specific genome-wide methylation patterns [14,15]. Thus, these data suggest that even at recommended concentrations, DMSO still affects the overall molecular landscape of target cells. However, to our knowledge the systematic and broad effects of DMSO on activation and function of therapeutically targetable signaling proteins and downstream substrates has not been reported, thus far. Given that network dynamics are an important mechanism of adaptation to cellular perturbations and resistance to targeted compounds, understanding the DMSO effect on these signaling events is of primary importance to accurately evaluate output data from in vitro preclinical testing.

Using lung cancer as a model system, this work explored the effect of ultra-low DMSO doses (8 × 10^−4^ to 4 × 10^−3^ % *v*/*v*) on the signaling network of eight commercially available cell lines commonly used for in vitro drug testing and cell-based assays. Dose- and time-dependent changes in signaling events were captured on a continuous scale using the Reverse Phase Protein Microarray (RPPA), a high throughput antibody-based assay. To fully capture off-target effects and functional deregulation of signaling networks associated with DMSO, emphasis was placed on activation levels of kinases and downstream substrates commonly targeted by FDA approved and investigational anti-cancer compounds.

## 2. Results

### 2.1. Ultra-Low DMSO Concentrations Broadly Affect the Signaling Network of NSCLC In Vitro Models

To explore DMSO-dependent off-target effects in in vitro drug testing, a panel of 8 commercially available Non-Small Cell Lung Cancer (NSCLC) cell lines was selected for molecular analysis. Cells were incubated with three different amounts of DMSO, namely 0.0008% (C1), 0.002% (C2), and 0.004% (C3) *v*/*v*, which were selected based on previously conducted cell viability studies indicating that, at large, these DMSO concentrations do not affect cells’ growth rates (Figure S1). Cell lines were incubated with the three DMSO concentrations for 5 min, as well as 1, 6, and 24 h and longitudinal biological samples were collected at each timepoint and processed by RPPA. Expression and activation levels of 187 proteins of which 137 were kinases and downstream substrates were captured at each timepoint. A non-parametric, two-tailed Kruskal Wallis rank test was performed for each cell line and within each concentration to capture changes in the expression and activation levels of the 187 measured analytes. The null hypothesis was rejected for comparisons where *p* < 0.05. A detailed list of all *p*-values that emerged from the analysis can be found in Table S1. 

**Figure 1:**
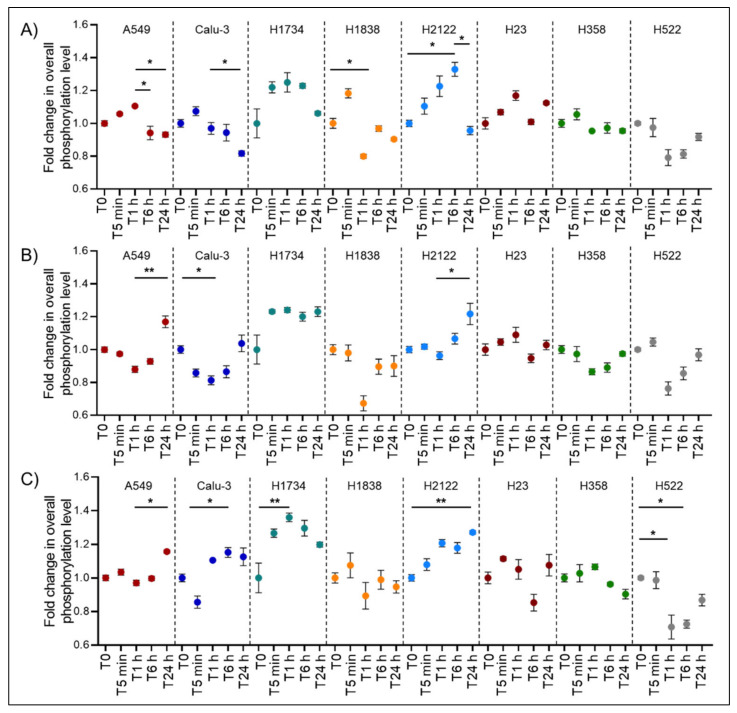
Dimethyl sulfoxide (DMSO) time- and dose-dependent changes of overall phosphorylation levels in eight lung cancer in vitro models. Broad change in protein phosphorylation levels was measured as aggregate changes of the Reverse Phase Protein Array (RPPA) intensity values across 125 post-translationally modified kinases and downstream substrates within each cell line. Tallied values for each cell line were normalized to the baseline (T0) of the corresponding DMSO concentration and expressed as fold change of the untreated values (C1 Panel **A**, C2 Panel **B**, and C3 Panel **C**, respectively). For each timepoint and DMSO concentration, technical replicates were collected (*n* = 3) and mean and standard error of the mean are shown. Adjusted pair-wise comparisons that retained statistical significance (* *p* < 0.05; ** *p* < 0.01, respectively) are shown for each cell line.

To explore the effect of DMSO on functional signaling networks and post-translationally modified proteins, broad changes in phosphorylation levels of 125 kinases and downstream substrates were first evaluated (Figure 1). Fold-change in the overall phosphorylation levels ranged between 0.58 and 1.4. As expected, cell lines presented different patterns of response to DMSO across timepoints. For example, DMSO induced an overall downregulation of signaling activity in some cell lines (Calu3 *p* = 0.01, 0.04, and 0.03 for C1, C2, and C3, respectively), while it promoted signal transduction events in others (e.g., H2122 *p* = 0.04, 0.03, and < 0.01 for C1, C2, and C3, respectively). The H2122 and H1734 lines were the most affected cell lines and presented a nearly 40% increase in the overall phosphorylation levels; however, the magnitude of change varied greatly across timepoints. On the other hand, the H358 and H23 cell lines were the least affected models within and across concentrations (*p* > 0.05). However, datapoints trended in similar directions across different concentrations of DMSO, suggesting that DMSO induces similar biological effects regardless of its amount.

**Figure 2:**
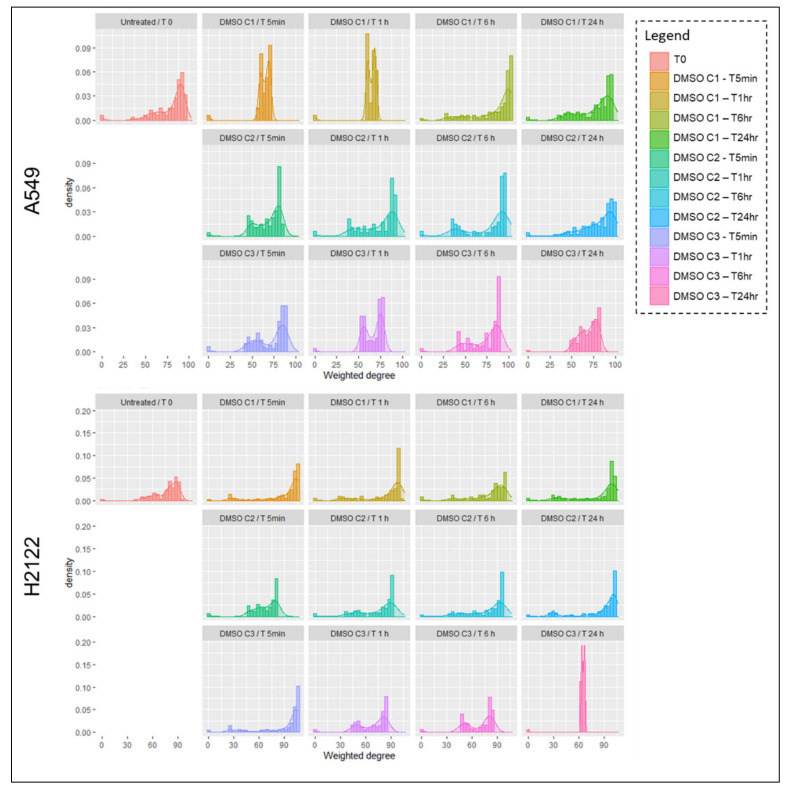
Histograms and kernel density plots representing weighted Pearson’s correlation coefficients for each DMSO dose across timepoints for A549 and H2122. Weighted Pearson’s correlation coefficients were calculated for paired proteins across all measured analytes and the degree of distribution of pair-wise interconnections between analytes is shown for each cell lines, DMSO concentration, and timepoints.

In contrast, DMSO had an inhibitory effect on phosphorylation events in the H522 cells, especially 1 and 6 h after the administration of DMSO. Similarly, a 20% to almost 40% reduction in phosphorylation events was detected after 1 h of incubation in the H1838. The ability of DMSO to change signaling network activities, especially at earlier timepoints after incubation, may be of relevance when testing targeted compounds as these changes may alter a compound’s activity, its ability to engage with its target(s), and its effect on downstream substrates. To further explore the effect of DMSO on the overall signaling network, weighted Pearson’s correlation coefficients were calculated and their distribution was represented as histograms and kernel density plots (Figure 2; Figure S2). This approach allowed us to capture the degree distribution of pair-wise interconnections between the measured analytes, thereby providing a broad overview on how the overall signaling network adapted to DMSO. As expected, the degree of interconnection between the analytes varies not only between cell lines, but also across timepoints and DMSO concentrations. For example, network interactions in the A549 changed dramatically 5 and 60 min after incubation at the C1 DMSO concentration. However, at 24 h the overall distribution of these interconnections was similar to the baseline values (Figure 2).

In contrast, interconnection in the H358, H2122, and H522 never recovered baseline levels within the 24 h period (Figure 2; Figure S2). However, interconnection showed a similar distribution at 24 h across DMSO concentrations in some models (e.g., H2122, A549, between C1 and C2, H522 between C2 and C3) (Figure 2; Figure S2). Overall, these data suggest that DMSO affects post-translational modifications in a time and cell line dependent manner. Given the ability of DMSO to modulate a cell’s overall signaling activities, the selection of appropriate DMSO concentrations and controls in in vitro experiments should be carefully evaluated to minimize unwanted up- or down-regulation of drug targets and downstream substrates.

### 2.2. Ultra-Low DMSO Concentrations Have Heterogeneous and Cell Line Dependent Effects on Signaling Molecules in NSCLC Models

**Figure 3:**
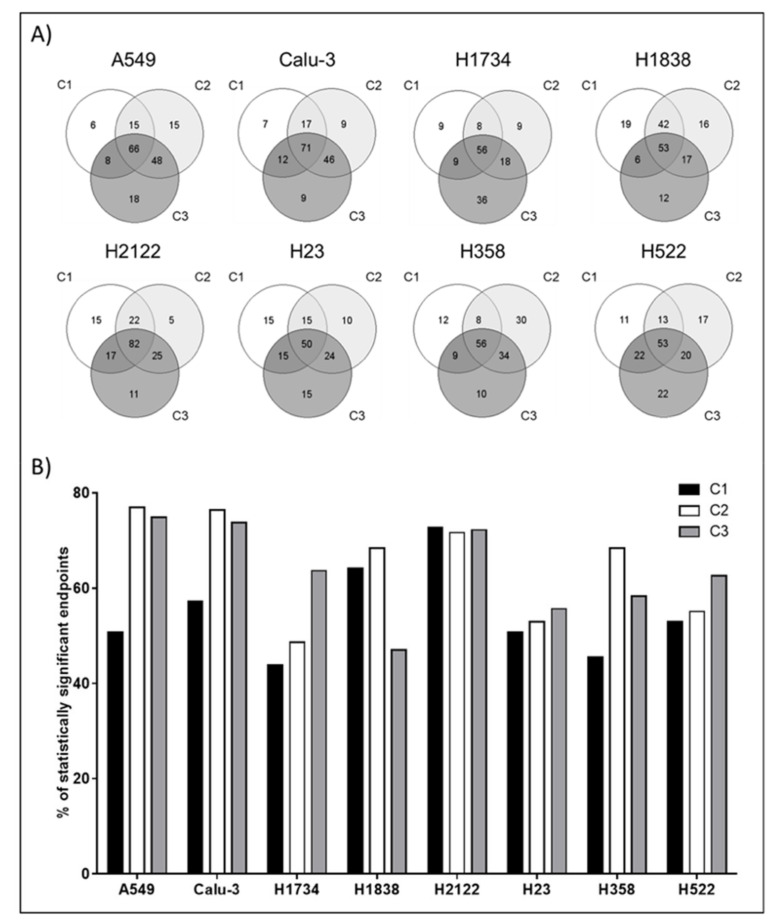
For each cell line and DMSO concentration (C1 = 0.0008%, C2 = 0.002% and C3 = 0.004%, respectively), a two-tailed Kruskal Wallis rank test was performed to capture changes in protein expression/activation across timepoint. Technical replicates (*n* = 3) were collected for each observation. Number and percentage of proteins that reached statistical significance in each DMSO concentration are reported. Venn diagrams illustrate unique and shared proteins across DMSO concentrations that reached statistical significance within each cell line (Panel **A**). Bar graphs show the percentage of proteins and phosphoproteins that were statistically different in each cell line and at each DMSO concentration (Panel **B**).

To evaluate the DMSO effect on individual proteins, a multiple-group comparison across timepoints was calculated for each cell line. Of the 187 proteins measured, 185 (98.9%) were statistically different in at least one cell line for the DMSO concentration C1, while the number of statistically different endpoints increased to 186 (99.5%) in C2, and to 187 (100%) C3 (Figure S3). On a cell line-by-cell line basis, the magnitude of the DMSO effect was highly heterogeneous and less than half of the analytes that reached significance were shared across all three DMSO concentrations within one cell line (Figure 3A). The H1734 cells were the least affected cell line and significant differences (Kruskal Wallis test *p* < 0.05) were found in less than half of the measured analytes in C1 and C2 (43.9% and 48.7%, respectively). However, the proportion of significant endpoints reached 63.6% at the highest DMSO concentration (Figure 3B, Table S1). Only 64 (34.2%) proteins were shared between C1 and C2 and 56 (29.9%) of those were also found significantly altered in C3 (Figure 3A). The H2122 cell line emerged as the model most affected by DMSO with 135 (72.2%) proteins reaching statistical significance in the C3 condition followed by 134 (71.7%) and 136 (72.7%) in C2 and C1, respectively (Figure 3B and Table S2). A total of 82 proteins were shared across the three conditions (43.9%) (Figure 3A). 

**Figure 4:**
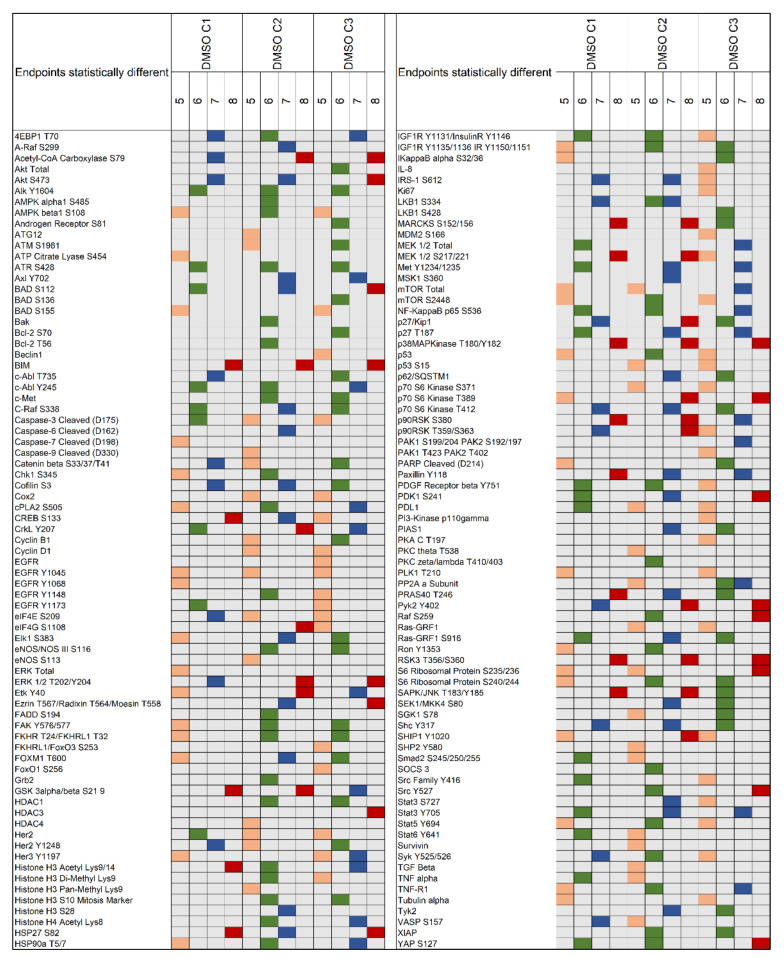
For each cell line and DMSO concentration (C1 = 0.0008%, C2 = 0.002%, and C3 = 0.004%, respectively), a two-tailed Kruskal Wallis rank test was used to capture changes across timepoints (*n* = 5). Proteins that emerged as statistically significant in five (orange), six (green), seven (blue), or all eight (red) cell lines are shown in the matrix. A total of 150 proteins met the criteria and are displayed.

**Figure 5:**
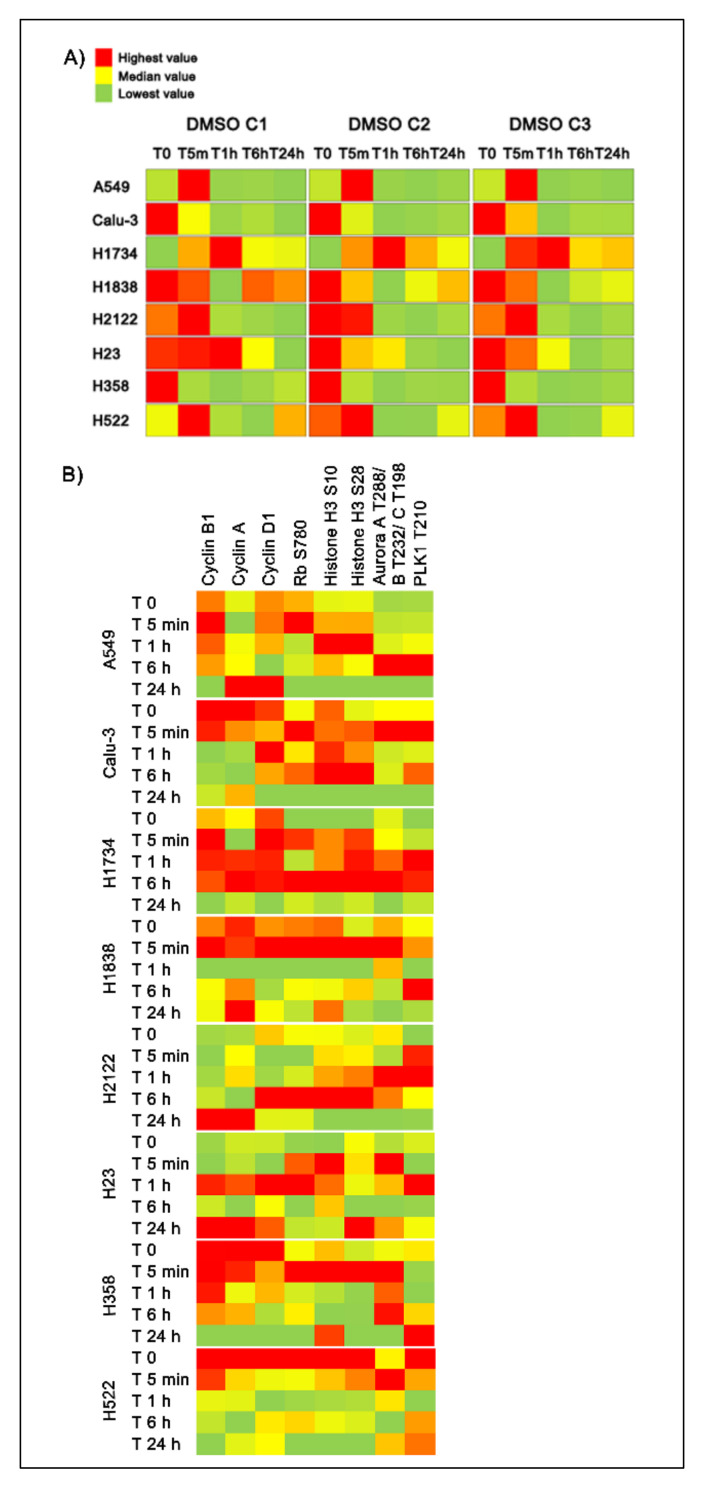
Heatmaps displaying dose- and time-dependent changes in ERK 1/2 activation measured as phosphorylation of the T202/Y204 residues (Panel **A**) along with expression/activation levels of proteins involved in the cell cycle regulation (Panel **B**). Heatmaps capture average values (*n* = 3) across five timepoints for the three DMSO concentrations within each cell line using a red (highest) to green (lowest) scale.

**Figure 6:**
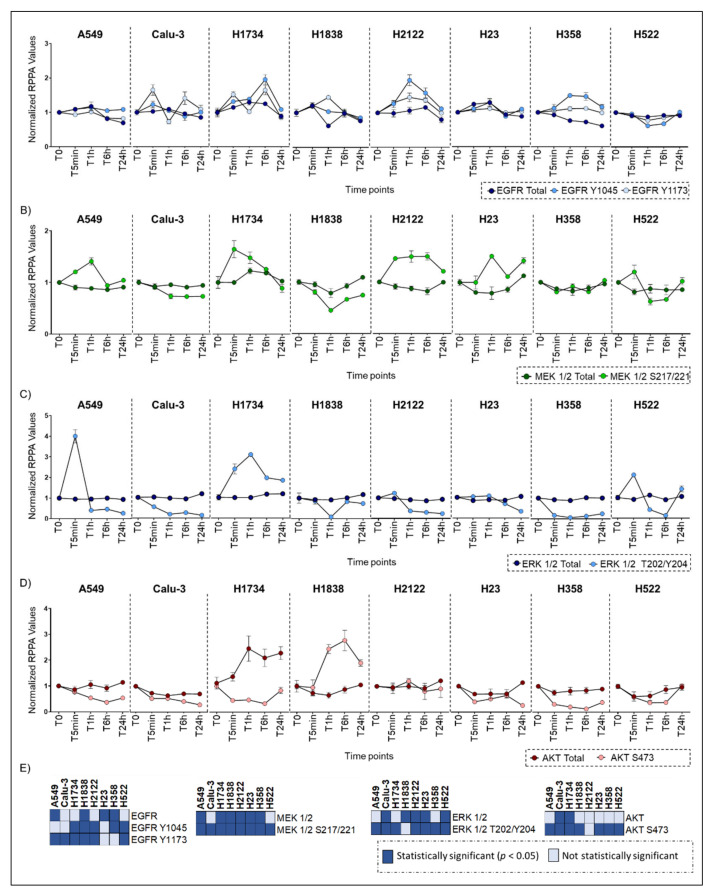
Low-dose DMSO effects on the expression and activation of selected proteins across timepoints (*n* = 5). Line plots display the heterogeneous effects of the solvent on selected protein across eight NSCLC cell lines at the DMSO C1 concentration. EGFR (Panel **A**), MEK 1/2 (Panel **B**), ERK 1/2 (Panel **C**), and AKT (Panel **D**) are displayed. Mean and standard error of the mean for the technical replicates (*n* = 3) are shown. Changes that emerged as statistically significant across timepoints using the Kruskal-Wallis test are shown in (Panel **E**).

Of the 187 proteins evaluated, 86 (46.0%) were significantly different in more than 5 cell lines in C1 followed by 120 (64.2%) in C2 and C3 (Figure 4; Figure S3). Amongst post-translationally modified key signaling nodes, phosphorylated ERK 1/2 T202/Y204 was found statistically significant in 7 of the 8 lines in C1 and in all lines in C2 and C3 (Figure 4; Figure S3). However, trends and magnitude of changes in ERK 1/2 activation varied significantly across cell lines and times (Figure 5A; Figure 6C). For example, in C1 ERK 1/2, activation was maintained in the H1838 across timepoints with the exception of a drop at the 1-h mark. A significant increase in the phosphorylation of residues T202/Y204 was detected after 5 min incubation in the A549 and H522 cell lines with the greatest effect on the A549 line that never recovered ERK 1/2 baseline activation levels afterward. 

In the Calu-3 and H358 models, ERK 1/2 activation was significantly reduced throughout the incubation period, while in the H1734 line, ERK 1/2 activation levels progressively increased by 2 to 3 times and never recovered to baseline levels. Trends in ERK 1/2 activation in C2 and C3 followed activation patterns resembling those of C1 (Figure 5A).

Activation patterns across time also varied from cell line to cell line for AKT, another key regulator of a complex signaling axis. DMSO induced downregulation of AKT S473 in six of the eight cell lines and only the H522 fully recovered after 24 h of incubation (Figure 6D). Increased activation of AKT was detected across all timepoints in the H1838 model, while AKT was only marginally affected in the H2122 cell line (Figure 6D). While changes in ERK 1/2 and AKT expression and phosphorylation levels were significant across lines, the magnitude of these fluctuations across timepoints was less prominent for the expression than for the activation levels. Thus, the effect of DMSO on ERK 1/2 and AKT activity was most likely independent from changes in its overall level of expression (Figure 6C–E). Similar trends were also observed for ERK 1/2 upstream signaling molecules including the receptor tyrosine kinase (RTK) EGFR and MEK1/2 (Figure 6A,B).

**Figure 7:**
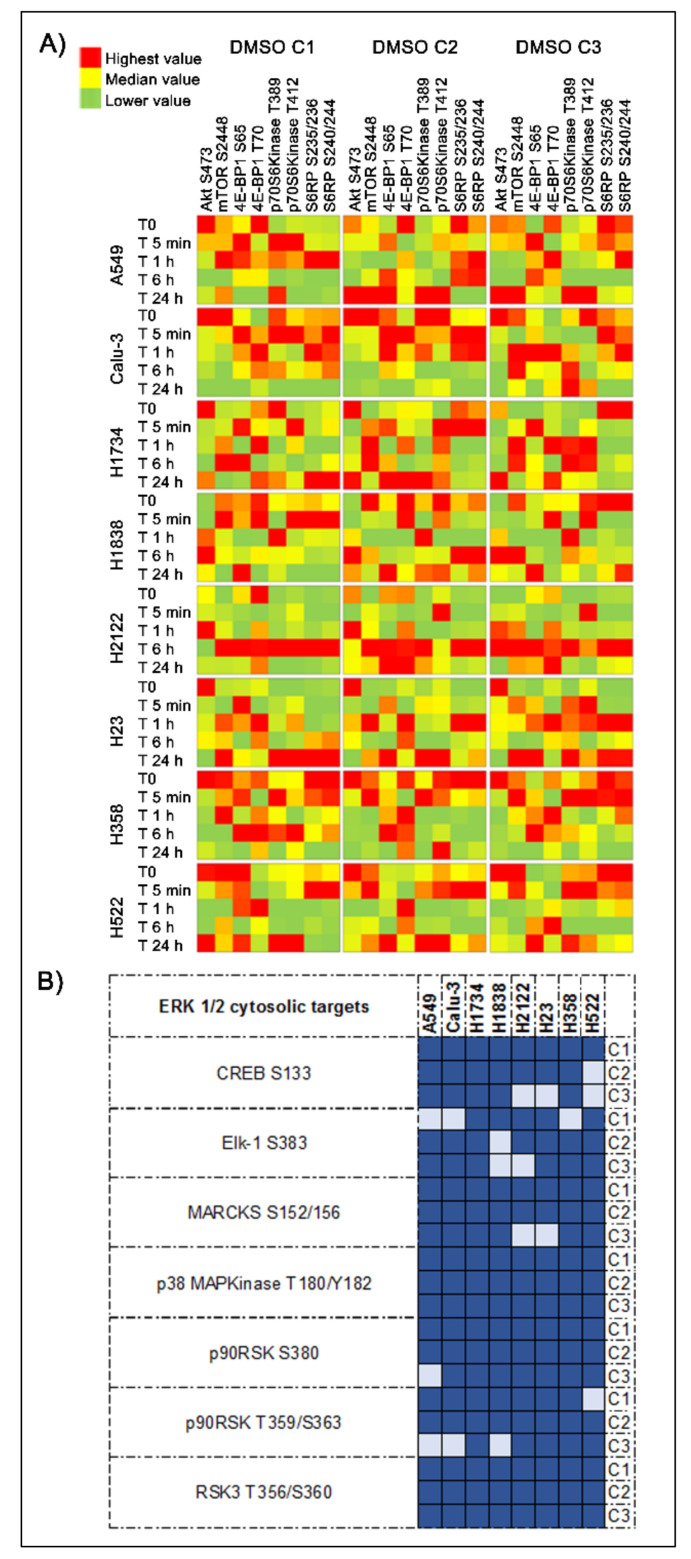
Changes in AKT-mTOR signaling and in the activation of ERK 1/2 downstream substrates induced by the DMSO. Heatmaps display average values (*n* = 3) for proteins belonging to the AKT-mTOR signaling pathway across timepoints are shown using a red (highest) to green (lowest) scale (Panel **A**). Matrixes illustrate the number of cell lines in which significant differences were detected (dark blue) in ERK 1/2 cytosolic targets and regulated transcription factors across the three DMSO concentrations. (Panel **B**).

To further capture DMSO dependent effects on signaling networks, we evaluated activation levels of ERK 1/2 and AKT downstream substrates across timepoints and concentrations (Figure 7A,B). Activation levels of the ERK 1/2 cytosolic targets MARCKS S152/156, p38MAPK T180/182, and RSK3 T365/S360 and its target transcription factors Elk-1 S383 and CREB S133 were also found to be significantly altered across models and DMSO concentrations (Figure 7A,B). For example, the two-tailed Kruskal Wallis rank test examining changes in the phosphorylation levels of CREB at the residue S133 was statistically significant (*p* < 0.05) across timepoints for all eight cell lines in C1, seven cell lines in C2, and five cell lines in C3 (Table S1), suggesting that changes in the activation level of this transcription factor are highly affected by DMSO and of biological relevance. However, while it is well-known that changes in ERK 1/2 activity vary across different phases to the cell cycle, [16], in our models ERK 1/2 phosphorylation levels did not mimic expression or activation patterns of cell cycle regulators (e.g., expression level of Cyclin A, B1, and D1; phosphorylation levels of Rb, Histone H3, Aurora Kinase, etc.) (Figure 5B). These observations suggest that most changes captured by this analysis do not merely mirror physiological cellular activities. 

Similar trends were also observed for AKT downstream substrates across timepoints and concentrations (Figure 7A). While patterns of AKT activation were slightly maintained across DMSO concentrations, its downstream substrates presented with less predictable behaviors. However, when multiple phosphorylation sites were measured for a given protein, in most cases similar patterns were detected across phosphorylation sites (e.g., S6RP S235/236 and S240/244; p70S6 T389 and T412 kinase, etc.) (Figure 7A). Whether DMSO concomitantly affects multiple members of these cascades or it modulates the activity of key nodes within these signaling networks that then aberrantly regulate downstream activities, and consequently cellular functions and gene expression profiles, is outside the scope of this work and needs to be evaluated in future investigations.

### 2.3. Ultra-Low DMSO Effects on Signal Transduction Cascades Are Time- and Model-Dependent

Pathway enrichment analysis, using the Reactome Knowledgebase software [17,18], was then performed to evaluate DMSO associated network dynamics across the eight models (Table S3). While by nature the RPPA analysis requires an a priori selection of the target analytes, only proteins that reach statistical significance were imputed for this analysis, which allowed us to identify pathways uniquely enriched in each cell line. As expected, the MAPK signal transduction cascade and the PI3K/AKT signaling pathway were enriched across cell lines, however the degree of over-representation of these pathways, and consequently their ranking and relevance, varied greatly across models (Figure S4). For example, enrichment of the MAPK family cascade included a variable number of proteins across cell lines, ranging from 19 to 25 nodes (Figure S4A). Only the A549 and Calu-3 models were enriched for multiple pathways involved in the vertical regulation of the ERK 1/2 signaling including RTKs like HER2, and regulatory signaling (e.g., Shc1 events in HER2 signaling) along with RAF activity (e.g., RAF/MAP kinase cascade) (Figure S4A). Half of the cell lines, namely the A549, Calu-3, H1838, and H2122, showed enrichment for proteins involved in apoptosis (Table S3). Although pro-apoptotic signaling and changes in post-translationally modified caspase levels were detected in different models after the incubation with DMSO, these changes had a minimal impact on the overall cell viability. A potential explanation for this observation may be given by the concomitant activation of counterbalancing ERK-driven stress responses and AKT-driven pro-survival. Indeed, enrichment for phosphatidylinositol (3,4,5)-trisphosphate (PIP3) and AKT signaling was found across cell lines. However, the number of proteins found in these enriched networks varied greatly from 18 to 27 nodes across cell lines (Figure S4B). Of interest, enrichment for the downstream mTOR signaling was identified only for the H522 and H2122 cell lines, while the Calu-3, H1734, H1838, and H358 models were enriched for interconnected proteins involved in the negative regulation of the PI3K/AKT network. Overall, these data suggest that signaling molecules and networks are differentially affected by DMSO in different cell lines suggesting that when conducting drug sensitivity studies, the effect of DMSO should not be considered as an evenly distributed variable across models.

### 2.4. DMSO Effect on Signal Transduction Pathways May Affect Cell Lines’ Responses to Treatment 

**Figure 8:**
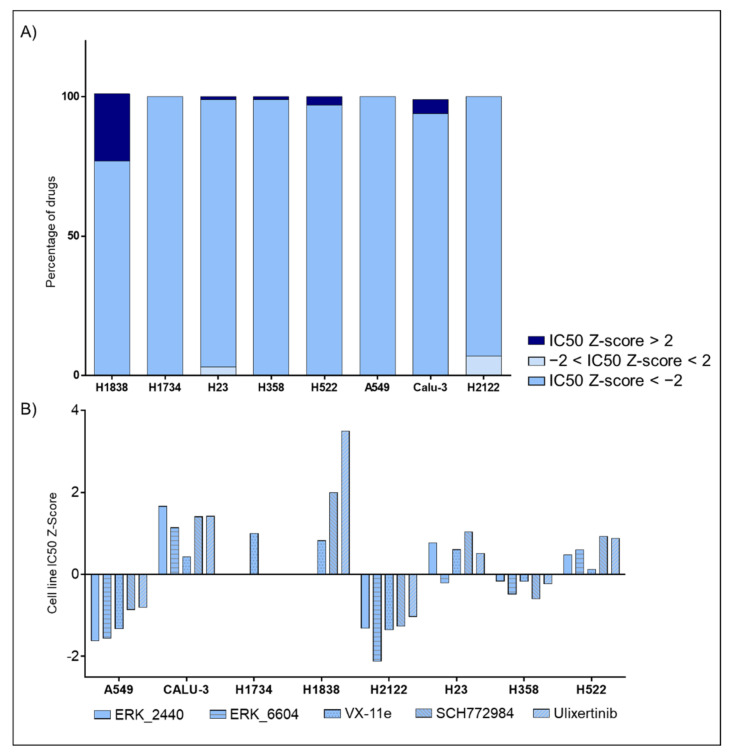
Low-dose effects of DMSO on response to targeted compounds and chemotherapeutic agents. Bar graphs capturing drug susceptibility across eight NSCLC cell lines using the IC50 Z-scores provided by the GDSC project (Panel **A**). GDSC-derived data capturing responses to ERK inhibitors across lines (Panel **B**).

Finally, using the Genomics of Drug Sensitivity in Cancer (GDSC) database (https://www.cancerrxgene.org, accessed on 1 February 2021), we evaluated broad responses to targeted agents and standard chemotherapeutic compounds across our eight cell lines. Drug susceptibility information was collected on average for 180 compounds with the exception of the H1838 and H1734 (101 and 29 compounds, respectively). For each compound and cell line, the GDSC database provides IC50-based Z-scores. Cell lines with Z-scores > +2 are considered resistant to treatment while those with Z-scores < −2 were defined as sensitive. Of interest, H2122, the cell line most affected by DMSO in our analysis, showed the greatest sensitivity across all compounds (12 or 7% of all tested compounds), followed by H23 (2.8%) and Calu-3 (0.5%) (Figure 8A). The H2122 cells were resistant to none of the 180 compounds tested by GDSC program. Given the wide effect of DMSO on ERK 1/2 activation across the eight models analyzed, response to ERK inhibitors were then retrieved from the GDSC database. Of interest, cell lines where DMSO caused downregulation of ERK 1/2′s activity were associated with increased sensitivity to the targeted compounds (Figure 8B). In contrast, cells with unaltered or increased ERK 1/2 activation after incubation with DMSO had higher level of resistance to the compounds. For example, the H1838 line, the only model where ERK 1/2 activation did not emerge as significant across time in the C1 concentration, presented with the highest level of resistance. On the other hand, A549, H2122, and H358 cells, where significant reduction in ERK 1/2 was detected after the administration of DMSO (*p* values < 0.01), were amongst the models with the highest sensitivity to different anti-ERK compounds. The Calu-3 cells were the only exception to this pattern. Taken together, these data indicated that the DMSO effects on signaling events and drug targets is highly heterogeneous. Cell-dependent response to DMSO may differentially affect activation levels of drug targets and downstream substrates across cell lines and should be carefully evaluated in drug screening and in vitro testing.

## 3. Discussion

To our knowledge, this is the first report exploring broad off-target effects of DMSO on protein signaling networks with a focus on biochemically interconnected, post-translationally modified drug targets and downstream substrates. With a growing number of targeted compounds entering the market every year, discerning the effects of experimental variables from true drug-associated effects is of primary importance to improve the drug discovery process and pre-clinical testing. While DMSO remains the most commonly used cosolvent for water insoluble compounds in pharmacological and toxicological studies, the concentration at which it is used is often unreported [19]. Subsequently, the biological off-target effects and DMSO’s ability to modulate expression and activation of drug targets and their downstream substrates, and consequently gene expression profiles, can be overlooked from a biological perspective. 

It has been previously documented that DMSO is a non-inert substance with significant effect across macromolecules and subsequently cellular functions and molecular profiles [13,14,20,21,22]. A few examples are DMSO’s ability to alter membrane phospholipid composition [23,24,25], DNA conformation, and epigenetic profiles [3,10,26,27]. From a protein perspective, previous analyses have indicated that DMSO can directly modulate proteins’ functional and structural stability [28], reduce protein complexes’ binding affinities, affect the stabilization of protein quaternary structure and noncovalent interactions between protein and ligands, and promote proteins’ degradation [1,3,29]. However, these alterations exhibit arbitrary behaviors and unpredictable patterns across models, tissue types, and duration of the incubation [14,30].

In vitro studies and biochemical assays have previously reported that exposure to DMSO can promote phosphorylation of kinases on multiple phospho-epitopes [31,32,33]. Earp and colleagues have also reported that treatment with DMSO, although at much higher concentrations than the one used in this analysis (10% *v*/*v* ), selectively induces phosphorylation of tyrosine residues on the EGF receptor in crude membrane fractions of murine erythroleukemia cells [34]. In line with these results, our analysis showed that DMSO strongly influences the post-translational state of druggable membrane and cytosolic kinases and their signal transduction activity, even when DMSO is used at concentrations which are significantly below previously recommended amounts. However, the trend and magnitude of these activation patterns are highly heterogeneous across cell lines and timepoints. Thus, DMSO directly affects baseline activation levels of many targets of FDA-approved and investigational agents following unspecific behaviors. 

Activation of ERK 1/2, a central node of the proliferative MAPK pathway and a crossroads between signal transduction networks, is a good example of these diverse adaptation mechanisms as each of the eight models analyzed in this work showed distinct patterns of ERK 1/2 activation and recovery after 24 h of incubation with DMSO. These trends appeared to be independent from expression and activation of cell cycle regulatory molecules or changes in ERK 1/2 overall expression levels, suggesting that DMSO directly affects ERK’s signaling activity. Similarly, Cataldi and colleagues have previously demonstrated that incubation with DMSO for 24 h induces PI3K activation, while the overall expression of the protein remains unaffected within this timeframe [35]. As expected, pathway enrichment analysis identified the pro-survival PI3K/AKT signaling pathway as one of the most frequently affected networks by DMSO. However, the level of activation of this signal transduction cascade varied from model to model. Apoptosis, on the other hand, was affected in only half of the analyzed models. Based on previous reports, DMSO can induce apoptosis by directly modulating the activity of members of this signaling pathway including cleavage of PARP-1 or promote mitochondrial-mediated, Caspase-3- and 9-dependent apoptosis through the down-regulation of members of the Bcl-2 family [19,36]. Taken together, these data indicate that DMSO alters druggable biochemically linked signaling transduction molecules in a heterogeneous way. Thus, the effect of DMSO on individual drug targets and downstream substrates should be carefully evaluated when conducting drug screening and testing. 

Although this work did not directly explore the effect of DMSO on response to treatment(s), data from the “The Genomics of Drug Sensitivity in Cancer” (GDSC) database have provided some insights on their potential interactions. The H2122 cell line, a model whose signaling network was profoundly affected by DMSO, emerged as the most sensitive model across compounds (IC50 Z-score <-2 for 12 of the 180 drugs). In addition, H2122 cells were not resistant to any of the 180 compounds tested. When emphasis was placed on ERK inhibitors exclusively, similar trends were observed. Specifically, cell lines where DMSO induced significant ERK 1/2 downregulation were overall more sensitive to these targeted compounds, while models where ERK 1/2 phosphorylation was unaltered or increased by DMSO showed higher levels of resistance, with only one exception. Although these observations are based on a small number of cell lines, and on a relative short time frame (only up to 24 h), a clear trend was detected between overall drug susceptibility and DMSO effect on cellular signaling networks. This data may have important implications for drug screening and pre-clinical studies, as DMSO may affect drug targets baseline activation levels and susceptibility to treatment. 

While we specifically designed this analysis to capture short-term adaptation mechanisms associated with DMSO, a few limitations of our work should be addressed. First, this study analyzed in vitro models established from lung malignancies only. Whether these observations hold true across tissue types should be validated in independent analyses. Previous investigations have indicated that the magnitude of the DMSO effects on phospholipids, DNA, RNA and proteins varies from cell type to cell type [3,26,27]. Thus, validating these results on a larger number of models and across tumor types may offer a more comprehensive understanding of the unspecific effects of the solvent in drug screening and in vitro testing. Second, our analysis only captured changes in drug targets’ activities within a 24 h timeframe. While these changes were significant and rarely did our models recover baseline activation levels of specific targets and of interactions patterns, this data do not provide direct insights on the long-term effects of DMSO. However, given that most studies exploring drugs’ mechanisms of action often measure changes in molecular profiles within a 24–72 h incubation period, our data provide valuable understandings on how DMSO can unwittingly affect in vitro drug testing. Devising novel methods able to account for these off-target effects may improve the drug sensitivity testing process and lead to the selection of compounds with a higher potential of achieving clinical benefit. For example, understanding if these changes are transitory and cells have the ability to recover baseline activation levels after longer exposure to DMSO, may lead to the development of protocols and standard procedures where cells are pre-sensitized with the solvent. As an alternative, prescreening of in vitro models may also help identify cell lines where the drug target(s) of interest and downstream substrates are only marginally affected by DMSO and develop analytical tools to account for these unspecific effects. For example, this work mostly captured broad changes across timepoints within each cell line. However, given the large number of datapoints analyzed, adjusted pair-wise comparisons within a given concentration of DMSO were not examined. However, identifying timepoints in each model that are most affected by DMSO may help optimize the experimental designs and minimize the effect of DMSO on signaling transduction events. Lastly, cross-validating significant findings with alternative solvents may also represent a possible solution for capturing the true effect of a targeted compound on the target cells. Taken together, these results suggest that DMSO, even in minuscule doses, can produce detrimental effects on proteins’ signaling networks, drug targets, and downstream substrates’ activity. These heterogeneous, unpredictable, and model-dependent effects should be carefully evaluated when designing and implementing in vitro drug screenings for pre-clinical testing. Limiting the amount of DMSO preventively, even to amounts that are below current recommendations, may improve the accuracy and reliably of in vitro testing.

## 4. Materials and Methods

### 4.1. Cell Cultures 

A panel of eight human non-small cell lung cancer cell lines including A549, H1734, H1838, H2122, H21, H358, and H522 were used in this analysis. Calu-3 were purchased from AddexBio (San Diego, CA, USA), and the remaining seven lines were obtained from American Type Culture Collection (ATCC, Manassas, VA, USA). Cell lines were grown following manufacturer’s recommendations. As suggested by the manufacturer, cell lines were grown in media (F-12K for A549; RPMI-1640 for H1734, H1838, H2122, H21, H358 and H522, and Eagle’s MEM medium for Calu-3; ATCC, Manassas, VA, USA) supplemented with 10% fetal bovine serum (FBS) (ATCC, Manassas, VA, USA) at 37 °C and 5% CO_2_ atmosphere. Cells were passaged using trypsin/EDTA (ATCC, Manassas, VA, USA) and sub-cultured at a ratio ranging between 1:3 and 1:10 depending on each cell line’s growth rate.

### 4.2. Cell lines Treatments for Functional Signaling Analysis 

Cells were seeded in technical replicates (*n* = 3) in a 6-well plate and cultured to 80% confluency. Each cell line was treated with three ultra-low DMSO concentrations (ATCC, Manassas, VA, USA) 0.0008% (C1), 0.002% (C2) and 0.004% (C3) respectively for 5 min, 1 h, 6 h, and 24 h (5min, T1 h, T6 h, and T24 h, respectively). Baseline untreated control samples were also collected along with the longitudinal samples. At the end of the incubation period, cells were washed twice with PBS (Invitrogen Life Technologies, Carlsbad, CA, USA) and lysed in TPER (Thermo Fisher Scientific, Waltham, MA) supplemented with 300 mM sodium chloride and a cocktail of protease and phosphatase inhibitors to prevent protein degradation and dephosphorylation including: 1 mM sodium orthovanadate (Sigma Aldrich, St. Louis, MO, USA); 200 mM PEFABLOC (Roche, Basel, Switzerland); 5 µg/mL Aprotonin (Sigma Aldrich, St. Louis, MO, USA), 5 µg/mL Pepstatin A (Sigma Aldrich, St. Louis, MO, USA) (prepared at 1.0 mg/mL in a solution of 10% acetic acid and 90% methanol) and 5 µg/mL Leupeptin (Sigma Aldrich, St. Louis, MO, USA) [37]. Protein concentrations were assessed using the Coomassie (Bradford) Protein Assay Kit (Thermo Fisher Scientific, Waltham, MA, USA) following the manufacturer’s instructions. Lysates were first diluted to 1 µg/µL concentration in TPER and subsequently brought to a final concentration of 0.5 µg/µL in 2X Tris-Glycine SDS Sample buffer (Invitrogen Life Technologies, Carlsbad, CA, USA) supplemented with 5% 2-mercaptoethanol (Sigma Aldrich, St. Louis, MO, USA). Samples were boiled for 8 min and stored at -80 °C prior to array construction.

### 4.3. Reverse Phase Protein Microarray 

Cell lysates were immobilized in technical triplicates onto nitrocellulose coated glass slides (Grace Bio-labs, Bend, OR, USA) using an Aushon 2470 arrayer (Quanterix, Billerica, MA, USA) equipped with 185 μm pins. Reference standard curves were printed along with the experimental samples for internal quality control. Selected arrays were stained with Sypro Ruby Protein Blot Stain (Molecular Probes, Eugene, OR, USA) following the manufacturer’s directions to quantify the total protein amount for each microarray spot and used for normalization purposes. Immunostaining was performed as previously described [38]. In brief, each array was probed with one polyclonal or monoclonal primary antibody targeting a protein of interest using an automated system (Dako Cytomation, Carpinteria, CA, USA). Primary antibodies were validated for specificity by western blotting on commercial cell lysates or human tissue lysates to test their specificity against the target protein [39]. Arrays were probed with a total of 187 antibodies, of which 137 targeted post-translational modifications (Table S4). Primary antibodies were recognized by a biotinylated anti-rabbit (Vector Laboratories, Inc.) or anti-mouse secondary antibody (Dako Cytomation, Carpinteria, CA, USA). Signal amplification was achieved using a commercially available tyramide-based avidin/biotin amplification system (Catalyzed Signal Amplification System (CSA); Dako Cytomation, Carpinteria, CA, USA) coupled with fluorescent detection using the streptavidin-conjugated IRDye680 dye (LI-COR Biosciences, Lincoln, NE, USA) according to the manufacturer’s instructions. Selected arrays were incubated with the secondary antibodies alone to capture background and unspecific signal. Images were acquired using a laser scanner (TECAN PowerScanner, Mönnedorf, Switzerland). Spot intensity was quantified using the commercially available software (MicroVigene V5.1.0.0; Vigenetech, Carlisle, MA, USA) [40]. In brief, samples were normalized to total protein values, the background and unspecific signal were subtracted, and technical replicates (*n* = 3) were averaged so that a single output value was generated for each sample. Coefficient of variations across technical replicates were <5%.

### 4.4. Drug Susceptibility Analysis

The effect of DMSO on drug sensitivity was assessed using archived data retrieved from the open-access Genomics of Drug Sensitivity in Cancer (GDSC) database (https://www.cancerrxgene.org, accessed on 1 February 2021). For the work, the GDSC2 dataset released in 2019 was used. As per database instructions, this version of the database drug sensitivity data was collected along with DMSO control data and used for normalization purposes. For each cell line, normalized IC50 are provided for a large panel of targeted compounds and chemotherapeutics. Cell lines were tested with an average of 180 drugs, with the exception of the H1838 and H1734 which were treated with 101 and 29 compounds, respectively. Normalized IC50 scores expressed as Z-scores were retrieved for each cell line. Per database instructions, cell lines with Z-scores > +2 were considered resistant to treatment while those with Z-scores < −2 were classified as sensitive.

### 4.5. Statistical Analysis

Kernel density plots and related histograms were created using R software version 3.4.3. In brief, for each treatment/time pair, replicate measurements were collected (*n* = 3) and protein networks were displayed using a graphical presentation where vertices represent individual proteins. 

Transformed correlations given by

(1) $$w_{u,v}=0.5\left(1+Corr\left(u,v\right)\right),$$

were used to establish edge weights, where $Corr\left(u,v\right)$ represents the Pearson correlation between the expression or activations of the proteins/vertices u and v. This approach forces the edge weights to take values between 0 and 1, with 1 indicating perfect correlation and 0 representing a negative correlation as previously described [41]. After constructing adjacency matrices for each cell/time pair, histograms and kernel density plots were built for the resulting (weighted) degree distributions, where degree was defined as

(2) $$\deg\left(u\right)=\underset{u\sim v}{\sum }w_{u,v};$$

(i.e., the degree of a vertex is the sum of its edge weights). Since the probability of having any protein pair with a perfectly negative correlation is extremely low, the topological structure for the untreated and DMSO networks are effectively the same. Thus, the different edge weights define the changes in protein expression and activation across experimental conditions. 

The two-tailed Kruskal Wallis rank test, a non-parametric one-way analysis of variance method, was used to identify changes in protein expression/activation levels across times and DMSO concentrations (C1: 0.0008%, C2: 0.002%, C3: 0.004%). Alpha level for significance was set at 0.05. In order to correct for inflated Type-I error from multiple group comparisons. For each cell line, RPPA values are shown using bar graphs created in GraphPad version v.6.07. RPPA values were first normalized, within each cell line, to baseline level; bar graphs visualize mean and standard error for each group. Venn Diagrams were created in a Gene List Venn Diagram. Pathway enrichment analysis was performed in Reactome Knowledgebase version 75 [17,18], and pathway visualization was conducted in String version 11.0b [42].

## Data Availability

Data will be made available upon reasonable request.

## Supplementary Materials

The following are available online at https://www.mdpi.com/1422-0067/22/6/2819/s1, Figure S1: Cell viability after 72 h of incubation with a wide range of DMSO doses, Figure S2: Histograms and kernel density plots representing weighted Pearson’s correlation coefficients for each DMSO dose across timepoints for six cell lines, Figure S3: Matrix capturing proteins that were statistically different across timepoints in eight lung cancer in vitro models, Figure S4: Enrichment analysis of selected pathways using the Reactome Knowledgebase software. Table S1: List of proteins with the corresponding *p* value from the non-parametric, two-tailed Kruskal Wallis rank test performed for each cell line and within each concentration, Table S2: Number of endpoints statistically different across cell lines for the three DMSO concentrations, Table S3: List of pathways enriched for each cell line based on the Reactome Knowledgebase software analysis, Table S4: List of antibodies used for the RPPA analysis including target proteins, vendors, catalogue numbers, and dilutions.
