# Supplementary material for: Heterogeneous Off-Target Effects of Ultra-Low Dose Dimethyl Sulfoxide (DMSO) on Targetable Signaling Events in Lung Cancer In Vitro Models
Source: Int J Mol Sci. 2021 Mar 10;22(6):2819. doi: 10.3390/ijms22062819 (PMC8001778; doi:10.3390/ijms22062819)
Supplement: Supplementary file 1 [file ijms-22-02819-s001.pdf]

# Supplementary Material

# Heterogeneous Off-Target Effects of Ultra-Low Dose Dimethyl Sulfoxide (DMSO) on Targetable Signaling Events in Lung Cancer *In Vitro* Models


**Elisa Baldelli [1], Mahalakshmi Subramanian [2,†], Abduljalil M. Alsubaie [2,†], Guy Oldaker [3], Maria Emelianenko [3], Emna El Gazzah [2], Sara Baglivo [4], Kimberley A. Hodge [1], Fortunato Bianconi [5], Vienna Ludovini [4], Lucio Crino' [6], Emanuel F. Petricoin [1], and Mariaelena Pierobon [1,2,*]**

[1] Center for Applied Proteomics and Molecular Medicine, George Mason University, Manassas, 20110 VA, USA; ebaldell@gmu.edu (E.B.); khodge5@gmu.edu (K.A.H.); epetrico@gmu.edu (E.F.P.)
[2] School of Systems Biology, George Mason University, Manassas, 20110 VA, USA; mahaaeiou93@gmail.com (M.S.); aalsuba5@masonlive.gmu.edu (A.M.A.); emnagazzah@yahoo.com (E.E)
[3] Department of Mathematical Science, George Mason University, Fairfax, 22030 VA, USA; goldaker@gmu.edu (G.O.); memelian@gmu.edu (M.E.)
[4] Division of Medical Oncology, S. Maria della Misericordia Hospital, 06156 Perugia, Italy; baglivosara@gmail.com (S.B.); oncolab@hotmail.com (V.L.)
[5] Independent Researcher, Belvedere 44, Montefalco, 06036, Perugia, Italy; fortunato.bianconi@gmail.com (F.B.);
[6] Department of Medical Oncology, Istituto Scientifico Romagnolo per lo Studio e la Cura dei Tumori (IRST) Istituto di ricovero e cura a carattere scientifico (IRCCS), Meldola 47014, Italy; lucio.crino@irst.emr.it (L.C.)
* Correspondence: mpierobo@gmu.edu
† These authors contributed equally to this work.


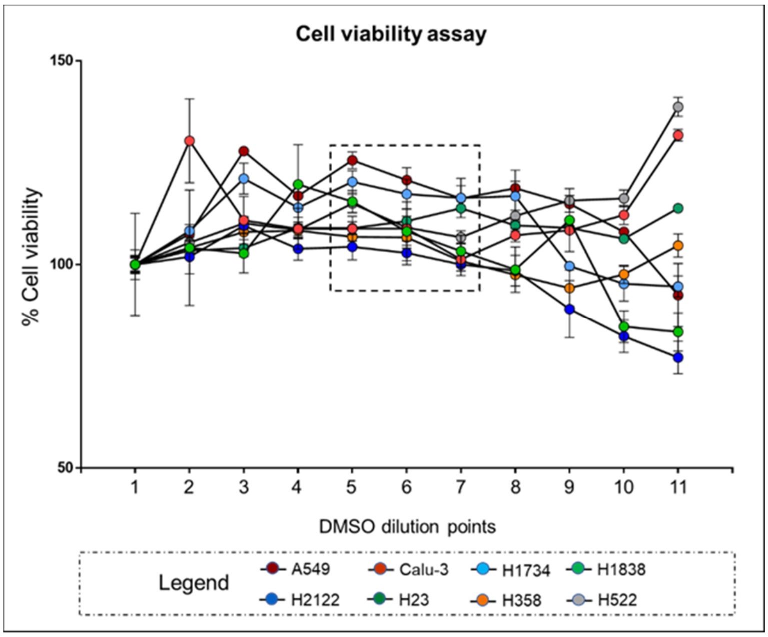


**Figure S1.** Cell viability after 72 hours of incubation with a wide range of DMSO doses. Cell lines were treated with 11 different DMSO concentrations for 72 hours in technical replicates (n=4) using a 2-fold serial dilution curve with a starting concentration of 0.05% v/v. Cell viability was assessed by a luminescence-based detection method and the effect of DMSO on cell growth was calculated as percentage change of RLU normalized to the lowest DMSO concentration. Results are presented as mean and standard error for the four technical replicates. The dashed square highlights the three DMSO concentrations selected for molecular analyses.

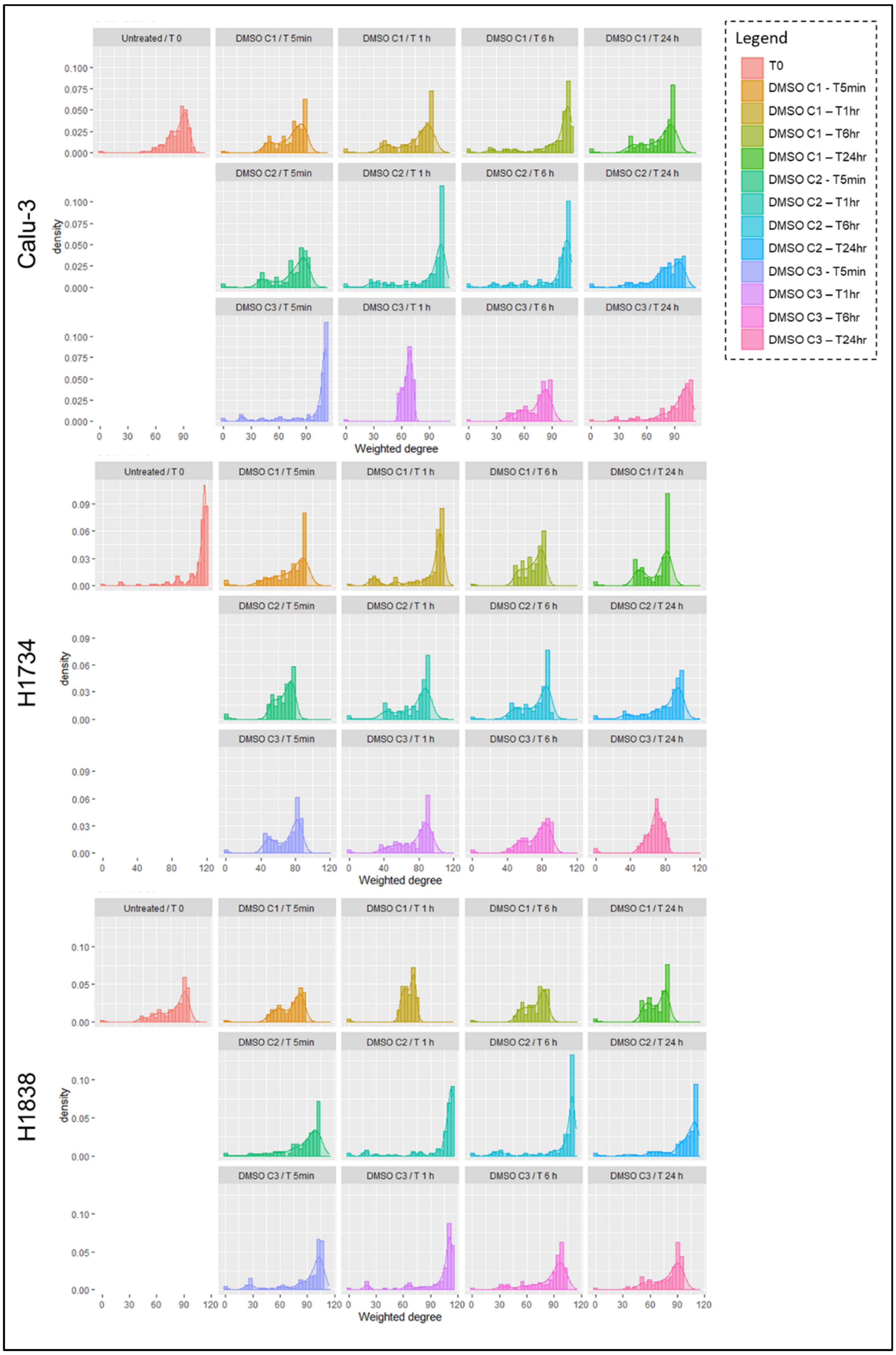

Calu-3
H1734
H1838
density
Weighted degree
Untreated / T 0
DMSO C1 / T 5min
DMSO C1 / T 1 h
DMSO C1 / T 6 h
DMSO C1 / T 24 h
DMSO C2 / T 5min
DMSO C2 / T 1 h
DMSO C2 / T 6 h
DMSO C2 / T 24 h
DMSO C3 / T 5min
DMSO C3 / T 1 h
DMSO C3 / T 6 h
DMSO C3 / T 24 h
Legend
T0
DMSO C1 - T5min
DMSO C1 – T1hr
DMSO C1 – T6hr
DMSO C1 – T24hr
DMSO C2 - T5min
DMSO C2 – T1hr
DMSO C2 – T6hr
DMSO C2 – T24hr
DMSO C3 - T5min
DMSO C3 – T1hr
DMSO C3 – T6hr
DMSO C3 – T24hr

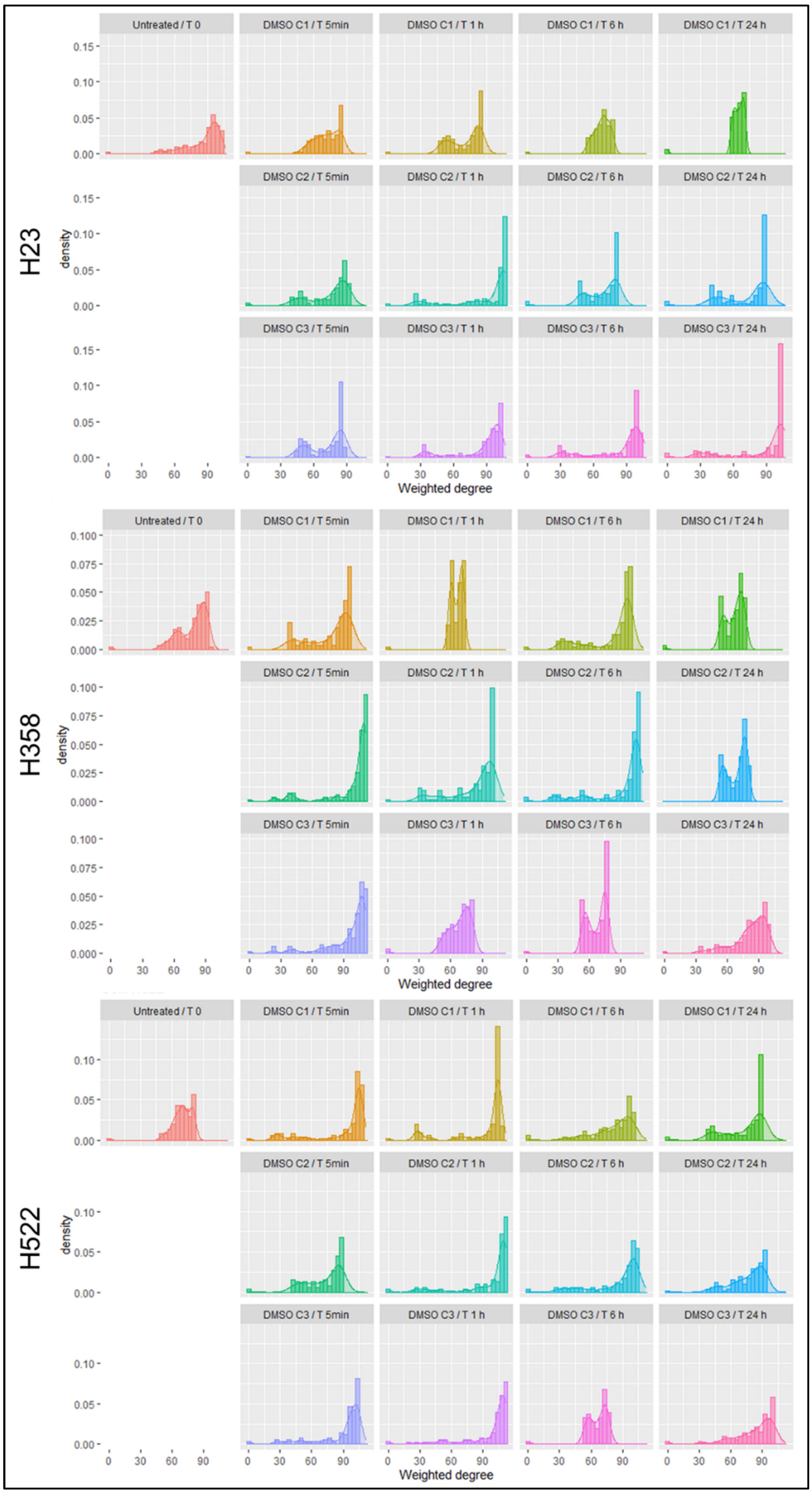


**Figure S2.** Histograms and kernel density plots representing weighted Pearson's correlation coefficients for each DMSO dose across timepoints for six cell lines. Weighted Pearson's correlation coefficients were calculated for paired proteins across all measured analytes and degree of distribution of pair-wise interconnections between analytes are shown for each cell lines, DMSO concentration, and timepoints.

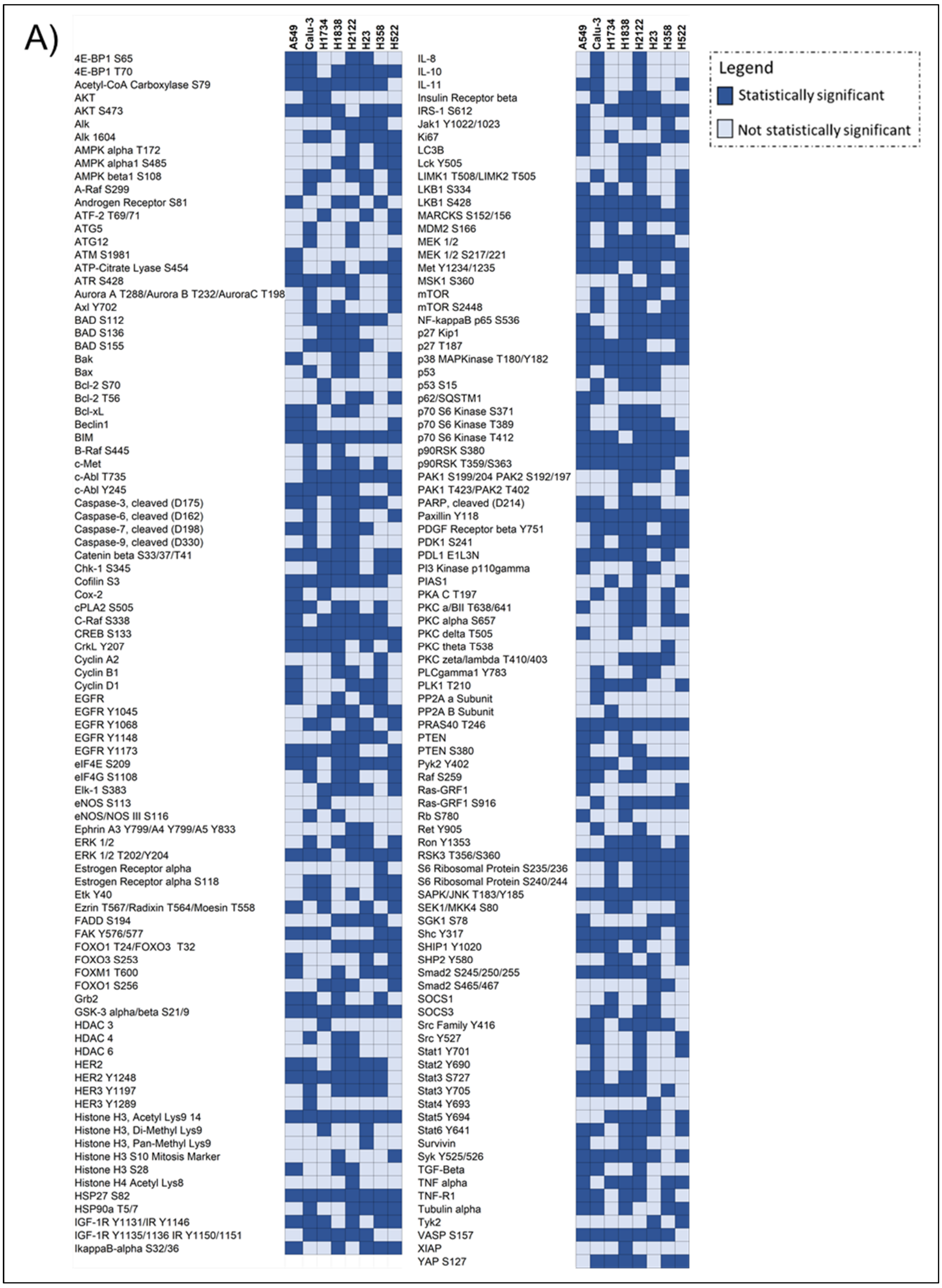
A)
A549
Calu-3
H1734
H1838
H2122
H23
H358
H522
4E-BP1 S65
4E-BP1 T70
Acetyl-CoA Carboxylase S79
AKT
AKT S473
Alk
Alk 1604
AMPK alpha T172
AMPK alpha1 S485
AMPK beta1 S108
A-Raf S299
Androgen Receptor S81
ATF-2 T69/71
ATG5
ATG12
ATM S1981
ATP-Citrate Lyase S454
ATR S428
Aurora A T288/Aurora B T232/AuroraC T198
Axl Y702
BAD S112
BAD S136
BAD S155
Bak
Bax
Bcl-2 S70
Bcl-2 T56
Bcl-xL
Beclin1
BIM
B-Raf S445
c-Met
c-Abl T735
c-Abl Y245
Caspase-3, cleaved (D175)
Caspase-6, cleaved (D162)
Caspase-7, cleaved (D198)
Caspase-9, cleaved (D330)
Catenin beta S33/37/T41
Chk-1 S345
Cofilin S3
Cox-2
cPLA2 S505
C-Raf S338
CREB S133
CrkL Y207
Cyclin A2
Cyclin B1
Cyclin D1
EGFR
EGFR Y1045
EGFR Y1068
EGFR Y1148
EGFR Y1173
eIF4E S209
eIF4G S1108
Elk-1 S383
eNOS S113
eNOS/NOS III S116
Ephrin A3 Y799/A4 Y799/A5 Y833
ERK 1/2
ERK 1/2 T202/Y204
Estrogen Receptor alpha
Estrogen Receptor alpha S118
Etk Y40
Ezrin T567/Radixin T564/Moesin T558
FADD S194
FAK Y576/577
FOXO1 T24/FOXO3 T32
FOXO3 S253
FOXM1 T600
FOXO1 S256
Grb2
GSK-3 alpha/beta S21/9
HDAC 3
HDAC 4
HDAC 6
HER2
HER2 Y1248
HER3 Y1197
HER3 Y1289
Histone H3, Acetyl Lys9 14
Histone H3, Di-Methyl Lys9
Histone H3, Pan-Methyl Lys9
Histone H3 S10 Mitosis Marker
Histone H3 S28
Histone H4 Acetyl Lys8
HSP27 S82
HSP90a T5/7
IGF-1R Y1131/IR Y1146
IGF-1R Y1135/1136 IR Y1150/1151
IkappaB-alpha S32/36
IL-8
IL-10
IL-11
Insulin Receptor beta
IRS-1 S612
Jak1 Y1022/1023
Ki67
LC3B
Lck Y505
LIMK1 T508/LIMK2 T505
LKB1 S334
LKB1 S428
MARCKS S152/156
MDM2 S166
MEK 1/2
MEK 1/2 S217/221
Met Y1234/1235
MSK1 S360
mTOR
mTOR S2448
NF-kappaB p65 S536
p27 Kip1
p27 T187
p38 MAPKinase T180/Y182
p53
p53 S15
p62/SQSTM1
p70 S6 Kinase S371
p70 S6 Kinase T389
p70 S6 Kinase T412
p90RSK S380
p90RSK T359/S363
PAK1 S199/204 PAK2 S192/197
PAK1 T423/PAK2 T402
PARP, cleaved (D214)
Paxillin Y118
PDGF Receptor beta Y751
PDK1 S241
PDL1 E1L3N
PI3 Kinase p110gamma
PIAS1
PKA C T197
PKC a/BII T638/641
PKC alpha S657
PKC delta T505
PKC theta T538
PKC zeta/lambda T410/403
PLCgamma1 Y783
PLK1 T210
PP2A a Subunit
PP2A B Subunit
PRAS40 T246
PTEN
PTEN S380
Pyk2 Y402
Raf S259
Ras-GRF1
Ras-GRF1 S916
Rb S780
Ret Y905
Ron Y1353
RSK3 T356/S360
S6 Ribosomal Protein S235/236
S6 Ribosomal Protein S240/244
SAPK/JNK T183/Y185
SEK1/MKK4 S80
SGK1 S78
Shc Y317
SHIP1 Y1020
SHP2 Y580
Smad2 S245/250/255
Smad2 S465/467
SOCS1
SOCS3
Src Family Y416
Src Y527
Stat1 Y701
Stat2 Y690
Stat3 S727
Stat3 Y705
Stat4 Y693
Stat5 Y694
Stat6 Y641
Survivin
Syk Y525/526
TGF-Beta
TNF alpha
TNF-R1
Tubulin alpha
Tyk2
VASP S157
XIAP
YAP S127
Legend
Statistically significant
Not statistically significant

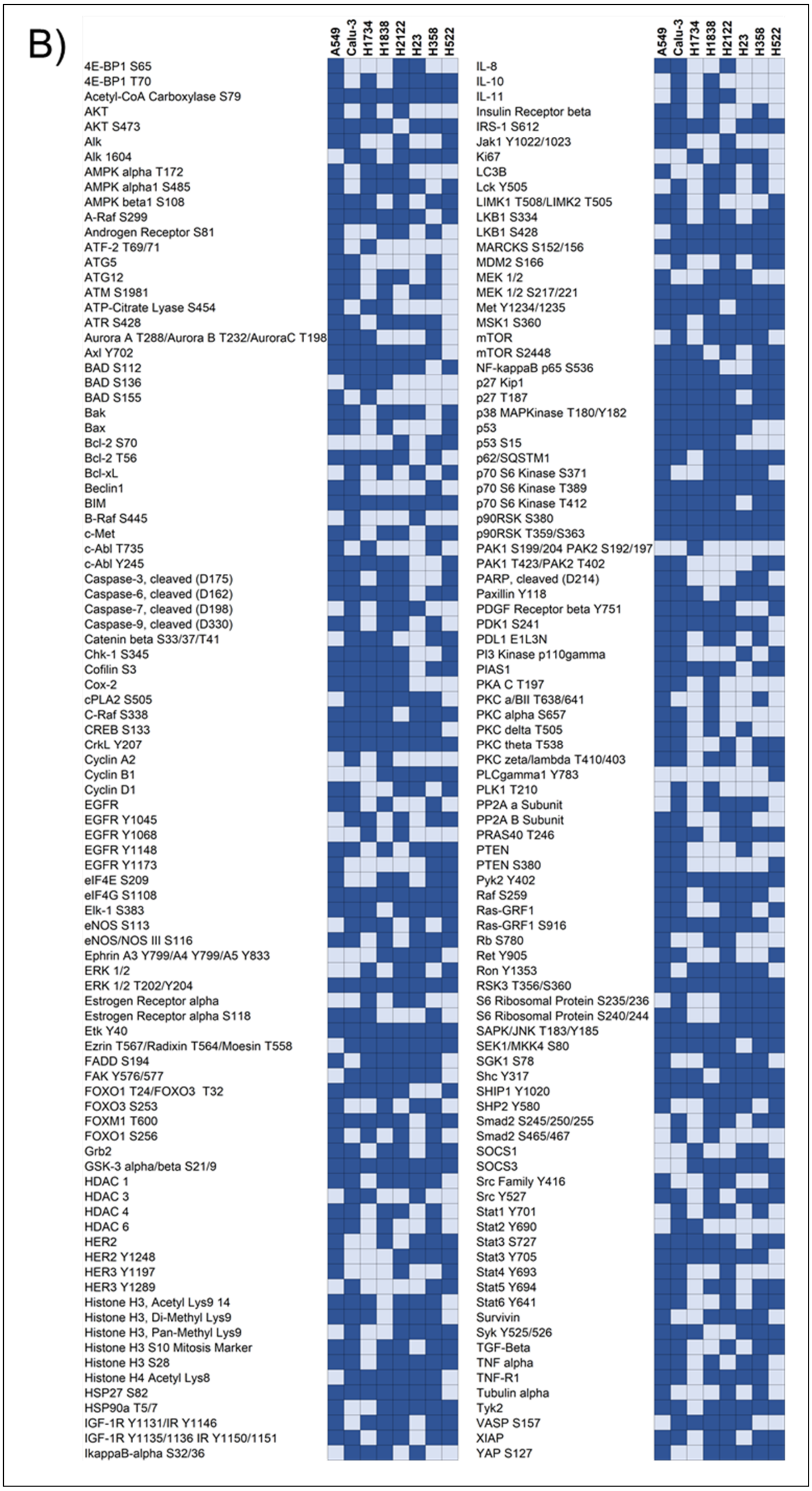
B)
A549
Calu-3
H1734
H1838
H2122
H23
H358
H522
4E-BP1 S65
4E-BP1 T70
Acetyl-CoA Carboxylase S79
AKT
AKT S473
Alk
Alk 1604
AMPK alpha T172
AMPK alpha1 S485
AMPK beta1 S108
A-Raf S299
Androgen Receptor S81
ATF-2 T69/71
ATG5
ATG12
ATM S1981
ATP-Citrate Lyase S454
ATR S428
Aurora A T288/Aurora B T232/AuroraC T198
Axl Y702
BAD S112
BAD S136
BAD S155
Bak
Bax
Bcl-2 S70
Bcl-2 T56
Bcl-xL
Beclin1
BIM
B-Raf S445
c-Met
c-Abl T735
c-Abl Y245
Caspase-3, cleaved (D175)
Caspase-6, cleaved (D162)
Caspase-7, cleaved (D198)
Caspase-9, cleaved (D330)
Catenin beta S33/37/T41
Chk-1 S345
Cofilin S3
Cox-2
cPLA2 S505
C-Raf S338
CREB S133
CrkL Y207
Cyclin A2
Cyclin B1
Cyclin D1
EGFR
EGFR Y1045
EGFR Y1068
EGFR Y1148
EGFR Y1173
eIF4E S209
eIF4G S1108
Elk-1 S383
eNOS S113
eNOS/NOS III S116
Ephrin A3 Y799/A4 Y799/A5 Y833
ERK 1/2
ERK 1/2 T202/Y204
Estrogen Receptor alpha
Estrogen Receptor alpha S118
Etk Y40
Ezrin T567/Radixin T564/Moesin T558
FADD S194
FAK Y576/577
FOXO1 T24/FOXO3 T32
FOXO3 S253
FOXM1 T600
FOXO1 S256
Grb2
GSK-3 alpha/beta S21/9
HDAC 1
HDAC 3
HDAC 4
HDAC 6
HER2
HER2 Y1248
HER3 Y1197
HER3 Y1289
Histone H3, Acetyl Lys9 14
Histone H3, Di-Methyl Lys9
Histone H3, Pan-Methyl Lys9
Histone H3 S10 Mitosis Marker
Histone H3 S28
Histone H4 Acetyl Lys8
HSP27 S82
HSP90a T5/7
IGF-1R Y1131/IR Y1146
IGF-1R Y1135/1136 IR Y1150/1151
IkappaB-alpha S32/36
IL-8
IL-10
IL-11
Insulin Receptor beta
IRS-1 S612
Jak1 Y1022/1023
Ki67
LC3B
Lck Y505
LIMK1 T508/LIMK2 T505
LKB1 S334
LKB1 S428
MARCKS S152/156
MDM2 S166
MEK 1/2
MEK 1/2 S217/221
Met Y1234/1235
MSK1 S360
mTOR
mTOR S2448
NF-kappaB p65 S536
p27 Kip1
p27 T187
p38 MAPKinase T180/Y182
p53
p53 S15
p62/SQSTM1
p70 S6 Kinase S371
p70 S6 Kinase T389
p70 S6 Kinase T412
p90RSK S380
p90RSK T359/S363
PAK1 S199/204 PAK2 S192/197
PAK1 T423/PAK2 T402
PARP, cleaved (D214)
Paxillin Y118
PDGF Receptor beta Y751
PDK1 S241
PDL1 E1L3N
PI3 Kinase p110gamma
PIAS1
PKA C T197
PKC a/BII T638/641
PKC alpha S657
PKC delta T505
PKC theta T538
PKC zeta/lambda T410/403
PLCgamma1 Y783
PLK1 T210
PP2A a Subunit
PP2A B Subunit
PRAS40 T246
PTEN
PTEN S380
Pyk2 Y402
Raf S259
Ras-GRF1
Ras-GRF1 S916
Rb S780
Ret Y905
Ron Y1353
RSK3 T356/S360
S6 Ribosomal Protein S235/236
S6 Ribosomal Protein S240/244
SAPK/JNK T183/Y185
SEK1/MKK4 S80
SGK1 S78
Shc Y317
SHIP1 Y1020
SHP2 Y580
Smad2 S245/250/255
Smad2 S465/467
SOCS1
SOCS3
Src Family Y416
Src Y527
Stat1 Y701
Stat2 Y690
Stat3 S727
Stat3 Y705
Stat4 Y693
Stat5 Y694
Stat6 Y641
Survivin
Syk Y525/526
TGF-Beta
TNF alpha
TNF-R1
Tubulin alpha
Tyk2
VASP S157
XIAP
YAP S127

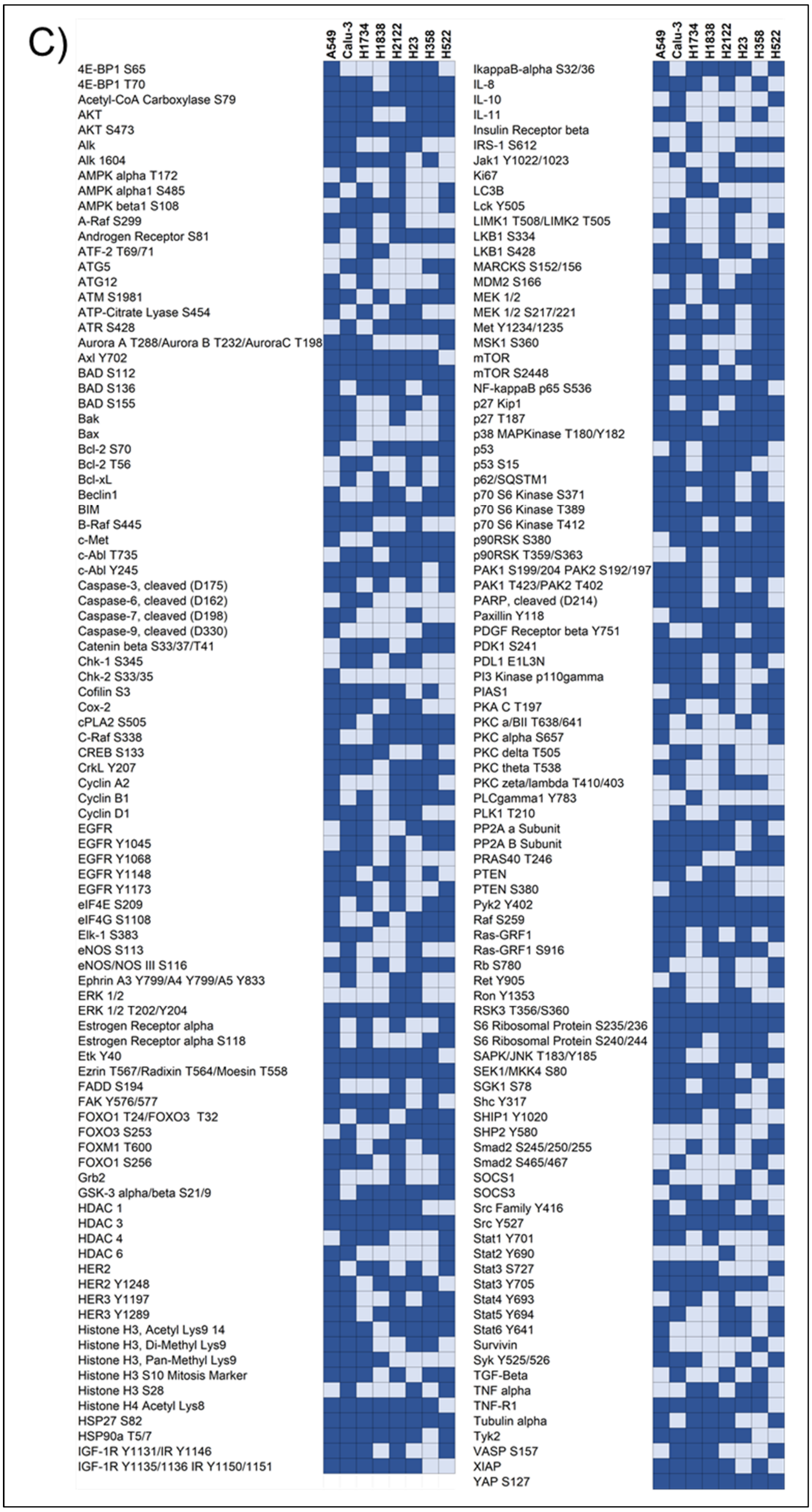

**Figure S3.** Matrix capturing proteins that were statistically different across timepoints in eight lung cancer *in vitro* models. For each analyte, changes in protein expression or activation across timepoints (n=5) were explored using the two-tailed Kruskal Wallis rank test. Proteins that reached statistical significance ($p<0.05$) across all three DMSO concentrations are shown for each cell line (C1 Panel **A**; C2 Panel **B** and C3 Panel **C** respectively).

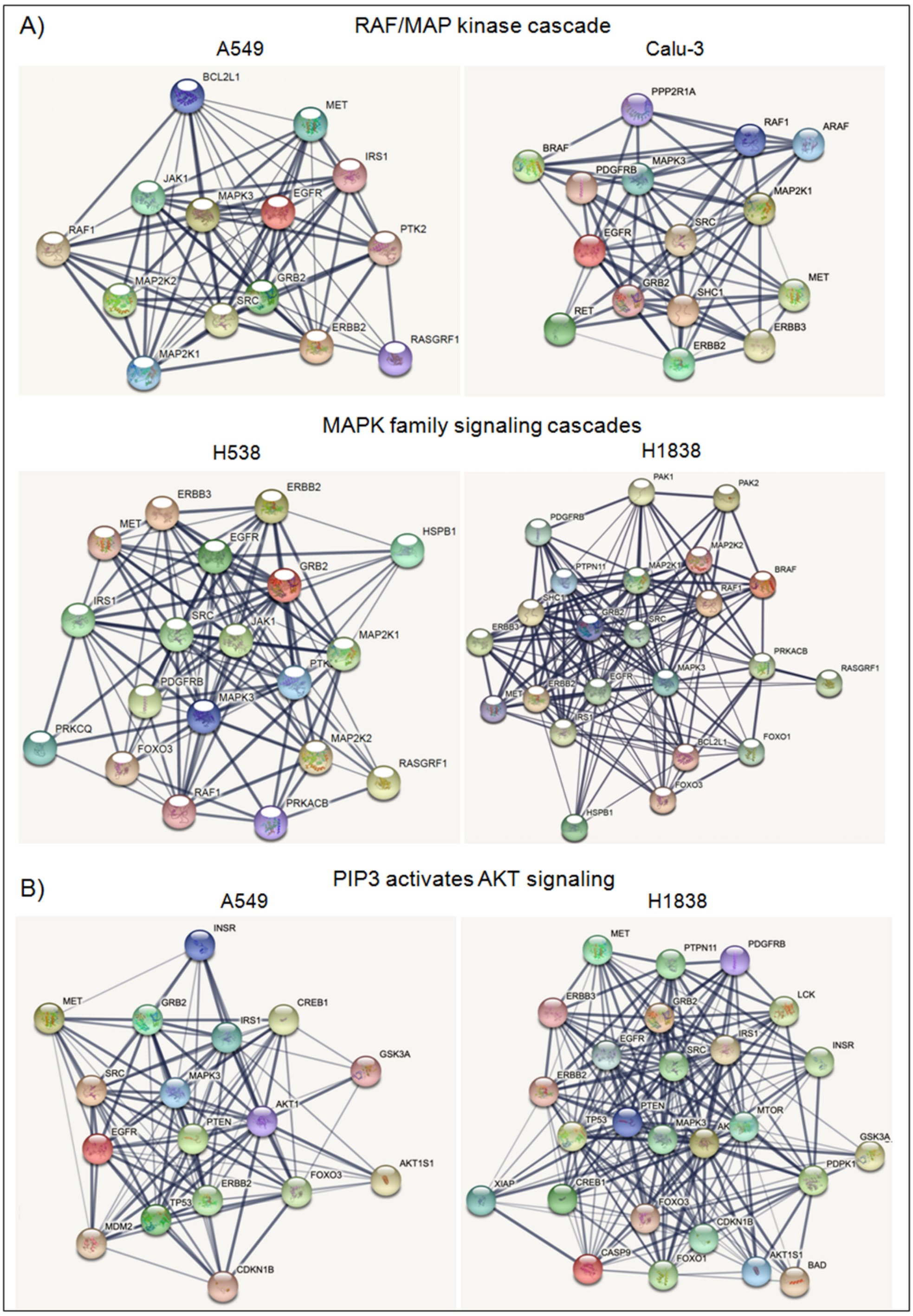


**Figure S4.** Enrichment analysis of selected pathways using the Reactome Knowledgebase software. Interconnected members are shown for: the RAF/MAP kinase cascade in A549 and Calu-3 cell lines (Panel **A**); the MAPK family signaling cascade in H538 and H1838 models (Panel **B**); the PIP3 activated AKT signaling in A549 and H1838 cell lines.

**Table S1.** List of proteins with the corresponding p value from the non-parametric, two-tailed Kruskal Wallis rank test performed for each cell line and within each concentration. In bold are represented comparisons that were statistical significance ($p< 0.05$).

| Proteins (C1) | A549 | Calu-3 | H1734 | H1838 | H2122 | H23 | H358 | H522 |
|---|---|---|---|---|---|---|---|---|
| 4E-BP1 S65 | **p <0.01** | **0.03** | 0.09 | 0.46 | **p <0.01** | **p <0.01** | 0.11 | 0.26 |
| 4E-BP1 T70 | **p <0.01** | **0.03** | 0.11 | **0.05** | **p <0.01** | **p <0.01** | **0.04** | **0.03** |
| Acetyl-CoA Carboxylase S79 | **p <0.01** | **p <0.01** | **0.02** | **0.01** | **p <0.01** | **p <0.01** | **p <0.01** | 0.09 |
| AKT | 0.41 | **0.02** | 0.05 | 0.10 | 0.16 | 0.07 | 0.22 | 0.13 |
| AKT S473 | **p <0.01** | **p <0.01** | **0.01** | **0.01** | 0.78 | **p <0.01** | **p <0.01** | **p <0.01** |
| Alk | 0.18 | 0.07 | 0.20 | **0.03** | **0.05** | **0.03** | **0.05** | 0.28 |
| Alk Y1604 | 0.16 | **p <0.01** | **0.01** | 0.06 | **p <0.01** | **0.04** | **0.02** | **p <0.01** |
| AMPK alpha T172 | 0.47 | 0.38 | 0.15 | 0.63 | **p <0.01** | 0.10 | **0.04** | **0.05** |
| AMPK alpha1 S485 | 0.74 | 0.07 | 0.06 | **0.01** | **0.04** | 0.06 | **0.03** | **0.04** |
| AMPK beta1 S108 | 0.25 | **p <0.01** | **0.02** | 0.06 | **p <0.01** | **0.02** | 0.52 | **0.03** |
| Androgen Receptor S81 | **p <0.01** | 0.08 | 0.38 | **0.02** | **0.05** | 0.07 | **p <0.01** | 0.40 |
| A-Raf S299 | 0.14 | **0.01** | 0.08 | 0.10 | 0.13 | **p <0.01** | 0.81 | **0.01** |
| ATF-2 T69/71 | 0.09 | 0.41 | **p <0.01** | 0.18 | 0.48 | **0.01** | 0.07 | **0.02** |
| ATG12 | 0.39 | **0.03** | 0.69 | 0.21 | **0.02** | 0.21 | 0.62 | 0.63 |
| ATG5 | 0.59 | **0.05** | 0.26 | 0.35 | **0.02** | 0.10 | 0.57 | **0.05** |
| ATM S1981 | **0.03** | 0.09 | 0.13 | 0.06 | 0.06 | 0.49 | 0.76 | **0.04** |
| ATP-Citrate Lyase S454 | **0.01** | 0.56 | 0.08 | **0.02** | 0.26 | **0.03** | **0.04** | **0.03** |
| ATR S428 | **0.03** | **0.04** | **0.04** | **0.04** | **p <0.01** | 0.12 | 0.15 | **0.02** |
| Aurora A T288/Aurora B T232/Aurora C T198 | 0.89 | **0.01** | 0.36 | 0.09 | **0.05** | 0.16 | 0.06 | **0.03** |
| Axl Y702 | 0.09 | **p <0.01** | 0.54 | **0.01** | 0.16 | 0.08 | 0.76 | **0.04** |
| BAD S112 | 0.22 | **0.01** | **0.01** | **0.01** | **0.01** | **0.03** | **0.04** | 0.16 |
| BAD S136 | 0.56 | 0.47 | **0.05** | **p <0.01** | **p <0.01** | 0.13 | 0.10 | 0.17 |
| BAD S155 | 0.40 | **p <0.01** | **0.01** | **0.03** | **0.03** | **p <0.01** | 0.84 | 0.07 |
| Bak | **0.03** | 0.16 | 0.13 | **p <0.01** | **p <0.01** | 0.55 | 0.06 | **p <0.01** |
| Bax | 0.17 | **0.02** | 0.20 | **0.03** | **0.02** | 0.22 | 0.11 | **0.03** |
| Bcl-2 S70 | 0.39 | 0.35 | **0.05** | 0.64 | 0.24 | 0.56 | 0.25 | 0.07 |
| Bcl-2 T56 | 0.12 | 0.53 | **0.02** | 0.15 | **0.05** | **0.04** | 0.08 | **p <0.01** |
| Bcl-xL | **0.04** | **0.01** | 0.43 | **0.01** | **0.01** | 0.40 | 0.16 | 0.08 |
| Beclin1 | **0.04** | **0.05** | 0.29 | 0.20 | 0.06 | 0.50 | 0.24 | **0.05** |
| BIM | **0.02** | **0.05** | **0.02** | **0.02** | **p <0.01** | **0.03** | **p <0.01** | **0.01** |
| B-Raf S445 | 0.06 | **0.04** | 0.14 | **0.03** | 0.06 | 0.18 | 0.17 | 0.15 |
| c-Abl T735 | 0.76 | **0.05** | **0.05** | **0.03** | **p <0.01** | **0.03** | **0.01** | **p <0.01** |
| c-Abl Y245 | **p <0.01** | **0.01** | **p <0.01** | **0.01** | **p <0.01** | 0.14 | 0.25 | **p <0.01** |
| Caspase-3, cleaved (D175) | **p <0.01** | **p <0.01** | 0.06 | **0.01** | **0.04** | **0.03** | **0.03** | 0.13 |
| Caspase-6, cleaved (D162) | 0.09 | **0.02** | 0.07 | **p <0.01** | **0.01** | 0.66 | 0.06 | **0.05** |
| Caspase-7, cleaved (D198) | **0.02** | **p <0.01** | 0.06 | **p <0.01** | **p <0.01** | **p <0.01** | 0.17 | 0.54 |
| Caspase-9, cleaved (D330) | 0.06 | **0.05** | 0.17 | **p <0.01** | **0.04** | 0.27 | 0.20 | 0.41 |
| Catenin beta S33/37/T41 | **0.02** | **0.03** | **0.02** | **0.02** | **0.03** | 0.09 | **p <0.01** | **0.02** |
| Chk-1 S345 | 0.17 | 0.15 | **0.05** | **0.01** | **0.03** | 0.07 | **0.03** | **p <0.01** |
| Chk-2 S33/35 | 0.94 | 1.00 | 1.00 | 1.00 | 0.57 | 1.00 | 1.00 | 1.00 |
| c-Met | 0.10 | **0.03** | 0.30 | **p <0.01** | **0.05** | 0.16 | **0.05** | 0.20 |
| Cofilin S3 | **p <0.01** | **p <0.01** | **0.01** | **p <0.01** | **0.02** | **p <0.01** | **0.02** | 0.14 |
| Cox-2 | **0.01** | 0.20 | **0.02** | 0.20 | 0.08 | 0.59 | 0.25 | 0.49 |
| cPLA2 S505 | **0.02** | **0.01** | 0.08 | **0.01** | **0.02** | 0.06 | **p <0.01** | 0.23 |
| C-Raf S338 | **p <0.01** | 0.32 | **0.02** | **0.04** | **0.03** | **p <0.01** | **0.03** | 0.33 |
| CREB S133 | **0.01** | **p <0.01** | **0.05** | **p <0.01** | **0.03** | **p <0.01** | **p <0.01** | **p <0.01** |
| CrkL Y207 | **0.03** | **p <0.01** | **p <0.01** | **0.01** | 0.06 | **p <0.01** | 0.15 | **0.02** |
| Cyclin A2 | 0.37 | 0.09 | 0.32 | **0.02** | 0.07 | 0.47 | **0.02** | 0.09 |
| Cyclin B1 | **0.02** | 0.21 | 0.18 | **0.02** | **0.03** | 0.07 | **0.02** | 0.08 |
| Cyclin D1 | **0.01** | 0.09 | 0.27 | 0.09 | **p <0.01** | **0.05** | **p <0.01** | 0.14 |
| EGFR | **p <0.01** | 0.12 | 0.06 | **0.01** | 0.10 | **0.03** | **p <0.01** | 0.14 |
| EGFR Y1045 | 0.23 | 0.22 | **p <0.01** | **0.03** | **p <0.01** | 0.08 | **p <0.01** | **p <0.01** |
| EGFR Y1068 | 0.39 | **0.02** | **p <0.01** | 0.08 | **p <0.01** | **0.02** | 0.09 | **0.02** |
| EGFR Y1148 | 0.15 | 0.22 | 0.12 | **0.03** | **0.04** | **0.01** | **0.03** | 0.78 |

| | | | | | | | | |
|---|---|---|---|---|---|---|---|---|
| EGFR Y1173 | **0.04** | **0.02** | **0.02** | **0.02** | **p <0.01** | 0.35 | 0.20 | **0.02** |
| eIF4E S209 | **p <0.01** | **p <0.01** | 0.06 | **0.02** | **p <0.01** | **0.03** | **p <0.01** | **0.02** |
| eIF4G S1108 | 0.28 | **0.02** | 0.20 | **0.03** | **0.03** | 0.06 | 0.37 | **0.04** |
| Elk-1 S383 | 0.25 | 0.09 | **0.02** | **0.04** | **0.01** | **0.03** | 0.69 | **p <0.01** |
| eNOS S113 | 0.10 | 0.38 | **0.03** | 0.43 | 0.58 | 0.13 | 0.49 | 0.88 |
| eNOS/NOS III S116 | 0.10 | **p <0.01** | 0.06 | **0.03** | 0.15 | 0.07 | 0.06 | 0.37 |
| Ephrin A3 Y799/A4 Y799/A5 Y833 | 0.08 | 0.19 | 0.35 | 0.14 | **p <0.01** | **0.03** | 0.10 | 0.10 |
| ERK 1/2 | 0.34 | **0.04** | 0.06 | **0.04** | **p<0.01** | **0.01** | 0.07 | **0.04** |
| ERK 1/2 T202/Y204 | **p <0.01** | **p <0.01** | **p <0.01** | 0.10 | **p <0.01** | **p <0.01** | **p <0.01** | **p <0.01** |
| Estrogen Receptor alpha | 0.12 | 0.47 | 0.14 | 0.36 | 0.08 | 0.06 | **0.05** | 0.18 |
| Estrogen Receptor alpha S118 | 0.21 | **0.04** | **0.05** | 0.72 | 0.09 | 0.68 | **0.05** | **0.02** |
| Etk Y40 | 0.11 | **0.03** | **0.02** | 0.08 | **p <0.01** | 0.11 | **0.05** | **p <0.01** |
| Ezrin T567/Radixin T564/Moesin T558 | **0.02** | 0.60 | **0.05** | 0.08 | 0.10 | **p <0.01** | 0.20 | **p <0.01** |
| FADD S194 | 0.16 | 0.58 | 0.07 | **0.02** | **0.01** | **0.04** | **0.01** | 0.15 |
| FAK Y576/577 | **0.02** | **p <0.01** | **0.02** | 0.38 | 0.24 | 0.18 | **0.05** | **0.02** |
| FOXM1 T600 | **0.04** | 0.07 | 0.69 | **0.02** | 0.10 | **0.04** | **p <0.01** | **0.05** |
| FOXO1 S256 | 0.06 | 0.10 | **0.02** | **p <0.01** | **p <0.01** | 0.34 | 0.65 | **0.02** |
| FOXO1 T24/FOXO3 T32 | 0.06 | 0.17 | 0.30 | **0.02** | **p <0.01** | **0.05** | **0.01** | **0.02** |
| FOXO3 S253 | **0.05** | 0.70 | 0.31 | 0.16 | 0.16 | **0.04** | 0.47 | **0.05** |
| Grb2 | **p <0.01** | **0.04** | 0.39 | **p <0.01** | 0.16 | 0.09 | **0.02** | 0.08 |
| GSK-3 alpha/beta S21/9 | **0.02** | **p <0.01** | **0.04** | **p <0.01** | **p <0.01** | **0.04** | **p <0.01** | **p <0.01** |
| HDAC 1 | 0.09 | 0.07 | 0.14 | 0.23 | 0.09 | 0.14 | 0.20 | 0.14 |
| HDAC 3 | 1.00 | 0.94 | **p <0.01** | 0.64 | 0.80 | 0.55 | 0.92 | 1.00 |
| HDAC 4 | 0.17 | **0.02** | 0.10 | **0.03** | **0.04** | 0.29 | 0.44 | 0.20 |
| HDAC 6 | 0.11 | 0.09 | 0.18 | **0.02** | **0.03** | 0.33 | 0.55 | 0.13 |
| HER2 | **0.03** | **0.04** | 0.10 | **0.02** | **0.03** | **p <0.01** | **p <0.01** | 0.09 |
| HER2 Y1248 | **0.02** | **p <0.01** | **p <0.01** | **0.01** | **0.04** | **0.03** | **0.03** | 0.06 |
| HER3 Y1197 | 0.06 | **0.04** | 0.48 | **p <0.01** | **0.01** | **0.02** | **0.03** | 0.21 |
| HER3 Y1289 | 0.40 | **p <0.01** | 0.27 | 0.06 | 0.09 | 0.08 | 0.09 | 0.14 |
| Histone H3 S10 Mitosis Marker | 0.23 | 0.09 | 0.15 | **0.04** | 0.09 | 0.16 | 0.11 | **0.03** |
| Histone H3 S28 | **0.03** | 0.07 | 0.11 | **p <0.01** | **p <0.01** | 0.08 | 0.21 | 0.10 |
| Histone H3, Acetyl Lys9 14 | **0.03** | **0.01** | **0.03** | **0.02** | **p <0.01** | **p <0.01** | **p <0.01** | **0.03** |
| Histone H3, Di-Methyl Lys9 | 0.11 | 0.24 | **0.03** | 0.33 | 0.26 | **p <0.01** | 0.68 | 0.80 |
| Histone H3, Pan-Methyl Lys9 | 0.16 | 0.14 | 0.14 | 0.17 | 0.07 | **0.05** | 0.80 | 0.13 |
| Histone H4 Acetyl Lys8 | 0.36 | 0.08 | 0.10 | 0.39 | **0.04** | 0.18 | 0.71 | 0.12 |
| HSP27 S82 | **p <0.01** | **p <0.01** | **0.02** | **p <0.01** | **p <0.01** | **p <0.01** | **p <0.01** | **0.02** |
| HSP90a T5/7 | 0.19 | **0.03** | 0.20 | 0.12 | **p <0.01** | **p <0.01** | **0.02** | **0.04** |
| IGF-1R Y1131/IR Y1146 | **0.03** | **0.02** | **0.01** | 0.29 | **p <0.01** | 0.26 | **0.05** | **p <0.01** |
| IGF-1R Y1135/1136 IR Y1150/1151 | 0.07 | **p <0.01** | **0.04** | **p <0.01** | **0.02** | **0.03** | 0.11 | 0.06 |
| IkappaB-alpha S32/36 | **0.04** | 0.47 | 0.21 | **0.05** | 0.12 | **0.02** | **p <0.01** | **0.01** |
| IL-10 | 0.06 | **0.01** | 0.20 | 0.07 | **0.02** | 0.20 | 0.11 | 0.20 |
| IL-11 | **0.04** | **0.05** | 0.27 | 0.32 | **p <0.01** | 0.49 | 0.16 | **0.03** |
| IL-8 | 0.20 | **0.02** | 0.85 | 0.11 | **p <0.01** | 0.15 | 0.29 | 0.24 |
| Insulin Receptor beta | 0.10 | **p <0.01** | 0.25 | **0.02** | **p <0.01** | **0.01** | 0.08 | 0.70 |
| IRS-1 S612 | **p <0.01** | 0.10 | **p <0.01** | **0.05** | **0.02** | **p <0.01** | **0.02** | **p <0.01** |
| Jak1 Y1022/1023 | **0.03** | 0.09 | 0.89 | 0.10 | **0.02** | 0.30 | **0.04** | 0.52 |
| Ki67 | **0.01** | 0.76 | **0.05** | 0.19 | 0.06 | 0.06 | **p <0.01** | **0.05** |
| LC3B | **0.04** | 0.12 | 0.33 | **0.01** | **0.03** | **0.02** | 0.09 | 0.71 |
| Lck Y505 | 0.38 | 0.06 | 0.26 | **0.05** | **p <0.01** | 0.32 | 0.06 | 0.13 |
| LIMK1 T508/LIMK2 T505 | 0.33 | **0.02** | 0.12 | **0.05** | **0.03** | 0.31 | 0.37 | **p <0.01** |
| LKB1 S334 | **0.02** | 0.26 | **0.03** | 0.08 | **0.04** | 0.91 | 0.13 | **0.03** |
| LKB1 S428 | **0.02** | **0.04** | **p <0.01** | **p <0.01** | **p <0.01** | **0.04** | 0.49 | **0.02** |
| MARCKS S152/156 | **0.03** | **p <0.01** | **0.02** | **p <0.01** | **0.05** | **p <0.01** | **p <0.01** | **0.01** |
| MDM2 S166 | **0.02** | 0.06 | **0.02** | 0.09 | **p <0.01** | 0.06 | 0.07 | **0.05** |
| MEK 1/2 | **0.03** | 0.09 | **0.04** | **0.03** | **0.02** | **p<0.01** | **0.03** | 0.13 |
| MEK 1/2 S217/221 | **p <0.01** | **p <0.01** | **0.02** | **p <0.01** | **p <0.01** | **0.01** | **0.02** | **p <0.01** |
| Met Y1234/1235 | **0.04** | **p <0.01** | **p <0.01** | 0.09 | **p <0.01** | **0.03** | 0.23 | **p <0.01** |
| MSK1 S360 | 0.51 | 0.07 | 0.08 | **0.03** | 0.17 | **p <0.01** | **0.03** | **0.04** |

| | | | | | | | | |
|---|---|---|---|---|---|---|---|---|
| mTOR | 0.59 | **0.01** | 0.24 | **0.01** | **p<0.01** | **0.02** | 0.66 | **p<0.01** |
| mTOR S2448 | 0.12 | **0.02** | 0.06 | **0.04** | **p <0.01** | 0.12 | **0.05** | **0.01** |
| NF-kappaB p65 S536 | **0.01** | 0.10 | 0.08 | **0.02** | **0.03** | **0.03** | **0.02** | **p <0.01** |
| p27 Kip1 | **0.04** | **0.04** | 0.55 | **0.01** | **p <0.01** | **0.02** | **0.04** | **0.01** |
| p27 T187 | **p <0.01** | **p <0.01** | **0.01** | **0.02** | **0.02** | 0.14 | 0.30 | **0.05** |
| p38 MAP Kinase T180/Y182 | **p <0.01** | **p <0.01** | **p <0.01** | **p <0.01** | **p <0.01** | **p <0.01** | **0.03** | **0.02** |
| p53 | **0.01** | 0.14 | **0.04** | **p <0.01** | **0.04** | **p <0.01** | 0.09 | 0.09 |
| p53 S15 | 0.41 | **p <0.01** | 0.39 | **0.02** | **0.03** | **0.01** | 0.06 | 0.13 |
| p62/SQSTM1 | **0.05** | **0.03** | 0.53 | 0.09 | 0.08 | 0.08 | 0.38 | 0.25 |
| p70 S6 Kinase S371 | **p <0.01** | 0.40 | 0.11 | **0.02** | **p <0.01** | **0.02** | 0.07 | 0.12 |
| p70 S6 Kinase T389 | **0.01** | 0.09 | 0.17 | **0.01** | **p <0.01** | **0.02** | **0.01** | 0.06 |
| p70 S6 Kinase T412 | **0.02** | **p <0.01** | **0.02** | 0.13 | **p <0.01** | **0.03** | **0.02** | **p <0.01** |
| p90RSK S380 | **0.02** | **0.03** | **p <0.01** | **0.01** | **p <0.01** | **p <0.01** | **p <0.01** | **0.02** |
| p90RSK T359/S363 | **0.03** | **p <0.01** | **0.02** | **0.02** | **p <0.01** | **p <0.01** | **0.03** | 0.06 |
| PAK1 S199/204 PAK2 S192/197 | 0.06 | 0.07 | 0.13 | **0.03** | **0.05** | **0.02** | 0.21 | **0.05** |
| PAK1 T423/PAK2 T402 | 0.37 | 0.13 | 0.56 | **0.02** | 0.24 | 0.42 | 0.98 | **0.01** |
| PARP, cleaved (D214) | **0.01** | **0.01** | 0.06 | **p <0.01** | **0.03** | **0.02** | 0.42 | 0.06 |
| Paxillin Y118 | **0.02** | **0.01** | **p <0.01** | **0.05** | **p <0.01** | **p <0.01** | **p <0.01** | **p <0.01** |
| PDGF Receptor beta Y751 | 0.19 | **p <0.01** | **0.02** | **0.05** | **p <0.01** | 0.32 | **0.02** | **p <0.01** |
| PDK1 S241 | 0.14 | **p <0.01** | 0.14 | **0.01** | **p <0.01** | **p <0.01** | **p <0.01** | **0.02** |
| PDL1 E1L3N | **p <0.01** | **0.02** | **0.03** | **0.01** | **0.01** | 0.75 | **0.01** | 0.41 |
| PI3 Kinase p110gamma | **0.04** | 0.08 | 0.76 | 0.14 | **0.05** | **0.02** | 0.30 | 0.44 |
| PIAS1 | 0.35 | 0.10 | **0.03** | 0.12 | **0.01** | 0.21 | 0.10 | **0.01** |
| PKA C T197 | 0.09 | **0.03** | 0.35 | **0.03** | **p <0.01** | 0.71 | **0.04** | 0.24 |
| PKC a/BII T638/641 | **0.01** | 0.06 | 0.31 | **0.03** | **p <0.01** | 0.11 | **0.01** | 0.72 |
| PKC alpha S657 | 0.09 | 0.17 | 0.32 | **0.05** | **0.02** | 0.12 | **0.04** | **0.02** |
| PKC delta T505 | **0.05** | 0.28 | 0.32 | **0.03** | 0.23 | 0.33 | 0.23 | 0.19 |
| PKC theta T538 | 0.19 | 0.66 | 0.85 | 0.31 | 0.30 | 0.35 | **0.02** | 0.08 |
| PKC zeta/lambda T410/403 | 0.11 | 0.28 | 0.46 | **0.03** | **0.04** | **p <0.01** | **0.01** | 0.16 |
| PLCgamma1 Y783 | 0.07 | **0.03** | 0.17 | 0.66 | **0.02** | **0.02** | 0.17 | 0.75 |
| PLK1 T210 | 0.13 | **0.03** | **0.03** | **0.05** | **0.02** | 0.08 | 0.15 | **0.05** |
| PP2A a Subunit | 0.33 | **0.02** | 0.14 | 0.07 | 0.12 | 0.11 | 0.50 | 0.19 |
| PP2A B Subunit | 0.36 | 0.08 | **0.03** | 0.49 | 0.06 | 0.12 | 0.46 | 0.55 |
| PRAS40 T246 | **p <0.01** | **p <0.01** | **0.01** | **0.02** | **0.02** | **p <0.01** | **p <0.01** | **p <0.01** |
| PTEN | **0.03** | **0.04** | 0.37 | **0.02** | 0.09 | 0.10 | 0.10 | 0.57 |
| PTEN S380 | **0.03** | 0.06 | 0.17 | **0.02** | **0.01** | 0.71 | 0.13 | 0.07 |
| Pyk2 Y402 | **0.02** | **0.03** | **p <0.01** | 0.19 | **0.02** | **0.04** | **0.03** | **p <0.01** |
| Raf S259 | **0.02** | **0.04** | 0.31 | **0.01** | **0.01** | 0.19 | 0.06 | 0.06 |
| Ras-GRF1 | **0.04** | 0.10 | 0.45 | 0.06 | 0.09 | 0.13 | 0.12 | **0.02** |
| Ras-GRF1 S916 | 0.16 | **0.03** | 0.84 | **p <0.01** | **0.05** | **0.01** | **0.04** | **0.04** |
| Rb S780 | **0.01** | 0.10 | 0.57 | **0.02** | 0.06 | 0.08 | 0.09 | 0.19 |
| Ret Y905 | 0.29 | **0.04** | 0.37 | 0.06 | **0.03** | 0.17 | 0.12 | 0.06 |
| Ron Y1353 | 0.06 | 0.07 | **0.02** | **0.04** | **0.02** | **p <0.01** | 0.14 | **0.05** |
| RSK3 T356/S360 | **p <0.01** | **p <0.01** | **0.02** | **p <0.01** | **p <0.01** | **p <0.01** | **p <0.01** | **0.02** |
| S6 Ribosomal Protein S235/236 | 0.13 | 0.11 | **0.03** | 0.08 | **p <0.01** | **p <0.01** | **0.05** | **0.03** |
| S6 Ribosomal Protein S240/244 | 0.08 | 0.09 | **0.03** | 0.09 | **p <0.01** | **p <0.01** | **0.04** | **0.05** |
| SAPK/JNK T183/Y185 | **0.01** | **p <0.01** | **0.02** | **p <0.01** | **p <0.01** | **0.03** | **p <0.01** | **0.01** |
| SEK1/MKK4 S80 | 0.18 | 0.34 | **0.02** | **0.03** | 0.40 | 0.31 | 0.06 | **p <0.01** |
| SGK1 S78 | **0.05** | 0.25 | 0.19 | 0.21 | 0.10 | **0.03** | **0.04** | **0.05** |
| Shc Y317 | **0.04** | **p <0.01** | **p <0.01** | **0.02** | **p <0.01** | **0.04** | 0.07 | **0.03** |
| SHIP1 Y1020 | **p <0.01** | **0.03** | **0.04** | 0.09 | **0.01** | 0.12 | 0.07 | **p <0.01** |
| SHP2 Y580 | 0.93 | 0.42 | **0.04** | **p <0.01** | 0.07 | **p <0.01** | 0.40 | **0.02** |
| Smad2 S245/250/255 | **p <0.01** | **0.01** | **0.04** | **0.02** | **p <0.01** | **0.02** | 0.09 | 0.40 |
| Smad2 S465/467 | 1.00 | 0.11 | 0.93 | 0.19 | 1.00 | **0.01** | **0.02** | 0.93 |
| SOCS1 | 0.13 | 0.06 | **0.03** | 0.41 | 0.06 | **0.05** | 0.30 | 0.14 |
| SOCS3 | 0.12 | **0.03** | **0.04** | 0.07 | **0.03** | **0.03** | 0.32 | 0.07 |
| Src Family Y416 | **0.05** | **0.02** | 0.07 | **0.03** | **p <0.01** | **0.05** | **p <0.01** | 0.09 |
| Src Y527 | **0.01** | **0.01** | 0.14 | 0.07 | **0.01** | 0.08 | 0.37 | **p <0.01** |
| Stat1 Y701 | 0.93 | **p <0.01** | 0.32 | 0.06 | **0.01** | 0.14 | 0.13 | **0.05** |

| | | | | | | | | |
|---|---|---|---|---|---|---|---|---|
| Stat2 Y690 | 0.06 | **0.05** | 0.32 | 0.20 | **0.02** | 0.20 | 0.09 | 0.47 |
| Stat3 S727 | **0.02** | **0.04** | 0.06 | **0.03** | **p <0.01** | 0.07 | 0.22 | 0.50 |
| Stat3 Y705 | **p <0.01** | **0.03** | **0.01** | **p <0.01** | **p <0.01** | 0.10 | **0.01** | 0.32 |
| Stat4 Y693 | 0.83 | 0.19 | 0.97 | 0.09 | 0.12 | **0.03** | 0.07 | 0.09 |
| Stat5 Y694 | 0.10 | 0.28 | **0.01** | **0.02** | **p <0.01** | **0.02** | 0.08 | **p <0.01** |
| Stat6 Y641 | **0.05** | **p <0.01** | 0.19 | **0.03** | **p <0.01** | **0.03** | 0.13 | **p <0.01** |
| Survivin | **p <0.01** | 0.47 | 0.08 | **0.02** | **p <0.01** | 0.52 | 0.09 | 0.07 |
| Syk Y525/526 | **0.04** | **p <0.01** | **p <0.01** | **p <0.01** | **p <0.01** | **p <0.01** | 0.13 | **p <0.01** |
| TGF-Beta | **0.04** | **0.04** | 0.28 | 0.08 | **0.03** | 0.07 | 0.20 | 0.12 |
| TNF alpha | **0.01** | 0.11 | **0.04** | **0.01** | **0.03** | 0.07 | **p <0.01** | **0.03** |
| TNF-R1 | **p <0.01** | **0.03** | 0.19 | **0.01** | **p <0.01** | 0.06 | **0.01** | 0.15 |
| Tubulin alpha | **0.02** | **0.04** | 0.50 | **p <0.01** | 0.48 | 0.22 | **0.04** | **0.02** |
| Tyk2 | 0.13 | 0.09 | 0.57 | 0.10 | 0.28 | **0.03** | 0.95 | **0.01** |
| VASP S157 | **p <0.01** | **p <0.01** | **p <0.01** | **0.02** | **p <0.01** | **p <0.01** | **p <0.01** | 0.24 |
| XIAP | 0.14 | 0.11 | 0.71 | **0.02** | 0.07 | 0.33 | 0.09 | 0.19 |
| YAP S127 | 0.14 | **p <0.01** | **0.02** | **p <0.01** | **p <0.01** | 0.53 | **p <0.01** | **p <0.01** |
| **Proteins (C2)** | **A549** | **Calu-3** | **H1734** | **H1838** | **H2122** | **H23** | **H358** | **H522** |
| 4E-BP1 S65 | **p <0.01** | 0.09 | 0.12 | 0.06 | **p <0.01** | **0.04** | 0.07 | 0.09 |
| 4E-BP1 T70 | **p <0.01** | 0.08 | **0.02** | 0.08 | **p <0.01** | **p <0.01** | **0.05** | **p <0.01** |
| Acetyl-CoA Carboxylase S79 | **p <0.01** | **p <0.01** | **0.01** | **p <0.01** | **p <0.01** | **0.02** | **p <0.01** | **0.03** |
| AKT | **0.03** | 0.07 | **p<0.015** | 0.07 | **0.02** | 0.05 | 0.13 | 0.08 |
| AKT S473 | **p <0.01** | **p <0.01** | **p <0.01** | **0.02** | 0.07 | **p <0.01** | **p <0.01** | **p <0.01** |
| Alk | **0.01** | **0.04** | 0.27 | 0.12 | **0.01** | 0.12 | 0.06 | **0.02** |
| Alk Y1604 | 0.12 | **p <0.01** | **p <0.01** | 0.51 | **p <0.01** | **0.02** | **0.01** | **0.04** |
| AMPK alpha T172 | **p <0.01** | 0.16 | **0.01** | **0.03** | **p <0.01** | 0.65 | 0.20 | 0.29 |
| AMPK alpha1 S485 | **p <0.01** | 0.06 | **p <0.01** | **0.05** | **0.01** | **0.02** | 0.06 | **0.03** |
| AMPK beta1 S108 | **p <0.01** | **p <0.01** | **p <0.01** | 0.07 | **0.04** | 0.52 | **0.04** | **0.05** |
| Androgen Receptor S81 | **p <0.01** | 0.54 | 0.15 | **0.03** | **p <0.01** | 0.07 | **p <0.01** | 0.11 |
| A-Raf S299 | **0.02** | **p <0.01** | **0.05** | **0.05** | **p <0.01** | **0.03** | 0.19 | **0.03** |
| ATF-2 T69/71 | **0.02** | 0.34 | **p <0.01** | 0.46 | 0.57 | 0.19 | 0.30 | 0.17 |
| ATG12 | **0.02** | **p <0.01** | 0.30 | **0.02** | **0.03** | 0.25 | **0.05** | 0.19 |
| ATG5 | **0.05** | **0.01** | 0.13 | 0.07 | 0.51 | 0.15 | **0.05** | 0.11 |
| ATM S1981 | **0.02** | **0.02** | 0.63 | **p <0.01** | 0.79 | **0.05** | **0.01** | 0.09 |
| ATP-Citrate Lyase S454 | **0.03** | 0.16 | **0.01** | **p <0.01** | 0.28 | 0.15 | 0.07 | 0.11 |
| ATR S428 | **0.04** | **0.02** | 0.09 | **0.02** | **0.02** | **0.02** | **p <0.01** | 0.07 |
| Aurora A T288/Aurora B T232/Auro-raC T198 | **0.02** | **0.03** | **0.02** | 0.07 | 0.06 | 0.40 | **0.01** | 0.11 |
| Axl Y702 | **p <0.01** | **p <0.01** | **0.05** | **0.02** | **p <0.01** | **0.01** | **0.02** | 0.11 |
| BAD S112 | **p <0.01** | **0.03** | **p <0.01** | **p <0.01** | **p <0.01** | **0.01** | 0.19 | **0.01** |
| BAD S136 | 0.32 | **0.03** | **0.02** | **p <0.01** | 0.07 | 0.13 | 0.13 | 0.74 |
| BAD S155 | **0.02** | 0.07 | **0.01** | 0.09 | 0.44 | 0.62 | 0.24 | 0.17 |
| Bak | **0.01** | **0.01** | 0.26 | **0.02** | **0.01** | **p <0.01** | 0.36 | **0.02** |
| Bax | **p <0.01** | **p <0.01** | 0.07 | **0.02** | 0.33 | 0.09 | 0.12 | **0.02** |
| Bcl-2 S70 | 0.06 | 0.07 | 0.07 | 0.09 | **p <0.01** | 0.12 | **0.04** | **0.03** |
| Bcl-2 T56 | **0.05** | **0.01** | **p <0.01** | **0.03** | **0.02** | 0.16 | **0.03** | 0.16 |
| Bcl-xL | 0.18 | **p <0.01** | 0.90 | **0.02** | 0.09 | **0.04** | 0.67 | **0.02** |
| Beclin1 | **p <0.01** | **0.04** | 0.12 | 0.24 | 0.26 | 0.53 | **0.02** | 0.08 |
| BIM | **p <0.01** | **0.02** | **0.02** | **0.01** | **p <0.01** | **0.02** | **0.03** | **0.01** |
| B-Raf S445 | 0.11 | **0.02** | 0.08 | 0.06 | 0.15 | **0.05** | 0.10 | 0.23 |
| c-Abl T735 | **p <0.01** | 0.08 | **0.02** | 0.12 | 0.09 | 0.09 | **0.04** | 0.14 |
| c-Abl Y245 | **p <0.01** | **0.02** | **p <0.01** | **0.01** | **0.01** | 0.35 | 0.12 | **0.03** |
| Caspase-3, cleaved (D175) | **0.04** | **p <0.01** | 0.08 | **0.04** | **0.04** | 0.36 | 0.13 | **0.02** |
| Caspase-6, cleaved (D162) | **0.01** | **0.02** | **0.03** | **0.04** | **0.03** | 0.35 | **0.02** | **0.03** |
| Caspase-7, cleaved (D198) | 0.07 | **0.02** | 0.20 | **0.04** | **p <0.01** | **0.02** | 0.63 | 0.79 |
| Caspase-9, cleaved (D330) | **0.02** | **0.03** | 0.55 | **0.01** | **0.02** | 0.64 | **0.04** | 0.12 |
| Catenin beta S33/37/T41 | 0.10 | **0.05** | **0.02** | **0.01** | 0.10 | 0.20 | **0.05** | **0.05** |
| Chk-1 S345 | **p <0.01** | **0.03** | **0.03** | **0.02** | **0.03** | 0.06 | 0.23 | **0.03** |
| Chk-2 S33/35 | 0.12 | 1.00 | 0.53 | 1.00 | 0.94 | 1.00 | 0.94 | 0.94 |
| c-Met | **0.01** | **0.03** | 0.23 | **p <0.01** | **p <0.01** | 0.09 | **0.02** | **0.01** |

| | | | | | | | | |
|---|---|---|---|---|---|---|---|---|
| Cofilin S3 | **p <0.01** | **p <0.01** | **p <0.01** | **p <0.01** | **0.03** | 0.06 | **0.01** | **0.04** |
| Cox-2 | **0.01** | **0.03** | **0.02** | **0.03** | **0.01** | 0.06 | 0.19 | 0.87 |
| cPLA2 S505 | 0.06 | **p <0.01** | **0.05** | **0.01** | **p <0.01** | **0.01** | **p <0.01** | 0.06 |
| C-Raf S338 | **p <0.01** | **0.02** | **0.01** | **0.01** | 0.11 | **p <0.01** | **0.01** | **0.04** |
| CREB S133 | **0.03** | **0.02** | **0.01** | **p <0.01** | **0.01** | **p <0.01** | **p <0.01** | 0.12 |
| CrkL Y207 | **p <0.01** | **p <0.01** | **p <0.01** | **0.02** | **p <0.01** | **p <0.01** | **p <0.01** | **p <0.01** |
| Cyclin A2 | 0.31 | **0.03** | 0.82 | **0.02** | 0.06 | 0.35 | 0.38 | 0.33 |
| Cyclin B1 | 0.08 | 0.16 | 0.62 | **0.04** | **0.02** | **p <0.01** | **0.04** | **0.04** |
| Cyclin D1 | **p <0.01** | **0.05** | 0.13 | 0.09 | **p <0.01** | **0.02** | 0.14 | **0.05** |
| EGFR | **0.03** | **0.01** | 0.07 | **0.02** | 0.12 | 0.24 | **0.01** | 0.51 |
| EGFR Y1045 | 0.07 | **p <0.01** | **p <0.01** | 0.25 | **p <0.01** | 0.50 | **p <0.01** | **p <0.01** |
| EGFR Y1068 | 0.09 | 0.34 | **0.04** | 0.39 | **p <0.01** | 0.08 | 0.06 | 0.09 |
| EGFR Y1148 | **0.02** | **0.01** | 0.09 | **0.04** | 0.08 | **0.02** | **0.02** | **p <0.01** |
| EGFR Y1173 | **0.01** | 0.19 | 0.73 | 0.23 | 0.27 | 0.11 | **0.03** | **0.02** |
| eIF4E S209 | **p <0.01** | 0.50 | 0.07 | **p <0.01** | **p <0.01** | 0.19 | **0.04** | **p <0.01** |
| eIF4G S1108 | **p <0.01** | **0.03** | **0.03** | **0.02** | **0.05** | **0.01** | **p <0.01** | **0.01** |
| Elk-1 S383 | **0.03** | **0.01** | **0.01** | 0.06 | **p <0.01** | **0.02** | **0.05** | **p <0.01** |
| eNOS S113 | 0.19 | **0.04** | **0.02** | **0.03** | 0.09 | **0.02** | 0.09 | **0.05** |
| eNOS/NOS III S116 | **0.01** | **0.01** | 0.08 | **0.04** | 0.26 | **0.03** | **0.02** | **0.02** |
| Ephrin A3 Y799/A4 Y799/A5 Y833 | 0.13 | 0.15 | 0.68 | **0.05** | **p <0.01** | **0.04** | **0.01** | 0.33 |
| ERK 1/2 | 0.23 | 0.13 | **0.02** | 0.09 | **0.04** | **0.02** | 0.13 | **0.02** |
| ERK 1/2 T202/Y204 | **p <0.01** | **p <0.01** | **p <0.01** | **0.02** | **p <0.01** | **p <0.01** | **p <0.01** | **p <0.01** |
| Estrogen Receptor alpha | 0.40 | 0.07 | **0.04** | 0.22 | **0.02** | **0.03** | 0.37 | 0.30 |
| Estrogen Receptor alpha S118 | **p <0.01** | **0.04** | **0.02** | 0.08 | 0.06 | 0.84 | **0.01** | 0.17 |
| Etk Y40 | **p <0.01** | **0.05** | **0.02** | **0.02** | **p <0.01** | **0.02** | **0.02** | **0.03** |
| Ezrin T567/Radixin T564/Moesin T558 | 0.19 | **0.05** | **0.01** | **0.04** | **p <0.01** | **0.01** | **p <0.01** | **0.01** |
| FADD S194 | **p <0.01** | 0.83 | **0.05** | **0.04** | **0.02** | **p <0.01** | **p <0.01** | 0.10 |
| FAK Y576/577 | 0.18 | **p <0.01** | **p <0.01** | **0.01** | **0.04** | **0.02** | **0.02** | 0.36 |
| FOXM1 T600 | **0.02** | **p <0.01** | **0.05** | **0.03** | **0.01** | 0.34 | **p <0.01** | **0.04** |
| FOXO1 S256 | **p <0.01** | 0.21 | **0.01** | 0.18 | **p <0.01** | 0.48 | **0.05** | 0.10 |
| FOXO1 T24/FOXO3 T32 | **p <0.01** | **0.04** | **p <0.01** | **p <0.01** | **0.05** | 0.10 | 0.12 | **p <0.01** |
| FOXO3 S253 | **0.02** | 0.10 | 0.14 | **0.04** | 0.10 | **p <0.01** | **0.02** | 0.09 |
| Grb2 | **p <0.01** | **0.01** | 0.28 | **0.01** | **0.03** | 0.58 | **0.02** | **0.02** |
| GSK-3 alpha/beta S21/9 | **p <0.01** | **p <0.01** | **0.02** | **0.01** | **p <0.01** | **p <0.01** | **p <0.01** | **p <0.01** |
| HDAC 1 | **p <0.01** | **0.01** | 0.08 | **0.01** | **0.02** | **0.01** | **p <0.01** | 0.15 |
| HDAC 3 | 0.11 | **0.02** | **p <0.01** | 0.06 | 0.06 | **0.02** | 0.08 | 0.12 |
| HDAC 4 | **p <0.01** | **0.02** | 0.13 | **0.02** | **0.01** | 0.89 | **0.01** | 0.38 |
| HDAC 6 | **p <0.01** | **p <0.01** | 0.36 | **0.03** | 0.17 | 0.76 | **0.01** | 0.10 |
| HER2 | **p <0.01** | 0.32 | 0.08 | **0.02** | 0.14 | **0.04** | **0.05** | **0.05** |
| HER2 Y1248 | **p <0.01** | 0.10 | 0.10 | 0.32 | **0.02** | **p <0.01** | **0.01** | **0.03** |
| HER3 Y1197 | **0.01** | 0.08 | 0.70 | 0.67 | **0.02** | 0.16 | 0.06 | **p <0.01** |
| HER3 Y1289 | 0.07 | **0.02** | 0.13 | 0.37 | 0.07 | 0.12 | **0.01** | **0.01** |
| Histone H3 S10 Mitosis Marker | **0.01** | **0.01** | 0.16 | **0.03** | **0.01** | 0.65 | **p <0.01** | **0.02** |
| Histone H3 S28 | **p <0.01** | **0.04** | 0.10 | **0.02** | **0.03** | **0.03** | **0.03** | **0.04** |
| Histone H3, Acetyl Lys9 14 | **p <0.01** | **p <0.01** | **0.03** | 0.07 | **p <0.01** | **p <0.01** | **p <0.01** | 0.08 |
| Histone H3, Di-Methyl Lys9 | **0.05** | **0.03** | **p <0.01** | 0.08 | **p <0.01** | **p <0.01** | **0.01** | 0.14 |
| Histone H3, Pan-Methyl Lys9 | 0.11 | **0.01** | 0.13 | 0.06 | **p <0.01** | **0.01** | **p <0.01** | **0.02** |
| Histone H4 Acetyl Lys8 | 0.08 | **0.03** | **0.05** | **0.04** | **p <0.01** | **0.01** | **0.03** | 0.20 |
| HSP27 S82 | **p <0.01** | **p <0.01** | **p <0.01** | **p <0.01** | **p <0.01** | **p <0.01** | **0.04** | 0.09 |
| HSP90a T5/7 | **p <0.01** | 0.07 | 0.11 | **0.02** | **p <0.01** | **0.02** | **0.02** | **0.02** |
| IGF-1R Y1131/IR Y1146 | **0.01** | 0.17 | **0.01** | **0.02** | **p <0.01** | 0.16 | **0.04** | **0.01** |
| IGF-1R Y1135/1136 IR Y1150/1151 | **p <0.01** | **p <0.01** | 0.44 | **0.01** | **0.04** | 0.06 | **0.01** | **p <0.01** |
| IkappaB-alpha S32/36 | 0.06 | **0.01** | **0.01** | **0.02** | 0.18 | **0.02** | 0.06 | 0.15 |
| IL-10 | 0.18 | **0.02** | 0.80 | **0.01** | 0.15 | 0.11 | 0.26 | 0.07 |
| IL-11 | 0.14 | **0.05** | 0.38 | **0.04** | **0.02** | 0.09 | 0.06 | 0.54 |
| IL-8 | **0.05** | **0.01** | 0.07 | 0.09 | **p <0.01** | 0.20 | 0.10 | 0.11 |
| Insulin Receptor beta | **p <0.01** | **0.02** | 0.22 | **0.03** | 0.21 | 0.08 | **0.05** | 0.28 |
| IRS-1 S612 | **p <0.01** | **p <0.01** | **0.01** | **0.02** | 0.12 | **0.02** | **p <0.01** | **p <0.01** |

| | | | | | | | | |
|---|---|---|---|---|---|---|---|---|
| Jak1 Y1022/1023 | **0.03** | **0.02** | 0.25 | 0.10 | **p <0.01** | 0.41 | 0.27 | 0.37 |
| Ki67 | 0.12 | 0.50 | **0.01** | 0.19 | **0.05** | **0.05** | **0.02** | 0.23 |
| LC3B | **p <0.01** | 0.06 | 0.33 | **0.03** | **p <0.01** | 0.09 | **0.03** | 0.14 |
| Lck Y505 | 0.08 | **0.02** | 0.81 | **0.02** | **0.03** | 0.17 | **0.04** | 0.17 |
| LIMK1 T508/LIMK2 T505 | **p <0.01** | **0.02** | 0.40 | **0.03** | 0.19 | 0.06 | 0.08 | **0.02** |
| LKB1 S334 | **p <0.01** | **p <0.01** | 0.53 | **0.03** | **0.04** | 0.07 | **0.02** | **0.02** |
| LKB1 S428 | 0.43 | **0.02** | **p <0.01** | **0.03** | **p <0.01** | **0.04** | **p <0.01** | **0.04** |
| MARCKS S152/156 | **p <0.01** | **p <0.01** | **0.04** | **0.02** | **0.05** | **p <0.01** | **p <0.01** | **p <0.01** |
| MDM2 S166 | 0.24 | **0.02** | 0.12 | 0.08 | **0.01** | 0.13 | **0.02** | **0.02** |
| MEK 1/2 | **p<0.018** | 0.11 | 0.08 | **0.01** | **0.01** | **0.01** | 0.11 | 0.07 |
| MEK 1/2 S217/221 | **0.01** | **p <0.01** | **p <0.01** | **0.03** | **p <0.01** | **0.04** | **0.02** | **0.02** |
| Met Y1234/1235 | **p <0.01** | **p <0.01** | **p <0.01** | **0.04** | 0.10 | **0.02** | **0.01** | **p <0.01** |
| MSK1 S360 | **0.02** | **0.01** | 0.08 | **0.03** | **0.04** | **p <0.01** | **0.01** | **p <0.01** |
| mTOR | 0.07 | **0.04** | 0.34 | **p<0.01** | **0.02** | **0.04** | **0.01** | 0.09 |
| mTOR S2448 | **0.03** | **0.02** | **0.02** | 0.29 | **p <0.01** | 0.09 | **p <0.01** | **0.02** |
| NF-kappaB p65 S536 | **0.03** | **p <0.01** | **0.03** | **0.03** | 0.06 | 0.09 | **0.04** | **p <0.01** |
| p27 Kip1 | **p <0.01** | **0.05** | **p <0.01** | **p <0.01** | **0.03** | **p <0.01** | **0.02** | **0.01** |
| p27 T187 | **p <0.01** | **p <0.01** | **p <0.01** | **0.04** | **0.01** | 0.24 | **0.05** | **0.04** |
| p38 MAP Kinase T180/Y182 | **p <0.01** | **p <0.01** | **p <0.01** | **0.02** | **p <0.01** | **0.02** | **p <0.01** | **p <0.01** |
| p53 | **p <0.01** | **0.01** | **0.02** | **0.01** | **p <0.01** | **0.02** | 0.07 | 0.17 |
| p53 S15 | **0.01** | **0.03** | **0.05** | **0.02** | **p <0.01** | 0.06 | 0.36 | 0.25 |
| p62/SQSTM1 | **0.01** | **p <0.01** | 0.20 | **0.02** | **p <0.01** | **0.02** | **p <0.01** | **0.02** |
| p70 S6 Kinase S371 | **p <0.01** | 0.10 | 0.33 | **p <0.01** | **p <0.01** | **0.03** | **p <0.01** | 0.53 |
| p70 S6 Kinase T389 | **p <0.01** | **p <0.01** | **0.04** | **0.01** | **0.02** | **0.03** | **p <0.01** | **p <0.01** |
| p70 S6 Kinase T412 | **p <0.01** | **p <0.01** | **p <0.01** | **0.02** | **0.01** | 0.09 | **p <0.01** | **0.01** |
| p90RSK S380 | **p <0.01** | **0.01** | **p <0.01** | **0.02** | **0.01** | **0.03** | **p <0.01** | **p <0.01** |
| p90RSK T359/S363 | **0.04** | **p <0.01** | **p <0.01** | **0.04** | **p <0.01** | **0.01** | **p <0.01** | **0.01** |
| PAK1 S199/204 PAK2 S192/197 | 0.06 | 0.13 | **p <0.01** | 0.43 | 0.16 | 0.14 | 0.14 | 0.07 |
| PAK1 T423/PAK2 T402 | **p <0.01** | **p <0.01** | 0.07 | 0.12 | 0.08 | 0.19 | **0.01** | **0.02** |
| PARP, cleaved (D214) | **0.02** | **0.01** | 0.09 | 0.07 | 0.07 | **0.01** | **0.03** | 0.16 |
| Paxillin Y118 | **0.01** | **p <0.01** | **p <0.01** | 0.13 | **p <0.01** | **p <0.01** | **p <0.01** | **p <0.01** |
| PDGF Receptor beta Y751 | **0.05** | **p <0.01** | **0.04** | **0.04** | **p <0.01** | 0.31 | 0.07 | **p <0.01** |
| PDK1 S241 | **p <0.01** | **p <0.01** | **0.02** | **0.02** | **0.01** | **p <0.01** | **0.02** | 0.06 |
| PDL1 E1L3N | **0.02** | **0.01** | 0.43 | **0.01** | **0.02** | 0.62 | **0.01** | 0.11 |
| PI3 Kinase p110gamma | **0.04** | **0.02** | 0.10 | 0.07 | 0.08 | **0.04** | 0.21 | **p <0.01** |
| PIAS1 | **p <0.01** | **p <0.01** | **0.01** | **0.04** | **0.01** | 0.67 | **0.02** | **0.05** |
| PKA C T197 | **p <0.01** | **p <0.01** | 0.17 | **p <0.01** | 0.09 | 0.10 | 0.39 | 0.08 |
| PKC a/BII T638/641 | **0.01** | 0.43 | 0.51 | **0.02** | 0.10 | 0.11 | **0.02** | 0.13 |
| PKC alpha S657 | **0.01** | **0.02** | 0.71 | **0.03** | 0.38 | 0.07 | 0.06 | 0.17 |
| PKC delta T505 | **0.01** | **p <0.01** | 0.30 | **0.04** | 0.12 | **0.02** | 0.33 | 0.14 |
| PKC theta T538 | **p <0.01** | **0.02** | 0.98 | 0.11 | **0.04** | 0.28 | **p <0.01** | **0.05** |
| PKC zeta/lambda T410/403 | **0.03** | **0.02** | 0.09 | **0.02** | **0.05** | 0.38 | **p <0.01** | **p <0.01** |
| PLCgamma1 Y783 | 0.11 | 0.08 | 0.09 | 0.07 | 0.78 | 0.08 | 0.17 | **0.02** |
| PLK1 T210 | 0.12 | **p <0.01** | 0.35 | **0.01** | 0.21 | 0.17 | 0.09 | 0.08 |
| PP2A a Subunit | 0.06 | **0.02** | 0.12 | **0.05** | **0.01** | **p <0.01** | **0.01** | 0.13 |
| PP2A B Subunit | **0.02** | **0.02** | 0.08 | 0.08 | **0.02** | **0.03** | 0.08 | 0.09 |
| PRAS40 T246 | **p <0.01** | **p <0.01** | **0.02** | 0.19 | **0.03** | **p <0.01** | **p <0.01** | **p <0.01** |
| PTEN | **p <0.01** | **0.01** | 0.14 | 0.10 | 0.07 | **0.02** | 0.12 | 0.25 |
| PTEN S380 | **p <0.01** | **p <0.01** | 0.10 | 0.06 | 0.10 | 0.83 | 0.09 | **0.05** |
| Pyk2 Y402 | **0.01** | **p <0.01** | **p <0.01** | **0.02** | **0.02** | **0.01** | **p <0.01** | **p <0.01** |
| Raf S259 | **p <0.01** | **p <0.01** | 0.18 | **0.02** | **0.02** | **0.03** | **0.01** | 0.07 |
| Ras-GRF1 | **p <0.01** | **0.04** | 0.47 | 0.06 | **0.02** | **0.01** | 0.06 | **0.05** |
| Ras-GRF1 S916 | **p <0.01** | **0.02** | **0.02** | **0.01** | **p <0.01** | **0.04** | **p <0.01** | 0.09 |
| Rb S780 | **0.02** | 0.13 | 0.14 | **0.03** | **p <0.01** | 0.61 | 0.09 | 0.07 |
| Ret Y905 | **p <0.01** | **p <0.01** | 0.40 | 0.18 | **p <0.01** | 0.59 | 0.42 | **0.01** |
| Ron Y1353 | **0.02** | 0.06 | **0.03** | **0.02** | **0.02** | **0.02** | **p <0.01** | 0.76 |
| RSK3 T356/S360 | **0.02** | **0.01** | **p <0.01** | **0.03** | **p <0.01** | **p <0.01** | **p <0.01** | **p <0.01** |
| S6 Ribosomal Protein S235/236 | 0.16 | **p <0.01** | 0.07 | 0.09 | **p <0.01** | **p <0.01** | **0.01** | **0.03** |
| S6 Ribosomal Protein S240/244 | **0.04** | **p <0.01** | 0.11 | 0.14 | **p <0.01** | **p <0.01** | **p <0.01** | **0.03** |

| | | | | | | | | |
|---|---|---|---|---|---|---|---|---|
| SAPK/JNK T183/Y185 | **p <0.01** | **p <0.01** | **0.04** | **0.02** | **p <0.01** | **p <0.01** | **p <0.01** | **p <0.01** |
| SEK1/MKK4 S80 | **0.02** | **0.02** | **p <0.01** | **0.02** | **0.03** | 0.22 | **p <0.01** | **p <0.01** |
| SGK1 S78 | **0.01** | 0.10 | 0.14 | **0.04** | **0.03** | **0.02** | **0.02** | 0.19 |
| Shc Y317 | **p <0.01** | **p <0.01** | **p <0.01** | 0.16 | **p <0.01** | **0.04** | **0.05** | **0.02** |
| SHIP1 Y1020 | **0.02** | **p <0.01** | **0.04** | **0.01** | **p <0.01** | **0.03** | **p <0.01** | **p <0.01** |
| SHP2 Y580 | **0.05** | 0.06 | 0.12 | **0.03** | **0.05** | **0.05** | 0.09 | **p <0.01** |
| Smad2 S245/250/255 | 0.09 | **p <0.01** | 0.10 | **0.01** | **p <0.01** | 0.08 | **p <0.01** | **0.02** |
| Smad2 S465/467 | 1.00 | **0.02** | 0.99 | **0.03** | 1.00 | 0.48 | 0.32 | 1.00 |
| SOCS1 | 0.25 | 0.18 | **0.02** | 0.22 | 0.13 | **0.02** | **0.03** | 0.49 |
| SOCS3 | 0.06 | 0.09 | **0.03** | **0.01** | **0.01** | **p <0.01** | **p <0.01** | **0.04** |
| Src Family Y416 | **p <0.01** | 0.17 | 0.07 | **0.01** | **0.02** | 0.14 | 0.08 | **0.02** |
| Src Y527 | **p <0.01** | **0.01** | 0.48 | **p <0.01** | 0.12 | **0.02** | **0.02** | **p <0.01** |
| Stat1 Y701 | 0.62 | **0.02** | 0.47 | **0.02** | **0.03** | 0.71 | **0.01** | 0.08 |
| Stat2 Y690 | 0.23 | **0.05** | **0.02** | 0.18 | 0.18 | 0.14 | 0.13 | 0.07 |
| Stat3 S727 | **0.05** | **p <0.01** | **0.04** | **p <0.01** | **p <0.01** | 0.27 | **0.01** | **p <0.01** |
| Stat3 Y705 | **p <0.01** | **p <0.01** | **p <0.01** | **p <0.01** | **p <0.01** | **p <0.01** | **p <0.01** | 0.26 |
| Stat4 Y693 | **p <0.01** | **p <0.01** | 0.84 | 0.16 | **0.02** | 0.10 | 0.11 | 0.11 |
| Stat5 Y694 | **p <0.01** | **0.03** | 0.07 | **0.01** | **0.03** | 0.30 | **p <0.01** | **p <0.01** |
| Stat6 Y641 | **p <0.01** | **p <0.01** | 0.22 | 0.07 | **p <0.01** | 0.14 | **0.01** | **p <0.01** |
| Survivin | **p <0.01** | 0.80 | 0.16 | **0.05** | **0.05** | **0.02** | **0.01** | 0.10 |
| Syk Y525/526 | **0.04** | **p <0.01** | **p <0.01** | 0.19 | 0.53 | **p <0.01** | **0.04** | **0.01** |
| TGF-Beta | **0.02** | **p <0.01** | 0.28 | 0.07 | **0.05** | 0.97 | **p <0.01** | **0.03** |
| TNF alpha | **0.01** | **0.03** | 0.06 | **p <0.01** | 0.13 | **0.04** | **p <0.01** | 0.15 |
| TNF-R1 | **p <0.01** | **p <0.01** | 0.28 | **0.01** | **p <0.01** | **0.05** | **p <0.01** | 0.11 |
| Tubulin alpha | **p <0.01** | 0.07 | 0.27 | 0.07 | **0.03** | 0.28 | **0.02** | 0.07 |
| Tyk2 | **p <0.01** | **p <0.01** | 0.26 | **0.02** | **0.03** | **p <0.01** | **p <0.01** | **0.02** |
| VASP S157 | 0.35 | **p <0.01** | **0.01** | **p <0.01** | **p <0.01** | **0.03** | 0.35 | 0.20 |
| XIAP | **p <0.01** | **p <0.01** | 0.08 | **0.02** | **p <0.01** | 0.22 | **0.01** | **p <0.01** |
| YAP S127 | **p <0.01** | 0.24 | 0.07 | **p <0.01** | **p <0.01** | **0.01** | **p <0.01** | **p <0.01** |
| **Proteins (C3)** | **A549** | **Calu-3** | **H1734** | **H1838** | **H2122** | **H23** | **H358** | **H522** |
| 4E-BP1 S65 | **p <0.01** | 0.12 | 0.72 | 0.20 | **p <0.01** | **p <0.01** | **0.02** | 0.10 |
| 4E-BP1 T70 | **p <0.01** | **0.01** | **0.02** | 0.06 | **p <0.01** | **p <0.01** | **p <0.01** | **p <0.01** |
| Acetyl-CoA Carboxylase S79 | **p <0.01** | **p <0.01** | **0.01** | **0.02** | **p <0.01** | **0.05** | **p <0.01** | **0.01** |
| AKT | **p <0.01** | **0.02** | **0.02** | 0.66 | **0.04** | **0.03** | **0.02** | **0.01** |
| AKT S473 | **p <0.01** | **p <0.01** | **p <0.01** | **0.02** | **p <0.01** | **p <0.01** | **p <0.01** | **p <0.01** |
| Alk | **0.03** | **p <0.01** | 0.35 | 0.11 | **p <0.01** | **0.02** | 0.19 | 0.21 |
| Alk Y1604 | **0.03** | **0.02** | **p <0.01** | **0.02** | **p <0.01** | 0.13 | **0.04** | 0.12 |
| AMPK alpha T172 | 0.16 | **0.01** | 0.58 | 0.15 | **0.01** | 0.92 | 0.08 | 0.37 |
| AMPK alpha1 S485 | **p <0.01** | 0.27 | **0.01** | 0.25 | **0.05** | 0.07 | 0.38 | **p <0.01** |
| AMPK beta1 S108 | 0.13 | **0.02** | **p <0.01** | **0.02** | **p <0.01** | 0.08 | 0.31 | **p <0.01** |
| Androgen Receptor S81 | **p <0.01** | 0.19 | **0.04** | 0.50 | **0.05** | **0.01** | **p <0.01** | **0.03** |
| A-Raf S299 | **p <0.01** | **p <0.01** | **0.03** | 0.62 | **p <0.01** | 0.07 | 0.33 | 0.51 |
| ATF-2 T69/71 | 0.11 | 0.34 | **0.03** | **0.03** | 0.57 | 0.07 | 0.29 | 0.09 |
| ATG12 | **0.04** | 0.06 | **0.04** | 0.27 | 0.41 | 0.25 | 0.82 | **0.02** |
| ATG5 | 0.08 | **0.04** | **0.05** | 0.08 | 0.07 | 0.11 | **0.02** | **0.01** |
| ATM S1981 | **0.05** | **0.02** | 0.26 | **0.02** | 0.30 | **0.01** | **0.03** | **0.02** |
| ATP-Citrate Lyase S454 | **0.03** | 0.43 | **p <0.01** | 0.40 | **0.05** | **0.02** | 0.60 | 0.06 |
| ATR S428 | 0.16 | **0.05** | 0.12 | **0.03** | **p <0.01** | **0.01** | **0.01** | **0.01** |
| Aurora A T288/Aurora B T232/Aurora C T198 | **0.01** | **p <0.01** | **p <0.01** | 0.58 | 0.52 | 0.10 | 0.10 | **0.05** |
| Axl Y702 | **p <0.01** | **p <0.01** | **0.01** | **0.01** | **p <0.01** | **0.02** | **0.03** | 0.15 |
| BAD S112 | **0.02** | **p <0.01** | **0.03** | **0.02** | **p <0.01** | **0.03** | **0.01** | **p <0.01** |
| BAD S136 | **0.03** | 0.29 | **0.04** | **0.01** | **0.03** | 0.08 | **p <0.01** | **0.03** |
| BAD S155 | **0.05** | **0.03** | 0.30 | 0.35 | **0.03** | **0.02** | 0.38 | **0.01** |
| Bak | **p <0.01** | **p <0.01** | 0.60 | 0.15 | **p <0.01** | 0.09 | 0.07 | **0.02** |
| Bax | **p <0.01** | **0.01** | 0.14 | 0.13 | 0.12 | 0.52 | 0.11 | **0.02** |
| Bcl-2 S70 | **p <0.01** | 0.31 | 0.39 | **0.02** | **p <0.01** | **p <0.01** | **0.02** | **p <0.01** |
| Bcl-2 T56 | 0.06 | **p <0.01** | **p <0.01** | 0.07 | 0.08 | **p <0.01** | 0.20 | **0.03** |
| Bcl-xL | 0.67 | **0.04** | 0.25 | **p <0.01** | 0.09 | **0.03** | 0.45 | **p <0.01** |

| | | | | | | | | |
|---|---|---|---|---|---|---|---|---|
| Beclin1 | **p <0.01** | 0.12 | 0.07 | **0.04** | **0.05** | 0.40 | **0.04** | **0.01** |
| BIM | **p <0.01** | **0.02** | **0.01** | **0.01** | **p <0.01** | **0.04** | **0.02** | **0.01** |
| B-Raf S445 | **0.03** | **p <0.01** | **p <0.01** | 0.66 | 0.08 | **0.01** | 0.15 | 0.35 |
| c-Abl T735 | 0.13 | **0.03** | **0.03** | 0.44 | **p <0.01** | **p <0.01** | **0.01** | **p <0.01** |
| c-Abl Y245 | **p <0.01** | **p <0.01** | **0.04** | **0.02** | **p <0.01** | **p <0.01** | 0.07 | **p <0.01** |
| Caspase-3, cleaved (D175) | **0.02** | **p <0.01** | 0.15 | **0.01** | 0.31 | **0.02** | 0.24 | **p <0.01** |
| Caspase-6, cleaved (D162) | 0.06 | **0.04** | **0.03** | 0.14 | 0.17 | 0.17 | 0.25 | 0.31 |
| Caspase-7, cleaved (D198) | **0.03** | **p <0.01** | 0.14 | 0.06 | 0.08 | **p <0.01** | 0.09 | 0.27 |
| Caspase-9, cleaved (D330) | **0.01** | 0.34 | 0.12 | 0.07 | 0.09 | 0.13 | **0.01** | **0.02** |
| Catenin beta S33/37/T41 | 0.51 | **p <0.01** | **p <0.01** | **0.03** | 0.07 | **0.04** | **p <0.01** | **0.02** |
| Chk-1 S345 | 0.07 | **p <0.01** | **p <0.01** | 0.26 | **p <0.01** | **0.01** | 0.13 | 0.08 |
| Chk-2 S33/35 | **0.05** | 0.80 | 1.00 | 1.00 | 0.96 | 1.00 | 1.00 | 1.00 |
| c-Met | **0.01** | 0.12 | 0.14 | **0.03** | **0.05** | **0.03** | **0.01** | **p <0.01** |
| Cofilin S3 | **0.01** | **p <0.01** | **0.05** | **p <0.01** | **p <0.01** | 0.15 | **0.02** | 0.06 |
| Cox-2 | **p <0.01** | **0.03** | **0.04** | 0.08 | **p <0.01** | **0.03** | 0.56 | 0.52 |
| cPLA2 S505 | **0.01** | **0.01** | 0.08 | **0.02** | **p <0.01** | **0.01** | **p <0.01** | **0.02** |
| C-Raf S338 | **p <0.01** | 0.13 | 0.08 | **p <0.01** | **0.03** | **0.01** | **0.01** | **0.01** |
| CREB S133 | **0.01** | **p <0.01** | **p <0.01** | **0.02** | 0.07 | 0.70 | **0.02** | 0.08 |
| CrkL Y207 | **0.01** | **p <0.01** | **p <0.01** | 0.08 | **0.03** | **p <0.01** | **p <0.01** | **p <0.01** |
| Cyclin A2 | **0.05** | 0.23 | 0.91 | 0.35 | **p <0.01** | **0.01** | **0.04** | 0.23 |
| Cyclin B1 | **0.02** | 0.62 | **p <0.01** | 0.49 | **0.01** | **p <0.01** | **0.01** | **0.02** |
| Cyclin D1 | **0.03** | **0.04** | **p <0.01** | 0.09 | **0.01** | **0.03** | 0.22 | 0.42 |
| EGFR | 0.09 | **p <0.01** | **0.01** | 0.12 | 0.19 | **0.04** | **0.03** | **0.02** |
| EGFR Y1045 | 0.06 | **p <0.01** | **p <0.01** | 0.08 | **p <0.01** | 0.21 | **p <0.01** | **p <0.01** |
| EGFR Y1068 | **0.03** | **0.02** | **0.01** | 0.07 | **p <0.01** | 0.53 | 0.30 | 0.44 |
| EGFR Y1148 | **0.01** | **0.04** | 0.53 | **0.02** | **p <0.01** | 0.11 | **p <0.01** | 0.10 |
| EGFR Y1173 | **0.01** | **p <0.01** | **0.05** | 0.22 | **p <0.01** | 0.11 | 0.25 | **0.03** |
| eIF4E S209 | **p <0.01** | 0.26 | **0.03** | 0.07 | **0.01** | 0.14 | **0.02** | **0.04** |
| eIF4G S1108 | **p <0.01** | 0.06 | 0.20 | **0.04** | 0.14 | **0.01** | **p <0.01** | **p <0.01** |
| Elk-1 S383 | **0.04** | **p <0.01** | **p <0.01** | 0.18 | 0.30 | **0.02** | **p <0.01** | **p <0.01** |
| eNOS S113 | 0.09 | **p <0.01** | 0.08 | 0.10 | 0.22 | **p <0.01** | 0.07 | 0.14 |
| eNOS/NOS III S116 | **0.03** | **p <0.01** | 0.10 | **0.02** | 0.43 | **0.05** | **p <0.01** | **0.03** |
| Ephrin A3 Y799/A4 Y799/A5 Y833 | 0.59 | **0.04** | 0.48 | 0.27 | **p <0.01** | **p <0.01** | 0.29 | 0.18 |
| ERK 1/2 | 0.70 | 0.08 | 0.16 | 0.43 | **0.04** | **0.03** | 0.21 | 0.33 |
| ERK 1/2 T202/Y204 | **p <0.01** | **p <0.01** | **p <0.01** | **p <0.01** | **p <0.01** | **p <0.01** | **p <0.01** | **p <0.01** |
| Estrogen Receptor alpha | **0.01** | 0.10 | **0.01** | 0.25 | **0.02** | 0.08 | 0.24 | **0.01** |
| Estrogen Receptor alpha S118 | **0.02** | 0.07 | **0.01** | 0.13 | 0.07 | 0.07 | **0.04** | **p <0.01** |
| Etk Y40 | **0.04** | **p <0.01** | **0.02** | **0.02** | **p <0.01** | **p <0.01** | **0.04** | 0.12 |
| Ezrin T567/Radixin T564/Moesin T558 | **0.02** | **p <0.01** | **p <0.01** | **0.01** | **p <0.01** | **0.01** | **0.01** | **0.01** |
| FADD S194 | **p <0.01** | 0.10 | 0.13 | 0.06 | **p <0.01** | 0.12 | **0.01** | **0.01** |
| FAK Y576/577 | **0.05** | **p <0.01** | **p <0.01** | **0.01** | **0.05** | 0.12 | **0.02** | 0.10 |
| FOXM1 T600 | **0.04** | **0.02** | 0.10 | **0.04** | **p <0.01** | 0.09 | **0.02** | **p <0.01** |
| FOXO1 S256 | **0.03** | **p <0.01** | **p <0.01** | 0.26 | **p <0.01** | 0.15 | 0.54 | **0.04** |
| FOXO1 T24/FOXO3 T32 | **p <0.01** | 0.45 | **p <0.01** | **0.01** | 0.41 | **0.03** | **0.02** | **p <0.01** |
| FOXO3 S253 | 0.12 | **0.05** | 0.19 | 0.11 | **0.01** | **0.03** | **0.04** | **0.02** |
| Grb2 | **p <0.01** | 0.18 | 0.50 | 0.21 | **p <0.01** | 0.31 | 0.06 | **0.01** |
| GSK-3 alpha/beta S21/9 | **p <0.01** | 0.06 | **0.01** | **0.05** | **p <0.01** | **0.02** | **p <0.01** | **p <0.01** |
| HDAC 1 | **0.01** | **p <0.01** | **p <0.01** | **0.02** | **p <0.01** | **p <0.01** | 0.09 | 0.98 |
| HDAC 3 | **p <0.01** | **p <0.01** | **p <0.01** | **0.02** | **0.01** | **p <0.01** | **p <0.01** | **0.01** |
| HDAC 4 | 0.24 | **p <0.01** | **0.03** | **0.05** | 0.08 | 0.32 | 0.14 | **p <0.01** |
| HDAC 6 | **p <0.01** | **p <0.01** | 0.28 | 0.07 | 0.07 | 0.18 | 0.06 | **0.01** |
| HER2 | **p <0.01** | 0.33 | **0.05** | **0.04** | 0.07 | **0.01** | 0.10 | **0.05** |
| HER2 Y1248 | **0.05** | **0.02** | 0.09 | **0.03** | **0.04** | **p <0.01** | **0.05** | 0.16 |
| HER3 Y1197 | **0.01** | **p <0.01** | 0.45 | 0.69 | **0.02** | 0.54 | **0.03** | **0.03** |
| HER3 Y1289 | **p <0.01** | **p <0.01** | 0.36 | **0.02** | **0.04** | **0.01** | **p <0.01** | **p <0.01** |
| Histone H3 S10 Mitosis Marker | **0.03** | **p <0.01** | **0.03** | 0.46 | **p <0.01** | 0.15 | **0.05** | **p <0.01** |
| Histone H3 S28 | 0.07 | **p <0.01** | 0.15 | 0.14 | 0.06 | **p <0.01** | 0.07 | **0.01** |
| Histone H3, Acetyl Lys9 14 | **p <0.01** | **p <0.01** | **0.03** | 0.06 | **p <0.01** | **p <0.01** | **p <0.01** | **0.05** |

| | | | | | | | | |
|---|---|---|---|---|---|---|---|---|
| Histone H3, Di-Methyl Lys9 | **p <0.01** | **p <0.01** | **p <0.01** | 0.06 | 0.06 | **p <0.01** | **0.02** | 0.06 |
| Histone H3, Pan-Methyl Lys9 | **p <0.01** | **p <0.01** | **0.01** | **0.05** | 0.22 | 0.06 | 0.12 | 0.06 |
| Histone H4 Acetyl Lys8 | **p <0.01** | **p <0.01** | **0.01** | **0.03** | **p <0.01** | **0.03** | **0.01** | 0.14 |
| HSP27 S82 | **p <0.01** | **p <0.01** | **p <0.01** | **0.01** | **p <0.01** | **0.02** | **p <0.01** | **0.02** |
| HSP90a T5/7 | **0.04** | **p <0.01** | **0.02** | **0.02** | **p <0.01** | **p <0.01** | 0.08 | **0.03** |
| IGF-1R Y1131/IR Y1146 | **0.05** | **0.01** | **0.02** | 0.54 | **p <0.01** | 0.11 | 0.18 | **0.03** |
| IGF-1R Y1135/1136 IR Y1150/1151 | **p <0.01** | **p <0.01** | **0.05** | **0.02** | **p <0.01** | **0.02** | 0.46 | 0.14 |
| IkappaB-alpha S32/36 | **p <0.01** | 0.21 | **0.03** | **0.02** | **0.04** | **0.02** | 0.14 | **p <0.01** |
| IL-10 | 0.65 | **0.03** | 0.19 | 0.13 | 0.09 | 0.12 | 0.51 | **0.02** |
| IL-11 | **0.02** | **p <0.01** | 0.11 | 0.15 | **p <0.01** | 0.35 | **0.04** | 0.80 |
| IL-8 | **p <0.01** | **0.02** | **0.01** | 0.43 | **p <0.01** | 0.16 | **0.04** | 0.92 |
| Insulin Receptor beta | 0.06 | 0.06 | **0.05** | 0.73 | 0.09 | 0.15 | 0.23 | 0.09 |
| IRS-1 S612 | **p <0.01** | 0.15 | **p <0.01** | 0.51 | 0.13 | **0.02** | **p <0.01** | **p <0.01** |
| Jak1 Y1022/1023 | 0.07 | **0.01** | 0.65 | 0.51 | **p <0.01** | 0.56 | 0.41 | 0.52 |
| Ki67 | 0.15 | 0.13 | **0.02** | 0.13 | **0.01** | **p <0.01** | **0.02** | **0.02** |
| LC3B | 0.08 | 0.06 | **p <0.01** | **0.04** | 0.15 | 0.08 | 0.18 | 0.16 |
| Lck Y505 | 0.27 | **0.05** | 0.34 | 0.22 | **0.02** | **0.02** | 0.08 | 0.07 |
| LIMK1 T508/LIMK2 T505 | **0.02** | **p <0.01** | 0.10 | 0.06 | **0.03** | 0.22 | 0.27 | **0.03** |
| LKB1 S334 | 0.20 | **p <0.01** | 0.29 | 0.39 | **p <0.01** | 0.27 | 0.61 | 0.22 |
| LKB1 S428 | **p <0.01** | **0.01** | **0.03** | 0.13 | **p <0.01** | **0.05** | 0.06 | **0.02** |
| MARCKS S152/156 | **p <0.01** | **p <0.01** | **0.01** | **p <0.01** | 0.44 | 0.28 | **p <0.01** | **p <0.01** |
| MDM2 S166 | 0.08 | **0.05** | 0.24 | **0.03** | 0.19 | **0.03** | **p <0.01** | **0.01** |
| MEK 1/2 | **p<0.01** | **0.01** | **0.03** | **0.01** | **p<0.016** | **p<0.01** | **p<0.01** | **p<0.01** |
| MEK 1/2 S217/221 | **p <0.01** | 0.74 | **p <0.01** | 0.09 | **p <0.01** | 0.12 | **0.01** | **p <0.01** |
| Met Y1234/1235 | **p <0.01** | **p <0.01** | **0.04** | **0.03** | **p <0.01** | 0.08 | **0.01** | **p <0.01** |
| MSK1 S360 | **0.02** | 0.87 | **p <0.01** | 0.78 | 0.06 | 0.10 | **p <0.01** | **p <0.01** |
| mTOR | **p<0.013** | 0.05 | **0.04** | **0.03** | 0.64 | **0.02** | **p<0.01** | **0.01** |
| mTOR S2448 | **0.01** | 0.12 | **p <0.01** | 0.20 | **p <0.01** | 0.12 | **0.01** | **p <0.01** |
| NF-kappaB p65 S536 | **p <0.01** | **p <0.01** | **p <0.01** | **0.01** | **p <0.01** | **0.04** | **0.01** | 0.11 |
| p27 Kip1 | **p <0.01** | 0.08 | **p <0.01** | **0.02** | 0.42 | **p <0.01** | **0.05** | **p <0.01** |
| p27 T187 | **p <0.01** | **p <0.01** | **p <0.01** | 0.20 | **p <0.01** | **0.05** | **0.02** | **0.03** |
| p38 MAP Kinase T180/Y182 | **p <0.01** | **p <0.01** | **p <0.01** | **0.04** | **p <0.01** | **p <0.01** | **0.03** | **p <0.01** |
| p53 | 0.11 | **p <0.01** | 0.07 | **0.01** | **p <0.01** | **p <0.01** | **p <0.01** | 0.14 |
| p53 S15 | **0.02** | **0.02** | 0.07 | **0.01** | **0.05** | **p <0.01** | 0.12 | 0.32 |
| p62/SQSTM1 | **0.01** | **p <0.01** | **0.01** | **0.02** | **p <0.01** | 0.07 | **0.01** | 0.55 |
| p70 S6 Kinase S371 | **0.01** | **p <0.01** | 0.15 | **0.02** | **p <0.01** | 0.23 | **p <0.01** | 0.08 |
| p70 S6 Kinase T389 | **p <0.01** | **p <0.01** | **0.01** | **0.01** | **p <0.01** | **0.02** | **p <0.01** | **0.01** |
| p70 S6 Kinase T412 | **p <0.01** | **p <0.01** | **p <0.01** | 0.59 | **0.03** | 0.64 | **0.01** | **0.01** |
| p90RSK S380 | 0.06 | **0.02** | **p <0.01** | **0.01** | **p <0.01** | **p <0.01** | **p <0.01** | **p <0.01** |
| p90RSK T359/S363 | 0.25 | 0.07 | **p <0.01** | 0.81 | **0.01** | **p <0.01** | **0.04** | **p <0.01** |
| PAK1 S199/204 PAK2 S192/197 | **p <0.01** | **0.02** | **p <0.01** | 0.06 | **0.02** | **0.03** | **0.04** | **0.01** |
| PAK1 T423/PAK2 T402 | **0.01** | **p <0.01** | **0.02** | 0.69 | **p <0.01** | 0.38 | **0.03** | 0.11 |
| PARP, cleaved (D214) | **0.05** | **p <0.01** | **0.01** | 0.07 | **p <0.01** | **p <0.01** | **0.04** | 0.13 |
| Paxillin Y118 | 0.19 | **0.04** | **p <0.01** | **0.05** | **p <0.01** | **p <0.01** | **0.01** | **p <0.01** |
| PDGF Receptor beta Y751 | **0.03** | 0.06 | 0.07 | **0.04** | **p <0.01** | 0.51 | **0.03** | **p <0.01** |
| PDK1 S241 | **p <0.01** | **0.04** | **0.03** | **0.03** | **p <0.01** | **p <0.01** | **0.03** | **p <0.01** |
| PDL1 E1L3N | **p <0.01** | **p <0.01** | **0.01** | 0.13 | **p <0.01** | 0.43 | **0.03** | 0.63 |
| PI3 Kinase p110gamma | **p <0.01** | **0.02** | **0.02** | 0.47 | **p <0.01** | 0.21 | 0.15 | **0.05** |
| PIAS1 | 0.12 | **p <0.01** | **0.04** | **0.02** | **p <0.01** | 0.14 | **0.02** | **0.05** |
| PKA C T197 | **0.05** | **0.01** | 0.08 | 0.07 | **p <0.01** | 0.21 | **0.05** | **0.03** |
| PKC a/BII T638/641 | **0.01** | 0.30 | **0.04** | 0.59 | 0.42 | **0.03** | 0.25 | **0.05** |
| PKC alpha S657 | **0.03** | 0.08 | 0.34 | 0.10 | 0.20 | 0.06 | 0.35 | **0.02** |
| PKC delta T505 | 0.08 | **p <0.01** | **0.02** | 0.35 | **0.01** | 0.14 | 0.09 | 0.06 |
| PKC theta T538 | **0.01** | **p <0.01** | 0.08 | 0.27 | **p <0.01** | 0.24 | 0.18 | 0.11 |
| PKC zeta/lambda T410/403 | 0.10 | **p <0.01** | 0.06 | 0.08 | **p <0.01** | **0.04** | **0.04** | 0.07 |
| PLCgamma1 Y783 | 0.41 | 0.06 | **0.04** | 0.11 | 0.20 | 0.09 | 0.15 | 0.42 |
| PLK1 T210 | 0.40 | **p <0.01** | 0.09 | 0.11 | **0.04** | **0.02** | **0.05** | **0.03** |
| PP2A a Subunit | **0.02** | **p <0.01** | **0.03** | **p <0.01** | **0.01** | 0.08 | **0.02** | 0.21 |
| PP2A B Subunit | **p <0.01** | **p <0.01** | **p <0.01** | **0.02** | **0.01** | 0.32 | **0.02** | **p <0.01** |

| | | | | | | | | |
|---|---|---|---|---|---|---|---|---|
| PRAS40 T246 | **p <0.01** | **p <0.01** | **0.01** | 0.16 | 0.12 | **p <0.01** | **p <0.01** | **p <0.01** |
| PTEN | **p <0.01** | **0.05** | 0.20 | **0.03** | **0.05** | 0.09 | 0.36 | 0.62 |
| PTEN S380 | 0.11 | **0.01** | **0.04** | **0.05** | **0.01** | 0.87 | 0.16 | 0.09 |
| Pyk2 Y402 | **0.04** | **p <0.01** | **p <0.01** | **0.03** | **p <0.01** | **0.02** | **p <0.01** | **p <0.01** |
| Raf S259 | **p <0.01** | **0.04** | **0.04** | **0.03** | **0.01** | **0.01** | **p <0.01** | **p <0.01** |
| Ras-GRF1 | **0.05** | **0.01** | 0.07 | **0.04** | 0.10 | **p <0.01** | 0.13 | **0.02** |
| Ras-GRF1 S916 | **p <0.01** | **p <0.01** | 0.11 | **p <0.01** | **p <0.01** | **p <0.01** | **p <0.01** | 0.19 |
| Rb S780 | **p <0.01** | 0.09 | 0.29 | 0.06 | **0.01** | 0.14 | 0.06 | **0.02** |
| Ret Y905 | 0.06 | **p <0.01** | 0.22 | 0.82 | **p <0.01** | 0.58 | 0.41 | **0.04** |
| Ron Y1353 | **p <0.01** | **0.01** | 0.09 | 0.71 | **p <0.01** | **p <0.01** | 0.45 | 0.09 |
| RSK3 T356/S360 | **p <0.01** | **0.01** | **0.01** | **p <0.01** | **0.01** | **0.01** | **p <0.01** | **p <0.01** |
| S6 Ribosomal Protein S235/236 | **p <0.01** | **0.03** | **0.01** | **0.02** | **p <0.01** | **p <0.01** | **0.01** | **0.04** |
| S6 Ribosomal Protein S240/244 | **0.03** | **0.02** | **0.02** | 0.12 | **p <0.01** | **p <0.01** | **0.03** | 0.07 |
| SAPK/JNK T183/Y185 | **p <0.01** | **0.03** | 0.16 | 0.07 | **p <0.01** | **0.02** | **p <0.01** | **p <0.01** |
| SEK1/MKK4 S80 | **p <0.01** | **p <0.01** | **0.01** | **0.02** | **p <0.01** | 0.22 | **p <0.01** | 0.50 |
| SGK1 S78 | **0.03** | **0.03** | 0.21 | **0.01** | **p <0.01** | **p <0.01** | 0.08 | **0.02** |
| Shc Y317 | **p <0.01** | **p <0.01** | **p <0.01** | **0.03** | **p <0.01** | 0.33 | 0.13 | **p <0.01** |
| SHIP1 Y1020 | **0.04** | **p <0.01** | **0.03** | 0.16 | **p <0.01** | **0.01** | 0.11 | 0.34 |
| SHP2 Y580 | 0.78 | 0.27 | 0.13 | 0.17 | **0.03** | 0.93 | 0.43 | **p <0.01** |
| Smad2 S245/250/255 | 0.09 | 0.08 | **0.05** | 0.09 | **p <0.01** | 0.11 | **0.03** | **p <0.01** |
| Smad2 S465/467 | 1.00 | **0.05** | 0.99 | 0.76 | 1.00 | 0.07 | **0.02** | 1.00 |
| SOCS1 | **0.01** | 0.23 | 0.06 | 0.13 | 0.62 | **0.03** | 0.17 | **0.05** |
| SOCS3 | 0.21 | **p <0.01** | **p <0.01** | 0.13 | **p <0.01** | **p <0.01** | 0.80 | 0.45 |
| Src Family Y416 | **p <0.01** | 0.07 | **0.04** | **0.05** | **p <0.01** | 0.13 | **0.05** | 0.20 |
| Src Y527 | **p <0.01** | **0.05** | **0.02** | **0.01** | **0.02** | **p <0.01** | **p <0.01** | **p <0.01** |
| Stat1 Y701 | **p <0.01** | **0.03** | 0.21 | **0.03** | 0.77 | 0.37 | 0.59 | **p <0.01** |
| Stat2 Y690 | 0.83 | 0.08 | 0.26 | 0.14 | **0.04** | 0.15 | 0.10 | 0.86 |
| Stat3 S727 | **0.01** | **p <0.01** | **0.04** | **0.01** | 0.30 | 0.08 | 0.07 | **p <0.01** |
| Stat3 Y705 | **p <0.01** | **p <0.01** | **p <0.01** | **p <0.01** | **p <0.01** | **p <0.01** | **0.01** | 0.06 |
| Stat4 Y693 | 0.07 | **p <0.01** | **0.03** | 0.80 | **p <0.01** | 0.17 | 0.07 | 0.06 |
| Stat5 Y694 | **p <0.01** | **0.03** | 0.11 | 0.23 | **p <0.01** | **0.04** | 0.56 | **p <0.01** |
| Stat6 Y641 | **p <0.01** | 0.06 | 0.13 | 0.17 | **p <0.01** | **0.03** | 0.31 | **p <0.01** |
| Survivin | **0.02** | 0.06 | 0.36 | 0.76 | 0.09 | 0.13 | **0.02** | 0.91 |
| Syk Y525/526 | **p <0.01** | **p <0.01** | **p <0.01** | 0.11 | 0.30 | **p <0.01** | 0.13 | **p <0.01** |
| TGF-Beta | 0.15 | **p <0.01** | 0.16 | 0.14 | **p <0.01** | 0.79 | **0.03** | 0.11 |
| TNF alpha | 0.06 | 0.24 | **0.05** | **0.02** | 0.31 | **p <0.01** | **0.03** | 0.11 |
| TNF-R1 | **p <0.01** | **0.02** | **p <0.01** | **0.04** | **0.01** | **0.03** | **0.02** | 0.20 |
| Tubulin alpha | **p <0.01** | 0.27 | **0.03** | **p <0.01** | **p <0.01** | 0.52 | 0.11 | **0.03** |
| Tyk2 | **0.04** | **p <0.01** | **0.05** | **p <0.01** | **p <0.01** | **p <0.01** | 0.13 | 0.10 |
| VASP S157 | 0.08 | **p <0.01** | **0.01** | **0.02** | 0.70 | 0.10 | 0.70 | **0.01** |
| XIAP | **0.01** | **p <0.01** | **p <0.01** | **p <0.01** | **p <0.01** | 0.08 | **p <0.01** | 0.24 |
| YAP S127 | **p <0.01** | **0.01** | **0.04** | **p <0.01** | **p <0.01** | **0.05** | **p <0.01** | **p <0.01** |

**Table 2.** Number of endpoints statistically different across cell lines for the three DMSO concentrations.

| Cell line | C1 | C2 | C3 |
|---|---|---|---|
| A549 | 95 | 144 | 140 |
| Calu-3 | 107 | 143 | 138 |
| H1734 | 82 | 91 | 119 |
| H1838 | 120 | 128 | 88 |
| H2122 | 136 | 134 | 135 |
| H23 | 95 | 99 | 104 |
| H358 | 85 | 128 | 109 |
| H522 | 99 | 103 | 117 |

**Table 3.** List of pathways enriched for each cell line based on the Reactome Knowledgebase software analysis.

| Cell line | Pathway name | Entities found | Entities ratio | Entities p-value | FDR | Reactions found | Reactions ratio |
|---|---|---|---|---|---|---|---|
| A549 | Diseases of signal transduction by growth factor receptors and second messengers | 27/416 | 0.036 | 1.11E-16 | 2.31E-14 | 239/448 | 0.034 |
| A549 | Signaling by Interleukins | 35/456 | 0.04 | 1.11E-16 | 2.31E-14 | 258/493 | 0.037 |
| A549 | Signaling by Receptor Tyrosine Kinases | 30/523 | 0.046 | 1.11E-16 | 2.31E-14 | 366/704 | 0.053 |
| A549 | Cytokine Signaling in Immune system | 39/795 | 0.07 | 1.11E-16 | 2.31E-14 | 315/687 | 0.052 |
| A549 | Signal Transduction | 67/2,854 | 0.25 | 1.11E-16 | 2.31E-14 | 771/2,408 | 0.182 |
| A549 | Signaling by NTRKs | 16/139 | 0.012 | 1.73E-14 | 3.00E-12 | 59/164 | 0.012 |
| A549 | Immune System | 50/2,246 | 0.196 | 2.92E-14 | 4.32E-12 | 455/1,593 | 0.12 |
| A549 | Extra-nuclear estrogen signaling | 13/80 | 0.007 | 8.23E-14 | 1.07E-11 | 21/38 | 0.003 |
| A549 | PI3K/AKT Signaling in Cancer | 14/116 | 0.01 | 4.57E-13 | 5.25E-11 | 20/21 | 0.002 |
| A549 | Intracellular signaling by second messengers | 20/319 | 0.028 | 5.04E-13 | 5.25E-11 | 52 /114 | 0.009 |
| A549 | PIP3 activates AKT signaling | 18/279 | 0.024 | 5.42E-12 | 5.09E-10 | 46/86 | 0.007 |
| A549 | Disease | 43/1,939 | 0.17 | 8.83E-12 | 7.33E-10 | 322/1,589 | 0.12 |
| A549 | Signaling by NTRK1 (TRKA) | 13/117 | 0.01 | 9.16E-12 | 7.33E-10 | 42 /102 | 0.008 |
| A549 | MAPK family signaling cascades | 19/338 | 0.03 | 1.36E-11 | 1.00E-09 | 48 /122 | 0.009 |
| A549 | Intrinsic Pathway for Apoptosis | 10/53 | 0.005 | 1.66E-11 | 1.15E-09 | 25 /62 | 0.005 |
| A549 | Estrogen-dependent nuclear events downstream of ESR-membrane signaling | 24/08 | 0.002 | 2.28E-11 | 1.48E-09 | 11/12 | 9.07E-04 |
| A549 | Signaling by HER2 | 10/56 | 0.005 | 2.83E-11 | 1.73E-09 | 40/46 | 0.003 |
| A549 | ESR-mediated signaling | 15/196 | 0.017 | 3.73E-11 | 2.13E-09 | 36 /110 | 0.008 |
| A549 | Shc1 events in HER2 signaling | 28/08 | 0.002 | 7.64E-11 | 4.13E-09 | 6/6 | 4.54E-4 |
| A549 | Interleukin-4 and Interleukin-13 Signaling | 12/111 | 0.01 | 8.30E-11 | 4.32E-09 | 29/47 | 0.004 |
| A549 | RAF/MAP kinase cascade | 17/292 | 0.026 | 1.09E-10 | 5.28E-09 | 36 /75 | 0.006 |
| A549 | Developmental Biology | 31/1,110 | 0.097 | 1.12E-10 | 5.28E-09 | 153/556 | 0.042 |
| A549 | MAPK1/MAPK3 signaling | 17/299 | 0.026 | 1.57E-10 | 7.06E-09 | 43/82 | 0.006 |
| A549 | Platelet activation, signaling and Aggregation | 16/265 | 0.023 | 2.45E-10 | 1.05E-08 | 48/115 | 0.009 |
| A549 | Signalling to ERKs | 08/36 | 0.003 | 5.43E-10 | 2.22E-08 | 17/32 | 0.002 |
| Calu-3 | PI3K/AKT Signaling in Cancer | 20/116 | 0.01 | 1.11E-16 | 1.31E-14 | 20/21 | 0.002 |
| Calu-3 | Extra-nuclear estrogen signaling | 18/80 | 0.007 | 1.11E-16 | 1.31E-14 | 35/38 | 0.003 |
| Calu-3 | Signaling by Receptor Tyrosine Kinases | 42/523 | 0.046 | 1.11E-16 | 1.31E-14 | 426/704 | 0.053 |
| Calu-3 | Diseases of signal transduction by growth factor receptors and second messengers | 40/416 | 0.036 | 1.11E-16 | 1.31E-14 | 260/448 | 0.034 |
| Calu-3 | PIP3 activates AKT signaling | 25/279 | 0.024 | 1.11E-16 | 1.31E-14 | 50/86 | 0.007 |

| | | | | | | | |
|---|---|---|---|---|---|---|---|
| Calu-3 | Intracellular signaling by second messengers | 27/319 | 0.028 | 1.11E-16 | 1.31E-14 | 57/114 | 0.009 |
| Calu-3 | Signal Transduction | 81/2,854 | 0.25 | 1.11E-16 | 1.31E-14 | 902/2,408 | 0.182 |
| Calu-3 | Signaling by Interleukins | 32/456 | 0.04 | 1.11E-16 | 1.31E-14 | 162/493 | 0.037 |
| Calu-3 | Cytokine Signaling in Immune system | 38/795 | 0.07 | 1.11E-16 | 1.31E-14 | 217/687 | 0.052 |
| Calu-3 | Immune System | 56/2,246 | 0.196 | 3.44E-15 | 3.62E-13 | 373/1,593 | 0.12 |
| Calu-3 | Disease | 52/1,939 | 0.17 | 3.77E-15 | 3.62E-13 | 329/1,589 | 0.12 |
| Calu-3 | Signaling by NTRKs | 17/139 | 0.012 | 9.88E-15 | 8.70E-13 | 69/164 | 0.012 |
| Calu-3 | ESR-mediated signaling | 19/196 | 0.017 | 1.43E-14 | 1.16E-12 | 97/110 | 0.008 |
| Calu-3 | VEGFA-VEGFR2 Pathway | 15/98 | 0.009 | 1.71E-14 | 1.30E-12 | 46/79 | 0.006 |
| Calu-3 | Negative regulation of the PI3K/AKT network | 16/125 | 0.011 | 3.23E-14 | 2.29E-12 | 08/10 | 7.56E-4 |
| Calu-3 | Signaling by VEGF | 15/108 | 0.009 | 6.82E-14 | 4.50E-12 | 46/86 | 0.007 |
| Calu-3 | Intrinsic Pathway for Apoptosis | 12/53 | 0.005 | 9.02E-14 | 5.59E-12 | 39/62 | 0.005 |
| Calu-3 | PI5P, PP2A and IER3 Regulate PI3K/AKT Signaling | 15/118 | 0.01 | 2.40E-13 | 1.41E-11 | 5/7 | 5.29E-4 |
| Calu-3 | MAPK family signaling cascades | 22/338 | 0.03 | 2.73E-13 | 1.53E-11 | 43/122 | 0.009 |
| Calu-3 | RAF/MAP kinase cascade | 20/292 | 0.026 | 1.54E-12 | 8.15E-11 | 37/75 | 0.006 |
| Calu-3 | MAPK1/MAPK3 signaling | 20/299 | 0.026 | 2.35E-12 | 1.18E-10 | 41/82 | 0.006 |
| Calu-3 | Programmed Cell Death | 17/198 | 0.017 | 2.67E-12 | 1.28E-10 | 85/175 | 0.013 |
| Calu-3 | Constitutive Signaling by AKT1 E17K in Cancer | 09/26 | 0.002 | 3.07E-12 | 1.41E-10 | 18/18 | 0.001 |
| Calu-3 | Signaling by Nuclear Receptors | 19/273 | 0.024 | 4.53E-12 | 1.93E-10 | 97/191 | 0.014 |
| Calu-3 | Signaling by HER2 | 11/56 | 0.005 | 4.59E-12 | 1.93E-10 | 43/46 | 0.003 |
| H1734 | PI3K/AKT Signaling in Cancer | 17/116 | 0.01 | 1.11E-16 | 9.44E-15 | 19 /21 | 0.002 |
| H1734 | ESR-mediated signaling | 20/196 | 0.017 | 1.11E-16 | 9.44E-15 | 92 /110 | 0.008 |
| H1734 | Extra-nuclear estrogen signaling | 17/80 | 0.007 | 1.11E-16 | 9.44E-15 | 30/38 | 0.003 |
| H1734 | Signaling by Receptor Tyrosine Kinases | 36/523 | 0.046 | 1.11E-16 | 9.44E-15 | 364/704 | 0.053 |
| H1734 | Signaling by Nuclear Receptors | 21/273 | 0.024 | 1.11E-16 | 9.44E-15 | 94/191 | 0.014 |
| H1734 | Diseases of signal transduction by growth factor receptors and second messengers | 29/416 | 0.036 | 1.11E-16 | 9.44E-15 | 197/448 | 0.034 |
| H1734 | PIP3 activates AKT signaling | 23/279 | 0.024 | 1.11E-16 | 9.44E-15 | 30/86 | 0.007 |
| H1734 | Signaling by Interleukins | 30/456 | 0.04 | 1.11E-16 | 9.44E-15 | 165/493 | 0.037 |
| H1734 | Signal Transduction | 63/2,854 | 0.25 | 1.11E-16 | 9.44E-15 | 751/2408 | 0.182 |
| H1734 | Cytokine Signaling in Immune system | 33/795 | 0.07 | 1.11E-16 | 9.44E-15 | 205/687 | 0.052 |
| H1734 | Intracellular signaling by second messengers | 23/319 | 0.028 | 1.11E-16 | 9.44E-15 | 33/115 | 0.009 |
| H1734 | Signaling by NTRKs | 16/139 | 0.012 | 1.55E-15 | 1.21E-13 | 54/164 | 0.012 |
| H1734 | Signaling by NTRK1 (TRKA) | 13/117 | 0.01 | 1.34E-12 | 9.66E-11 | 42/102 | 0.008 |
| H1734 | PI5P, PP2A and IER3 Regulate PI3K/AKT Signaling | 13/118 | 0.01 | 1.49E-12 | 9.99E-11 | 5/7 | 5.29E-4 |
| H1734 | Negative regulation of the PI3K/AKT network | 13/125 | 0.011 | 3.04E-12 | 1.89E-10 | 7/10 | 7.56E-4 |

| | | | | | | | |
|---|---|---|---|---|---|---|---|
| H1734 | Estrogen-dependent nuclear events downstream of ESR-membrane signaling | 08/24 | 0.002 | 6.96E-12 | 4.10E-10 | 11/12 | 9.07E-4 |
| H1734 | Disease | 39/1,939 | 0.17 | 1.26E-11 | 6.65E-10 | 255/1589 | 0.12 |
| H1734 | Constitutive Signaling by AKT1 E17K in Cancer | 08/26 | 0.002 | 1.31E-11 | 6.65E-10 | 18/18 | 0.001 |
| H1734 | MAP kinase activation | 10/63 | 0.006 | 2.02E-11 | 6.65E-10 | 20/32 | 0.002 |
| H1734 | MAPK targets/ Nuclear events mediated by MAP kinases | 08/31 | 0.003 | 5.19E-11 | 1.23E-09 | 13/16 | 0.001 |
| H1734 | Immune System | 41/2,246 | 0.196 | 5.84E-11 | 1.23E-09 | 312/1593 | 0.12 |
| H1734 | MyD88:MAL(TIRAP) cascade initiated on plasma membrane | 11/96 | 0.008 | 5.85E-11 | 1.23E-09 | 21/64 | 0.005 |
| H1734 | Toll Like Receptor TLR6:TLR2 Cascade | 11/96 | 0.008 | 5.85E-11 | 1.23E-09 | 21/66 | 0.005 |
| H1734 | Interleukin-17 signaling | 10/71 | 0.006 | 6.39E-11 | 1.31E-09 | 20/35 | 0.003 |
| H1734 | TRIF(TICAM1)-mediated TLR4 signaling | 11/97 | 0.008 | 6.53E-11 | 1.31E-09 | 21/58 | 0.004 |
| H1838 | PI3K/AKT Signaling in Cancer | 22/116 | 0.01 | 1.11E-16 | 1.39E-14 | 20/21 | 0.002 |
| H1838 | Signaling by Receptor Tyrosine Kinases | 47/523 | 0.046 | 1.11E-16 | 1.39E-14 | 430/704 | 0.053 |
| H1838 | PIP3 activates AKT signaling | 27/279 | 0.024 | 1.11E-16 | 1.39E-14 | 50/86 | 0.007 |
| H1838 | Diseases of signal transduction by growth factor receptors and second messengers | 38/416 | 0.036 | 1.11E-16 | 1.39E-14 | 252/448 | 0.034 |
| H1838 | Intracellular signaling by second messengers | 30/319 | 0.028 | 1.11E-16 | 1.39E-14 | 59 /114 | 0.009 |
| H1838 | Signaling by NTRKs | 20/139 | 0.012 | 1.11E-16 | 1.39E-14 | 68/164 | 0.012 |
| H1838 | Signal Transduction | 90/2,854 | 0.25 | 1.11E-16 | 1.39E-14 | 888/2,408 | 0.182 |
| H1838 | Signaling by Interleukins | 34/456 | 0.04 | 1.11E-16 | 1.39E-14 | 175/493 | 0.037 |
| H1838 | Cytokine Signaling in Immune system | 39/795 | 0.07 | 1.11E-16 | 1.39E-14 | 220/687 | 0.052 |
| H1838 | Intrinsic Pathway for Apoptosis | 14/53 | 0.005 | 2.22E-16 | 2.49E-14 | 44/62 | 0.005 |
| H1838 | Disease | 55/1,939 | 0.17 | 1.11E-15 | 1.13E-13 | 340/1,589 | 0.12 |
| H1838 | MAPK family signaling cascades | 25/338 | 0.03 | 1.44E-15 | 1.34E-13 | 55/122 | 0.009 |
| H1838 | Extra-nuclear estrogen signaling | 15/80 | 0.007 | 2.55E-15 | 2.13E-13 | 28/38 | 0.003 |
| H1838 | Constitutive Signaling by AKT1 E17K in Cancer | 11/26 | 0.002 | 2.66E-15 | 2.13E-13 | 18/18 | 0.001 |
| H1838 | Apoptosis | 19/180 | 0.016 | 1.14E-14 | 8.58E-13 | 104/141 | 0.011 |
| H1838 | Immune System | 57/2,246 | 0.196 | 3.32E-14 | 2.32E-12 | 414/1,593 | 0.12 |
| H1838 | VEGFA-VEGFR2 Pathway | 15/98 | 0.009 | 4.70E-14 | 3.10E-12 | 50/79 | 0.006 |
| H1838 | Programmed Cell Death | 19/198 | 0.017 | 6.08E-14 | 3.77E-12 | 109/175 | 0.013 |
| H1838 | Signaling by VEGF | 15/108 | 0.009 | 1.87E-13 | 1.10E-11 | 50/86 | 0.007 |
| H1838 | Signaling by NTRK1 (TRKA) | 15/117 | 0.01 | 5.78E-13 | 3.24E-11 | 49/102 | 0.008 |
| H1838 | Negative regulation of the PI3K/AKT network | 15/125 | 0.011 | 1.47E-12 | 7.77E-11 | 8/10 | 7.56E-4 |
| H1838 | Developmental Biology | 37/1,110 | 0.097 | 4.56E-12 | 2.32E-10 | 163/556 | 0.042 |
| H1838 | Nervous system development | 27/584 | 0.051 | 5.91E-12 | 2.84E-10 | 99/324 | 0.024 |
| H1838 | RHO GTPase Effectors | 20/295 | 0.026 | 6.72E-12 | 3.09E-10 | 46/113 | 0.009 |

| | | | | | | | |
|---|---|---|---|---|---|---|---|
| H1838 | ESR-mediated signaling | 17/196 | 0.017 | 6.92E-12 | 3.12E-10 | 43/110 | 0.008 |
| H2122 | Constitutive Signaling by AKT1 E17K in Cancer | 12/26 | 0.002 | 1.11E-16 | 1.24E-14 | 18/18 | 0.001 |
| H2122 | PI3K/AKT Signaling in Cancer | 21/116 | 0.01 | 1.11E-16 | 1.24E-14 | 20/21 | 0.002 |
| H2122 | Extra-nuclear estrogen signaling | 17/80 | 0.007 | 1.11E-16 | 1.24E-14 | 28/38 | 0.003 |
| H2122 | Signaling by Interleukins | 41/456 | 0.04 | 1.11E-16 | 1.24E-14 | 273/493 | 0.037 |
| H2122 | Signaling by Receptor Tyrosine Kinases | 43/523 | 0.046 | 1.11E-16 | 1.24E-14 | 374/704 | 0.053 |
| H2122 | Intracellular signaling by second messengers | 28/319 | 0.028 | 1.11E-16 | 1.24E-14 | 59/114 | 0.009 |
| H2122 | Cytokine Signaling in Immune system | 48/795 | 0.07 | 1.11E-16 | 1.24E-14 | 340/687 | 0.052 |
| H2122 | Diseases of signal transduction by growth factor receptors and second messengers | 40/416 | 0.036 | 1.11E-16 | 1.24E-14 | 213/448 | 0.034 |
| H2122 | Signal Transduction | 95/2,854 | 0.25 | 1.11E-16 | 1.24E-14 | 867/2,408 | 0.182 |
| H2122 | Immune System | 65/2,246 | 0.196 | 1.11E-16 | 1.24E-14 | 529/1,593 | 0.12 |
| H2122 | Interleukin-4 and Interleukin-13 signaling | 18/111 | 0.01 | 2.22E-16 | 2.07E-14 | 29/47 | 0.004 |
| H2122 | PIP3 activates AKT signaling | 25/279 | 0.024 | 2.22E-16 | 2.07E-14 | 50/86 | 0.007 |
| H2122 | Intrinsic Pathway for Apoptosis | 14/53 | 0.005 | 7.77E-16 | 6.68E-14 | 43/62 | 0.005 |
| H2122 | Disease | 57/1,939 | 0.17 | 6.44E-15 | 5.15E-13 | 298/1,589 | 0.12 |
| H2122 | ESR-mediated signaling | 19/196 | 0.017 | 2.66E-13 | 1.99E-11 | 46 /110 | 0.008 |
| H2122 | Programmed Cell Death | 19/198 | 0.017 | 3.17E-13 | 2.22E-11 | 102/175 | 0.013 |
| H2122 | Apoptosis | 18/180 | 0.016 | 7.13E-13 | 4.71E-11 | 96/141 | 0.011 |
| H2122 | MAPK family signaling cascades | 23/338 | 0.03 | 9.26E-13 | 5.48E-11 | 55/122 | 0.009 |
| H2122 | MTOR signalling | 11/41 | 0.004 | 9.29E-13 | 5.48E-11 | 20/29 | 0.002 |
| H2122 | Signaling by NTRKs | 16/139 | 0.012 | 1.87E-12 | 1.05E-10 | 63/164 | 0.012 |
| H2122 | Generic Transcription Pathway | 42/1,258 | 0.11 | 2.43E-12 | 1.29E-10 | 402/824 | 0.062 |
| H2122 | VEGFA-VEGFR2 Pathway | 14/98 | 0.009 | 2.93E-12 | 1.50E-10 | 49/79 | 0.006 |
| H2122 | Estrogen-dependent nuclear events downstream of ESR-membrane signaling | 09/24 | 0.002 | 6.20E-12 | 2.98E-10 | 12/12 | 9.07E-4 |
| H2122 | Signaling by VEGF | 14/108 | 0.009 | 1.05E-11 | 4.82E-10 | 49/86 | 0.007 |
| H2122 | Developmental Biology | 38/1,110 | 0.097 | 1.94E-11 | 8.73E-10 | 176/556 | 0.042 |
| H23 | Extra-nuclear estrogen signaling | 17/80 | 0.007 | 1.11E-16 | 1.32E-14 | 28/38 | 0.003 |
| H23 | Signaling by Receptor Tyrosine Kinases | 38/523 | 0.046 | 1.11E-16 | 1.32E-14 | 390/704 | 0.053 |
| H23 | Signaling by Interleukins | 33/456 | 0.04 | 1.11E-16 | 1.32E-14 | 240/493 | 0.037 |
| H23 | Diseases of signal transduction by growth factor receptors and second messengers | 28/416 | 0.036 | 1.11E-16 | 1.32E-14 | 201/448 | 0.034 |
| H23 | ESR-mediated signaling | 20/196 | 0.017 | 1.11E-16 | 1.32E-14 | 46/110 | 0.008 |
| H23 | Signaling by NTRKs | 19/139 | 0.012 | 1.11E-16 | 1.32E-14 | 68/164 | 0.012 |
| H23 | Cytokine Signaling in Immune system | 37/795 | 0.07 | 1.11E-16 | 1.32E-14 | 281/687 | 0.052 |
| H23 | Signal Transduction | 72/2,854 | 0.25 | 1.11E-16 | 1.32E-14 | 726/2,408 | 0.182 |
| H23 | PI3K/AKT Signaling in Cancer | 16/116 | 0.01 | 5.55E-16 | 5.88E-14 | 19/21 | 0.002 |

| | | | | | | | |
|---|---|---|---|---|---|---|---|
| H23 | PIP3 activates AKT signaling | 21/279 | 0.024 | 1.33E-15 | 1.24E-13 | 33/86 | 0.007 |
| H23 | Intracellular signaling by second messengers | 22/319 | 0.028 | 1.44E-15 | 1.24E-13 | 37/114 | 0.009 |
| H23 | Signaling by Nuclear Receptors | 20/273 | 0.024 | 1.14E-14 | 9.03E-13 | 46/191 | 0.014 |
| H23 | Signaling by NTRK1 (TRKA) | 15/117 | 0.01 | 1.29E-14 | 9.40E-13 | 47/102 | 0.008 |
| H23 | Estrogen-dependent nuclear events downstream of ESR-membrane signaling | 09/24 | 0.002 | 2.81E-13 | 1.91E-11 | 12/12 | 9.07E-4 |
| H23 | Immune System | 47/2,246 | 0.196 | 3.79E-13 | 2.39E-11 | 442/1,593 | 0.12 |
| H23 | TRIF(TICAM1)-mediated TLR4 signaling | 13/97 | 0.008 | 4.92E-13 | 2.75E-11 | 26/58 | 0.004 |
| H23 | MyD88-independent TLR4 cascade | 13/97 | 0.008 | 4.92E-13 | 2.75E-11 | 26/60 | 0.005 |
| H23 | MAPK family signaling cascades | 20/338 | 0.03 | 5.69E-13 | 2.86E-11 | 50/122 | 0.009 |
| H23 | Constitutive Signaling by AKT1 E17K in Cancer | 09/26 | 0.002 | 5.72E-13 | 2.86E-11 | 18/18 | 0.001 |
| H23 | Toll Like Receptor 4 (TLR4) Cascade | 14/129 | 0.011 | 9.68E-13 | 4.55E-11 | 27/95 | 0.007 |
| H23 | Disease | 43/1,939 | 0.17 | 1.21E-12 | 5.46E-11 | 275/1,589 | 0.12 |
| H23 | Axon guidance | 24/558 | 0.049 | 1.60E-12 | 6.87E-11 | 95/298 | 0.023 |
| H23 | Nervous system development | 24/584 | 0.051 | 4.15E-12 | 1.70E-10 | 95/324 | 0.024 |
| H23 | Toll Like Receptor 3 (TLR3) Cascade | 12/96 | 0.008 | 6.35E-12 | 2.48E-10 | 25/61 | 0.005 |
| H23 | MyD88:MAL(TIRAP) cascade initiated on plasma membrane | 12/96 | 0.008 | 9.13E-12 | 3.10E-10 | 24/64 | 0.005 |
| H358 | Extra-nuclear estrogen signaling | 15/80 | 0.007 | 1.11E-16 | 1.65E-14 | 36/38 | 0.003 |
| H358 | PI3K/AKT Signaling in Cancer | 20/116 | 0.01 | 1.11E-16 | 1.65E-14 | 19/21 | 0.002 |
| H358 | Signaling by Receptor Tyrosine Kinases | 33/523 | 0.046 | 1.11E-16 | 1.65E-14 | 376/704 | 0.053 |
| H358 | Diseases of signal transduction by growth factor receptors and second messengers | 31/416 | 0.036 | 1.11E-16 | 1.65E-14 | 235/448 | 0.034 |
| H358 | PIP3 activates AKT signaling | 23/279 | 0.024 | 1.11E-16 | 1.65E-14 | 30/86 | 0.007 |
| H358 | Signal Transduction | 66/2,854 | 0.25 | 1.11E-16 | 1.65E-14 | 818/2,408 | 0.182 |
| H358 | Intracellular signaling by second messengers | 25/319 | 0.028 | 1.11E-16 | 1.65E-14 | 38/114 | 0.009 |
| H358 | PI5P, PP2A and IER3 Regulate PI3K/AKT Signaling | 15/118 | 0.01 | 5.66E-15 | 7.42E-13 | 5/7 | 5.29E-4 |
| H358 | Negative regulation of the PI3K/AKT network | 15/125 | 0.011 | 1.29E-14 | 1.37E-12 | 7/10 | 7.56E-4 |
| H358 | Disease | 44/1,939 | 0.17 | 1.32E-14 | 1.37E-12 | 303/1,589 | 0.12 |
| H358 | Signaling by Interleukins | 23/456 | 0.04 | 4.86E-14 | 4.62E-12 | 246/493 | 0.037 |
| H358 | Developmental Biology | 33/1,110 | 0.097 | 1.15E-13 | 9.25E-12 | 156/556 | 0.042 |
| H358 | Nervous system development | 25/584 | 0.051 | 1.16E-13 | 9.25E-12 | 94/324 | 0.024 |
| H358 | VEGFA-VEGFR2 Pathway | 13/98 | 0.009 | 2.48E-13 | 1.83E-11 | 45/79 | 0.006 |

| | | | | | | | |
|---|---|---|---|---|---|---|---|
| H358 | Constitutive Signaling by AKT1 E17K in Cancer | 09/26 | 0.002 | 3.25E-13 | 2.24E-11 | 18/18 | 0.001 |
| H358 | Axon guidance | 24/558 | 0.049 | 3.68E-13 | 2.39E-11 | 92/298 | 0.023 |
| H358 | ESR-mediated signaling | 16/196 | 0.017 | 5.28E-13 | 3.22E-11 | 98/110 | 0.008 |
| H358 | Signaling by VEGF | 13/108 | 0.009 | 8.30E-13 | 4.81E-11 | 45/86 | 0.007 |
| H358 | MAPK family signaling cascades | 19/338 | 0.03 | 1.79E-12 | 9.85E-11 | 49/122 | 0.009 |
| H358 | Estrogen-dependent nuclear events downstream of ESR-membrane signaling | 8/24 | 0.002 | 9.53E-12 | 4.96E-10 | 11/12 | 9.07E-4 |
| H358 | Cytokine Signaling in Immune system | 26/795 | 0.07 | 1.44E-11 | 7.07E-10 | 302/687 | 0.052 |
| H358 | Signaling by NTRKs | 13/139 | 0.012 | 1.86E-11 | 8.75E-10 | 50/164 | 0.012 |
| H358 | Constitutive Signaling by Aberrant PI3K in Cancer | 11/89 | 0.008 | 4.05E-11 | 1.79E-09 | 1/2 | 1.51E-4 |
| H358 | Signaling by NTRK1 (TRKA) | 12/117 | 0.01 | 4.16E-11 | 1.79E-09 | 37/102 | 0.008 |
| H358 | Signaling by Nuclear Receptors | 16/273 | 0.024 | 7.02E-11 | 2.88E-09 | 98/191 | 0.014 |
| H522 | Extra-nuclear estrogen signaling | 19/80 | 0.007 | 1.11E-16 | 1.21E-14 | 37/38 | 0.003 |
| H522 | ESR-mediated signaling | 21/196 | 0.017 | 1.11E-16 | 1.21E-14 | 99/110 | 0.008 |
| H522 | PI3K/AKT Signaling in Cancer | 19/116 | 0.01 | 1.11E-16 | 1.21E-14 | 19/21 | 0.002 |
| H522 | Signaling by Receptor Tyrosine Kinases | 44/523 | 0.046 | 1.11E-16 | 1.21E-14 | 383/704 | 0.053 |
| H522 | Signaling by Interleukins | 33/456 | 0.04 | 1.11E-16 | 1.21E-14 | 218/493 | 0.037 |
| H522 | Diseases of signal transduction by growth factor receptors and second messengers | 30/416 | 0.036 | 1.11E-16 | 1.21E-14 | 186/448 | 0.034 |
| H522 | Cytokine Signaling in Immune system | 39/795 | 0.07 | 1.11E-16 | 1.21E-14 | 278/687 | 0.052 |
| H522 | PIP3 activates AKT signaling | 23/279 | 0.024 | 1.11E-16 | 1.21E-14 | 32/86 | 0.007 |
| H522 | Signal Transduction | 75/2,854 | 0.25 | 1.11E-16 | 1.21E-14 | 782/2,408 | 0.182 |
| H522 | Signaling by NTRKs | 18/139 | 0.012 | 2.22E-16 | 1.98E-14 | 60/164 | 0.012 |
| H522 | Intracellular signaling by second messengers | 24/319 | 0.028 | 2.22E-16 | 1.98E-14 | 37/114 | 0.009 |
| H522 | VEGFA-VEGFR2 Pathway | 15/98 | 0.009 | 6.66E-15 | 5.46E-13 | 51/79 | 0.006 |
| H522 | Signaling by Nuclear Receptors | 21/273 | 0.024 | 1.20E-14 | 8.99E-13 | 99/191 | 0.014 |
| H522 | Signaling by VEGF | 15/108 | 0.009 | 2.70E-14 | 1.89E-12 | 51/86 | 0.007 |
| H522 | Constitutive Signaling by AKT1 E17K in Cancer | 10/26 | 0.002 | 3.49E-14 | 2.27E-12 | 18/18 | 0.001 |
| H522 | MTOR signalling | 11/41 | 0.004 | 8.38E-14 | 5.11E-12 | 20/29 | 0.002 |
| H522 | Immune System | 51/2,246 | 0.196 | 3.20E-13 | 1.86E-11 | 422/1,593 | 0.12 |
| H522 | Estrogen-dependent nuclear events downstream of ESR-membrane signaling | 9/24 | 0.002 | 8.68E-13 | 4.69E-11 | 12/12 | 9.7E-4 |
| H522 | TRIF(TICAM1)-mediated TLR4 signaling | 13/97 | 0.008 | 2.46E-12 | 1.21E-10 | 25/58 | 0.004 |
| H522 | MyD88-independent TLR4 cascade | 13/97 | 0.008 | 2.46E-12 | 1.21E-10 | 25/60 | 0.005 |
| H522 | MAPK family signaling cascades | 20/338 | 0.03 | 6.41E-12 | 3.01E-10 | 51/122 | 0.009 |

| | | | | | | | |
|---|---|---|---|---|---|---|---|
| H522 | MyD88 cascade initiated on plasma membrane | 12/85 | 0.007 | 9.96E-12 | 8.80E-11 | 24/58 | 0.004 |
| H522 | Toll Like Receptor 10 (TLR10) Cascade | 12/85 | 0.007 | 9.96E-12 | 8.80E-11 | 24/59 | 0.004 |
| H522 | Toll Like Receptor 5 (TLR5) Cascade | 12/85 | 0.007 | 9.96E-12 | 8.80E-11 | 24/59 | 0.004 |
| H522 | Nervous system development | 25/584 | 0.051 | 1.07E-11 | 9.70E-11 | 101/324 | 0.024 |

**Table 4.** List of antibodies used for the RPPA analysis including target proteins, vendors, catalogue numbers, and dilutions.

| Antibody | Vendor | Catalog number | Dilution | Host |
|---|---|---|---|---|
| 4E-BP1 S65 | CellSig | 9451 | 1:50 | R |
| 4E-BP1 T70 | CellSig | 9455 | 1:200 | R |
| Acetyl-CoA Carboxylase S79 | CellSig | 3661 | 1:50 | R |
| AKT | CellSig | 9272 | 1:2000 | R |
| AKT S473 | CellSig | 9271 | 1:100 | R |
| Alk | CellSig | 3633 | 1:50 | R |
| Alk Y1604 | CellSig | 3341 | 1:50 | R |
| AMPK alpha T172 | CellSig | 4188 | 1:2000 | R |
| AMPK alpha1 S485 | CellSig | 4184 | 1:50 | R |
| AMPK beta1 S108 | CellSig | 4181 | 1:50 | R |
| Androgen Receptor S81 | Millipore | 07-1375 | 1:1000 | R |
| A-Raf S299 | CellSig | 4431 | 1:50 | R |
| ATF-2 T69/71 | CellSig | 9225 | 1:500 | R |
| ATG5 | CellSig | 2630 | 1:1000 | R |
| ATG12 | CellSig | 2010 | 1:100 | R |
| ATM S1981 | CellSig | 5883 | 1:50 | R |
| ATP-Citrate Lyase S454 | CellSig | 4331 | 1:100 | R |
| ATR S428 | CellSig | 2853 | 1:50 | R |
| Aurora A T288/Aurora B T232/AuroraC T198 | CellSig | 2914 | 1:50 | R |
| Axl Y702 | CellSig | 5724 | 1:50 | R |
| BAD S112 | CellSig | 9291 | 1:200 | R |
| BAD S136 | CellSig | 9295 | 1:50 | R |
| BAD S155 | CellSig | 9297 | 1:100 | R |
| Bak | CellSig | 3814 | 1:100 | R |
| Bax | CellSig | 2772 | 1:200 | R |
| Bcl-2 S70 | CellSig | 2827 | 1:50 | R |
| Bcl-2 T56 | CellSig | 2875 | 1:200 | R |
| Bcl-xL | CellSig | 2762 | 1:500 | R |
| Beclin1 | CellSig | 3738 | 1:100 | R |
| BIM | CellSig | 2933 | 1:500 | R |
| B-Raf S445 | CellSig | 2696 | 1:50 | R |
| c-Met | Abcam | ab51067 | 1:200 | R |
| c-Abl T735 | CellSig | 2864 | 1:50 | R |
| c-Abl Y245 | CellSig | 2861 | 1:100 | R |
| Caspase-3, cleaved (D175) | CellSig | 9661 | 1:50 | R |
| Caspase-6, cleaved (D162) | CellSig | 9761 | 1:50 | R |
| Caspase-7, cleaved (D198) | CellSig | 9491 | 1:100 | R |
| Caspase-9, cleaved (D330) | CellSig | 9501 | 1:50 | R |
| Catenin beta S33/37/T41 | CellSig | 9561 | 1:100 | R |
| Chk-1 S345 | CellSig | 2341 | 1:50 | R |
| Chk-2 S33/35 | CellSig | 2665 | 1:50 | R |
| Cofilin S3 | CellSig | 3313 | 1:500 | R |
| Cox-2 | BD | 610203 | 1:200 | M |
| cPLA2 S505 | CellSig | 2831 | 1:1000 | R |
| C-Raf S338 | CellSig | 9427 | 1:200 | R |
| CREB S133 | CellSig | 9191 | 1:100 | R |
| CrkL Y207 | CellSig | 3181 | 1:100 | R |
| Cyclin A2 | CellSig | 4656 | 1:50 | M |
| Cyclin B1 | CellSig | 4135 | 1:200 | M |
| Cyclin D1 | BD | 554180 | 1:100 | M |
| EGFR | CellSig | 2232 | 1:100 | R |
| EGFR Y1045 | CellSig | 2237 | 1:50 | R |
| EGFR Y1068 | CellSig | 2234 | 1:50 | R |
| EGFR Y1148 | BioSource | 44-792 | 1:100 | R |
| EGFR Y1173 | BioSource | 44-794 | 1:100 | R |
| eIF4E S209 | CellSig | 9741 | 1:50 | R |

| | | | | |
|---|---|---|---|---|
| eIF4G S1108 | CellSig | 2441 | 1:1000 | R |
| Elk-1 S383 | CellSig | 9181 | 1:100 | R |
| eNOS S113 | CellSig | 9575 | 1:50 | R |
| eNOS/NOS III S116 | Upstate | 07-357 | 1:500 | R |
| Ephrin A3 Y799/A4 Y799/A5 Y833 | Abcam | 124881 | 1:100 | R |
| ERK 1/2 | CellSig | 9102 | 1:200 | R |
| ERK 1/2 T202/Y204 | CellSig | 9101 | 1:1000 | R |
| Estrogen Receptor alpha | CellSig | 2511 | 1:1000 | M |
| Estrogen Receptor alpha S118 | CellSig | 2511 | 1:1000 | M |
| Etk Y40 | CellSig | 3211 | 1:2000 | R |
| Ezrin T567/Radixin T564/Moesin T558 | CellSig | 3141 | 1:100 | R |
| FADD S194 | CellSig | 2781 | 1:100 | R |
| FAK Y576/577 | CellSig | 3281 | 1:200 | R |
| FOXO1 T24/FOXO3 T32 | CellSig | 9464 | 1:200 | R |
| FOXO3 S253 | Upstate | 06-953 | 1:1000 | R |
| FOXM1 T600 | CellSig | 14655 | 1:100 | R |
| FOXO1 S256 | CellSig | 9461 | 1:100 | R |
| Grb2 | CellSig | 3972 | 1:1000 | R |
| GSK-3 alpha/beta S21/9 | CellSig | 9331 | 1:100 | R |
| HDAC 1 | CellSig | 2062 | 1:100 | R |
| HDAC 3 | CellSig | 2632 | 1:1000 | R |
| HDAC 4 | CellSig | 2072 | 1:100 | R |
| HDAC 6 | SantaCruz | sc-11420 | 1:2000 | R |
| HER2 | DAKO | A0485 | 1:250 | R |
| HER2 Y1248 | Imgenex | IMG-90189 | 1:500 | R |
| HER3 Y1197 | CellSig | 4561 | 1:100 | R |
| HER3 Y1289 | CellSig | 4791 | 1:200 | R |
| Histone H3, Acetyl Lys9 14 | CellSig | 9677 | 1:2000 | R |
| Histone H3, Di-Methyl Lys9 | CellSig | 9753 | 1:500 | R |
| Histone H3, Pan-Methyl Lys9 | CellSig | 4069 | 1:100 | R |
| Histone H3 S10 Mitosis Marker | Upstate | 06-570 | 1:200 | R |
| Histone H3 S28 | Upstate | 07-145 | 1:1000 | R |
| Histone H4 Acetyl Lys8 | CellSig | 2594 | 1:200 | R |
| HSP27 S82 | CellSig | 2406 | 1:100 | R |
| HSP90a T5/7 | CellSig | 3488 | 1:100 | R |
| IGF-1R Y1131/IR Y1146 | CellSig | 3021 | 1:500 | R |
| IGF-1R Y1135/1136 IR Y1150/1151 | CellSig | 3024 | 1:500 | R |
| IkappaB-alpha S32/36 | CellSig | 9246 | 1:100 | M |
| IL-8 | Abcam | ab7747 | 1:200 | R |
| IL-10 | Abcam | ab52909 | 1:2000 | R |
| IL-11 | SantaCruz | sc-7924 | 1:500 | R |
| Insulin Receptor beta | CellSig | 3025 | 1:200 | R |
| IRS-1 S612 | CellSig | 2386 | 1:200 | R |
| Jak1 Y1022/1023 | CellSig | 3331 | 1:50 | R |
| Ki67 | DAKO | M7240 | 1:100 | M |
| LC3B | CellSig | 2775 | 1:100 | R |
| Lck Y505 | Biosource | 44-850 | 1:50 | R |
| LIMK1 T508/LIMK2 T505 | CellSig | 3841 | 1:100 | R |
| LKB1 S334 | CellSig | 3055 | 1:50 | R |
| LKB1 S428 | CellSig | 3051 | 1:100 | R |
| MARCKS S152/156 | CellSig | 2741 | 1:200 | R |
| MDM2 S166 | CellSig | 3521 | 1:100 | R |
| MEK 1/2 | CellSig | 9122 | 1:500 | R |
| MEK 1/2 S217/221 | CellSig | 9121 | 1:200 | R |
| Met Y1234/1235 | CellSig | 3126 | 1:200 | R |
| MSK1 S360 | CellSig | 9594 | 1:50 | R |
| mTOR | CellSig | 2972 | 1:200 | R |
| mTOR S2448 | CellSig | 2971 | 1:100 | R |
| NF-kappaB p65 S536 | CellSig | 3031 | 1:100 | R |

| | | | | |
|---|---|---|---|---|
| p27 Kip1 | BD | 610242 | 1:100 | M |
| p27 T187 | Zymed | 71-7700 | 1:200 | R |
| p38 MAP Kinase T180/Y182 | CellSig | 9211 | 1:100 | R |
| p53 | CellSig | 9282 | 1:5000 | R |
| p53 S15 | CellSig | 9284 | 1:1000 | R |
| p62/SQSTM1 | CellSig | 8025 | 1:50 | R |
| p70 S6 Kinase S371 | CellSig | 9208 | 1:50 | R |
| p70 S6 Kinase T389 | CellSig | 9205 | 1:100 | R |
| p70 S6 Kinase T412 | Upstate | 07-018 | 1:500 | R |
| p90RSK S380 | CellSig | 9341 | 1:200 | R |
| p90RSK T359/S363 | CellSig | 9344 | 1:200 | R |
| PAK1 S199/204 PAK2 S192/197 | CellSig | 2605 | 1:50 | R |
| PAK1 T423/PAK2 T402 | CellSig | 2601 | 1:100 | R |
| PARP, cleaved (D214) | CellSig | 9541 | 1:100 | R |
| Paxillin Y118 | CellSig | 2541 | 1:500 | R |
| PDGF Receptor beta Y751 | CellSig | 3161 | 1:50 | R |
| PDK1 S241 | CellSig | 3061 | 1:200 | R |
| PDL1 E1L3N | CellSig | 13684 | 1:500 | R |
| PI3 Kinase p110gamma | CellSig | 4252 | 1:100 | R |
| PIAS1 | CellSig | 3550 | 1:100 | R |
| PKA C T197 | CellSig | 4781 | 1:200 | R |
| PKC a/BII T638/641 | CellSig | 9375 | 1:100 | R |
| PKC alpha S657 | Upstate | 06-822 | 1:1000 | R |
| PKC delta T505 | CellSig | 9374 | 1:50 | R |
| PKC theta T538 | CellSig | 9377 | 1:100 | R |
| PKC zeta/lambda T410/403 | CellSig | 9378 | 1:50 | R |
| PLCgamma1 Y783 | CellSig | 2821 | 1:100 | R |
| PLK1 T210 | BD | 558400 | 1:200 | M |
| PP2A a Subunit | CellSig | 2039 | 1:1000 | R |
| PP2A B Subunit | CellSig | 4953 | 1:1000 | R |
| PRAS40 T246 | BioSource | 44-1100 | 1:1000 | R |
| PTEN | CellSig | 9552 | 1:50 | R |
| PTEN S380 | CellSig | 9551 | 1:500 | R |
| Pyk2 Y402 | CellSig | 3291 | 1:200 | R |
| Raf S259 | CellSig | 9421 | 1:100 | R |
| Ras-GRF1 | CellSig | 3322 | 1:200 | R |
| Ras-GRF1 S916 | CellSig | 3321 | 1:50 | R |
| Rb S780 | CellSig | 3590 | 1:2000 | R |
| Ret Y905 | CellSig | 3221 | 1:100 | R |
| Ron Y1353 | Epitomics | 5176-1 | 1:1000 | R |
| RSK3 T356/S360 | CellSig | 9348 | 1:500 | R |
| S6 Ribosomal Protein S235/236 | CellSig | 4856 | 1:200 | R |
| S6 Ribosomal Protein S240/244 | CellSig | 2215 | 1:1000 | R |
| SAPK/JNK T183/Y185 | CellSig | 9251 | 1:100 | R |
| SEK1/MKK4 S80 | CellSig | 9155 | 1:50 | R |
| SGK1 S78 | CellSig | 5599 | 1:100 | R |
| Shc Y317 | Upstate | 07-206 | 1:200 | R |
| SHIP1 Y1020 | CellSig | 3941 | 1:50 | R |
| SHP2 Y580 | Biosource | 44-558 | 1:500 | R |
| Smad2 S245/250/255 | CellSig | 3104 | 1:100 | R |
| Smad2 S465/467 | CellSig | 3101 | 1:200 | R |
| SOCS1 | CellSig | 3950 | 1:50 | R |
| SOCS3 | CellSig | 2923 | 1:50 | R |
| Src Family Y416 | CellSig | 2101 | 1:100 | R |
| Src Y527 | CellSig | 2105 | 1:200 | R |
| Stat1 Y701 | CellSig | 9171 | 1:500 | R |
| Stat2 Y690 | CellSig | 4441 | 1:100 | R |
| Stat3 S727 | CellSig | 9134 | 1:100 | R |
| Stat3 Y705 | CellSig | 9145 | 1:100 | R |

| | | | | |
|---|---|---|---|---|
| Stat4 Y693 | CellSig | 5267 | 1:100 | R |
| Stat5 Y694 | CellSig | 9351 | 1:50 | R |
| Stat6 Y641 | CellSig | 9361 | 1:100 | R |
| Survivin | CellSig | 2808 | 1:500 | R |
| Syk Y525/526 | CellSig | 2711 | 1:50 | R |
| TGF-Beta | CellSig | 3709 | 1:1000 | R |
| TNF alpha | Abcam | ab9635 | 1:200 | R |
| TNF-R1 | CellSig | 3736 | 1:50 | R |
| Tubulin alpha | Sigma | T 6074 | 1:2000 | M |
| Tyk2 | CellSig | 9312 | 1:200 | R |
| VASP S157 | CellSig | 3111 | 1:100 | R |
| XIAP | CellSig | 2042 | 1:100 | R |
